# Phenotypic and Transcriptomic Analysis Revealed a Lack of Risk Perception by Native Tadpoles Toward Novel Non‐Native Fish

**DOI:** 10.1002/ece3.70481

**Published:** 2024-10-21

**Authors:** Yuanfei Wang, Yudong Zhu, Liuyang He, Haoqi Yu, Xiuqin Lin, Jianghong Ran, Feng Xie

**Affiliations:** ^1^ CAS Key Laboratory of Mountain Ecological Restoration and Bioresource Utilization and Ecological Restoration Biodiversity Conservation Key Laboratory of Sichuan Province, Chengdu Institute of Biology Chinese Academy of Sciences Chengdu China; ^2^ Key Laboratory of Bio‐Resource and Eco‐Environment of the Ministry of Education, College of Life Sciences Sichuan University Chengdu China; ^3^ University of Chinese Academy of Sciences Beijing China; ^4^ Sichuan Liziping National Nature Reserve Shimian China; ^5^ Open Laboratory of Shimian Research Center of Giant Panda Small Population Conservation and Rejuvenation Shimian China; ^6^ Liziping Giant Panda's Ecology and Conservation Observation and Research Station of Sichuan Province Shimian China

**Keywords:** kairomones, native fish, non‐native fish, prey naivety hypothesis, *Rana chaochiaoensis* tadpoles, transcriptomics

## Abstract

The introduction of alien species poses a serious threat to native biodiversity, and mountain lake systems in the southwest of China are particularly vulnerable to the introduction of non‐native fish. The prey naivety hypothesis states that native species may not be able to recognize novel introduced species due to a lack of common evolutionary background and therefore become easy targets, so the impacts of non‐native fish on mountain endemic amphibians need to be urgently assessed. In an ex‐situ experiment, we exposed the tadpoles of the Chaochiao Brown Frog (*Rana chaochiaoensis*), endemic to western China, to kairomones of both native and translocated fish species, and their phenotypic and genetic response patterns were compared. The results revealed significant phenotypic plasticity responses in total length (TOL), tail length (TL), and tail muscle width (TW) of tadpoles induced by native fish kairomone, while tadpoles exposed to translocated fish kairomone exhibited weaker phenotypic changes. At the transcriptional level, the number of differently expressed genes (DEGs) in the native fish treatment was 3.1‐fold (liver) and 52.6‐fold (tail muscle) higher than in the translocated fish treatment, respectively. There were more unique DEGs in the native fish treatment, primarily enriched in terms and pathways related to stress response, energy metabolism, and muscle development. The study revealed a lack of risk perception by native tadpoles toward novel non‐native fish, providing new evidence for the prey naivety hypothesis from both phenotypic and molecular perspectives. Future conservation efforts should prioritize assessing the impacts of non‐native fish on alpine and subalpine threatened and narrowly distributed amphibians. Additionally, prevention, early warning, monitoring, and removal of non‐native fish should be carried out as soon as possible.

## Graphical abstract

Our study reveals a lack of risk perception by native tadpoles toward novel alien fish and validates the prey naivety hypothesis, as well as providing new evidence for this hypothesis from phenotypic and molecular perspectives.

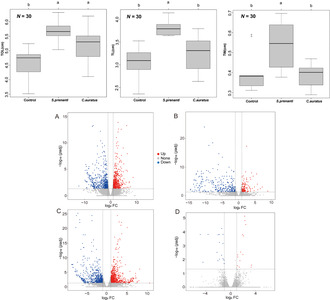

## 1 Introduction

With the development of global trade and transport, biological invasions caused by the introduction of alien species (Guo et al. 2024), the loss of plant and animal habitats (Brooks et al. 2002), and global climate change (Thomas et al. 2004) have become the world's three major environmental problems, seriously threatening global biodiversity, causing substantial economic losses globally, and affecting ecosystem function (Wiles et al. 2003). In aquatic ecosystems worldwide, the most frequently introduced aquatic animal taxon is fish, and non‐native freshwater fishes have been regarded as one of the major causes of the worldwide decline of aquatic fauna (Gozlan et al. 2010; Bernery et al. 2022). Non‐native fishes refer to fish that have been introduced to areas beyond their native range and are further categorized into alien fishes and translocated fishes considering the different origins. Alien fishes are fish that have been introduced from outside a region, and translocated fishes are fish that are native to the region but have been introduced to at least one other water body in the region (Wang et al. 2023). In recent years, more non‐native fish species have been recorded in alpine and subalpine regions, and the impact of these fish on regional ecosystems has become a hot topic.

Under the influence of human activities, some of the climatic and geographical barriers that could have kept out exotic species may have been weakened, and mountain ecosystems, which are rich in biodiversity, are also under ecological threat from the introduction of exotic species (Panda, Behera, and Roy 2018). The southwest mountains are a global hotspot for biodiversity conservation, and it is a concentrated distribution area of China's endemic amphibians, which are narrowly distributed and sensitive to environmental change (Xie et al. 2007). In the last two decades, fish have been introduced into mountain lake ecosystems by developers and local communities for religious releases, tourism, and aquaculture purposes, and some studies have linked non‐native fish introductions to native fish declines (Zhang et al. 2015; Zhu et al. 2022); however, the effects of introduced fish on the diversity of amphibians in mountain lakes and the responses of amphibians to exotic fish are not systematically studied.

Pheromones play an important role in the growth, development, and reproduction of organisms. According to the interaction relationship, pheromones are divided into intraspecific and interspecific pheromones, with interspecific pheromones including allomone, kairomone, and synomone (Cao 2022). Aquatic predators of both vertebrates and invertebrates release kairomone into the surrounding water, and prey can detect kairomone that triggers predator‐specific responses from prey to reduce vulnerability (Schoeppner and Relyea 2009). Fish metabolites, such as feces, release pheromones, mainly kairomone (Śluarczyk and Rygielska 2004), and many studies have investigated phenotypic induction experiments by using kairomone as pheromones (Nunes et al. 2014a), which prompts prey to develop better defended individuals and is very effective in inducing morphological changes (Grant and Bayly 1981; Rozenberg et al. 2015). Morphological (Kats and Ferrer 2003; Martín‐Torrijos et al. 2016), behavioral (Rick 2001), and life history (Nunes et al. 2014a) changes in response to fish kairomone have also been observed in a variety of aquatic organisms such as daphnia and amphibians. Under the induction of kairomone, the tissues of the organism undergo drastic morphological changes, and signal transduction is involved in the developmental process (Mori et al. 2005). Several studies have linked phenotypic plasticity induced by kairomone in salamanders to gene expression patterns (Matsunami et al. 2015, 2020). Recent studies have shown that endocrine regulation plays a key role in the expression of plasticity phenotypes in a variety of animals (Lema 2014; De Meyer et al. 2017).

The prey naivety hypothesis states that native species may not be able to recognize kairomones of novel introduced species due to a lack of common evolutionary background, fail to respond with effective avoidance behavior, and therefore become easy targets (Banks and Dickman 2007). Alpine and subalpine narrowly distributed amphibian species in the southwest mountains of China face many unknown novel non‐native fish predators and competitors (Zhu et al. 2022). It remains uncertain whether they will be able to recognize and respond to this threat.

The Chaochiao brown frog, *Rana chaochiaoensis*, is a widely distributed endemic species of alpine and subalpine mountains (1150–3500 m) in the southwest mountains of China, and its breeding and developmental habitats mainly include mountain lakes and marshes (Fei, Ye, and Jiang 2012). Its aquatic habitats contain the sympatric distribution of native Ya‐fish *Schizothorax prenanti* and translocated fish goldfish *Carassius auratus* (Guo et al. 2021). For the purposes of tourism and aquaculture, *C. auratus*, which typically inhabits lower elevation, has been translocated to the alpine and subalpine aquatic regions where the frog resides (Wang et al. 2023). These fish are omnivorous, with a diet primarily consisting of algae, aquatic vascular plants, and other small aquatic organisms, including anuran larvae (Ding 1994). In this study, the frog tadpoles were exposed to kairomones from native and translocated fish through an ex‐situ experiment. By combining phenotypic observation with modern omics analysis, we aim to test: (1) Do the frog tadpoles undergo phenotypical plasticity in response to kairomone induction from the two fish species? (2) the similarities and differences in their response patterns to kairomone induction from the two fish species, and (3) the differences in gene expression levels. This study may not only offer new support for the prey naivety hypothesis but also provide crucial guidance to relevant stakeholders on the conservation of threatened and narrowly distributed amphibians, as well as the management of non‐native fish in mountain lake ecosystems.

## 2 Materials and Methods

### 2.1 Sample Collection and Experimental Design

*Rana chaochiaoensis* tadpoles from one egg clutch were collected in June 2022 at Menghuo Castle (28.88666591° N, 102.35780125° E, 2639 m) in Shimian City, Sichuan Province, China. During the egg clutch collection process, we discovered that it was a single egg clutch containing less than average size, 1500 eggs (Fei, Ye, and Jiang 2012), with the same developmental stage. Therefore, we concluded that it should be a single egg clutch. The individuals from one egg clutch with the most identical genetic background are optimal for minimizing the genetic contribution to gene expression (Semenova et al. 2022). Then, we brought the egg clutch back to the laboratory, where they were incubated for approximately 2 weeks, with most of the tadpoles developing to Gosner stage 26 (Gosner 1960). Thirty tadpoles of similar body size and developmental period (Gosner stage 26) were selected and divided into three groups of 10 individuals each.

In this study, the frog tadpoles were exposed to different treatments in an experimental trial: aerated water and water with different fish kairomones. Therefore, following the method of Sih and Kats (1991), the water with kairomones from the rearing tanks of *S. prenanti* and *C. auratus* was used to raise the tadpoles of *R. chaochiaoensis*, respectively. The densities of each fish species were both 15 individuals, and the average sizes of each fish species were: *S. prenanti* = 7.2 ± 0.6 cm and *C. auratus* = 6.8 ± 0.5 cm. The water volumes of the tanks were both 7 L. Five liters of the rearing water from each of the two fish species tank was inoculated into the white container (42 × 30 × 10 cm) where the tadpoles lived, and the control group was inoculated with the same volume of aerated water. After each inoculation, the volume of water in the fish tank was replenished to its original volume. The treatment and control groups were replicated once each, and the tadpoles used for transcriptomic analysis were replicated three times from each group. Meanwhile, the tadpoles were fed with the solution of *Spirulina* powder once a day. Aerated water and water‐bearing fish kairomones were completely poured out and immediately inoculated with 5 L of fresh aerated water and water‐bearing fish kairomones, the process being repeated every 2 days. Treatments lasted approximately for 70 days until the tadpoles grew to Gosner stage 42 (Gosner 1960). All experimental procedures involving animals were strictly conducted in accordance with the regulations established by the Animal Care and Use Committee of the Chengdu Institute of Biology, Chinese Academy of Sciences (CIBDWLL2021014).

### 2.2 Morphological Observation

When tadpoles grew to Gosner stage 42 (Gosner 1960), all 10 tadpoles in each group were photographed under a stereomicroscope using a StereoZoom (Leica S8 Apo) with an attached digital camera (Leica DFC450 C) and Leica Application Suite X for morphological assessment. Using the NIH ImageJ public domain software (Schneider, Rasband, and Eliceiri 2012) and following the measurement methods described by Fei et al. (2005), three phenotypic traits were quantified for each tadpole, including total length (TOL), tail length (TL), and tail muscle width (TW). TOL represents the overall condition of the individual, and TL and TW represent functional traits associated with tadpole locomotion.

### 2.3 RNA Isolation, Sequencing, and Transcriptomic Analysis

To analyze differences in gene expression between treatments, we randomly sampled 2–3 tadpoles from each group. As the liver is crucial for various metabolic processes and the tail muscle is essential for tadpole locomotion (Gomes et al. 2009), we pooled the liver and tail muscle tissues after euthanizing using MS‐222 to extract the required total RNA, respectively. A total of six groups were generated for transcriptome sequencing (group *S. prenanti* treatment—Liver, *S. prenanti* treatment—Muscle, *C. auratus* treatment—Liver, *C. auratus* treatment—Muscle, and Control—Muscle samples = 3; group Control—Liver samples = 2). Total RNA was extracted using TRIzol reagent (Invitrogen, Carlsbad, CA, USA) according to the manufacturer's protocol. RNA degradation and contamination were tested using electrophoresis. After purification, the concentration and integrity of RNA were quantified, and equal amounts of total RNA from the six groups were used to construct the corresponding cDNA libraries. Sequencing was performed on an Illumina HiSeq 2500 platform from Annoroad (Beijing), and paired‐end reads were generated.

After removing the adapter reads, poly‐N, and low‐quality reads, the index files were constructed using Hisat2 software (Pertea et al. 2016), and the reads data were aligned to the reference genome of a related species, the European common frog (*Rana temporaria*). The expression levels of the genes were quantified using the quantitative expression software Stringtie (Pertea et al. 2016). The reads count was analyzed using DESeq2 software (Love, Huber, and Anders 2014) based on the negative binomial distribution, and differently expressed genes (DEGs) were identified using the criteria of *p* < 0.05 and |log_2_FC| ≥ 1. The gene ontology (GO) enrichment and pathway enrichment analysis of DEGs were conducted using KOBAS 3.0 with a *p*‐value threshold of 0.05 (Xie et al. 2011).

### 2.4 Statistical Analysis

A one‐way ANOVA was conducted to assess for the effects of kairomone exposure on morphological traits including TOL, TL, and TW. We ran a one‐way ANOVA using the aov() function, and then we used the “multcomp” package to perform a Tukey's post hoc test. Normal distribution and homogeneity of variance were assessed using the QQ plot and Bartlett tests, respectively. All statistical analyses were conducted in R version 4.1.1 (R Core Team 2020), with a significance level of *p* < 0.05.

## 3 Results

### 3.1 Morphological Trait Variation

**FIGURE 1:**
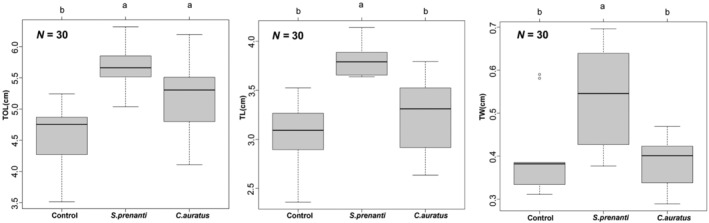
Box plots of the phenotypic response of *Rana chaochiaoensis* tadpoles under different treatments (median ± SD). Different letters indicate the significant differences (*p* < 0.05).

All phenotypic traits complied with normal distribution (Figure S1) and homogeneity of variance (Table S1). One‐way ANOVA indicated a significant increase in TOL (*p* < 0.001), TL (*p* < 0.001), and TW (*p* = 0.007) of *R. chaochiaoensis* tadpoles exposed to *S. prenanti* kairomone compared to the control. While the locomotion‐related phenotypes TL (*p* = 0.29) and TW (*p* = 0.94) of *R. chaochiaoensis* did not change significantly, only TOL (*p* = 0.027) increased significantly when exposed to *C. auratus* kairomone. When comparing between treatments, the tadpoles in the *S. prenanti* treatment group showed a significant increase in TL (*p* = 0.001) and TW (*p* = 0.003) compared to the *C. auratus* treatment group, while the TOL (*p* = 0.08) did not show a significant difference (Figure 1).

### 3.2 Identification of Differently Expressed Genes

The total number of reads obtained from sequencing each sample ranged from 40,656,128 to 49,763,008, and the quality percentage of Q30 bases was greater than 93.56%, and the quality scores of all sample bases were greater than 36, and the proportion of GC bases was greater than 45.8% (Table S2). The results indicated that the sequencing quality of all samples was qualified and could be used for subsequent analyses.

**FIGURE 2:**
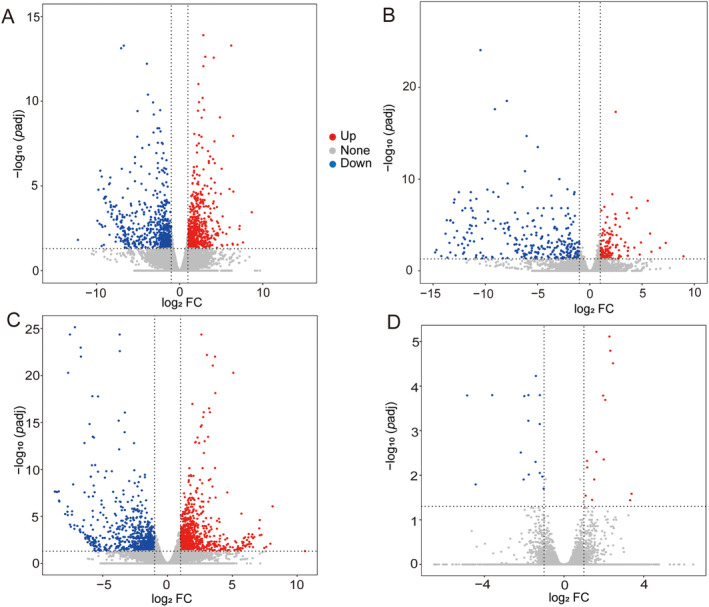
Analysis of DEGs in different tissues and treatments. Volcano plot of *Schizothorax prenanti* treatment vs. control in liver tissue (A); Volcano plot of *Carassius auratus* treatment vs. control in liver tissue (B); Volcano plot of *S. prenanti* treatment vs. control in tail muscle tissue (C); Volcano plot of *C. auratus* treatment vs. control in tail muscle tissue (D).

**FIGURE 3:**
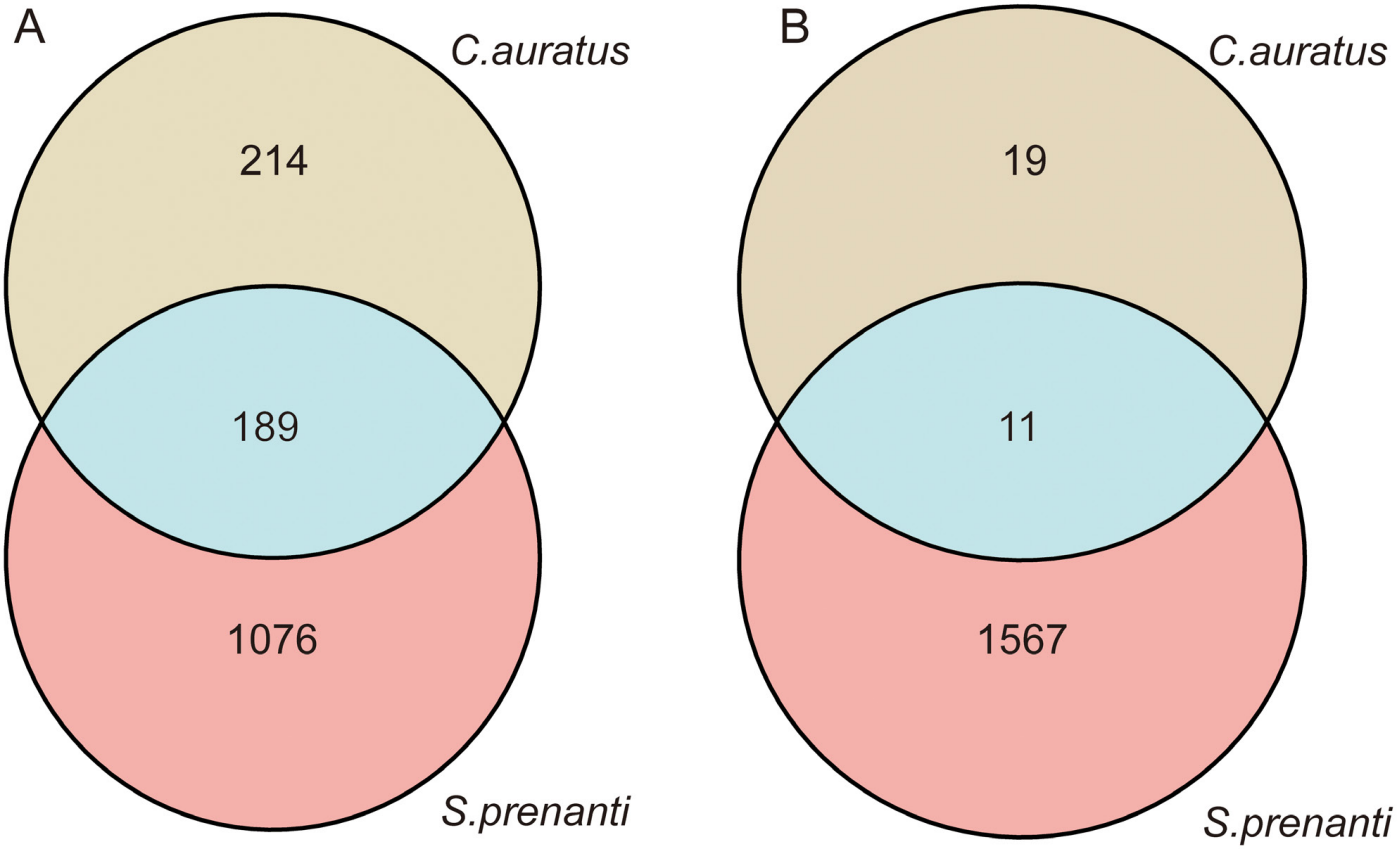
Venn diagram showing the number of DEGs identified from each treatment. Shared DEGs and DEGs uniquely identified in liver tissue (A); Shared DEGs and DEGs uniquely identified in tail muscle tissue (B).

Comparative transcriptomics was conducted to explore the molecular changes in liver and tail muscle of *R. chaochiaoensis* induced by different kairomone exposures. In liver tissue, a total of 1265 and 403 DEGs were identified in the “*S. prenanti* treatment vs. Control” and “*C. auratus* treatment vs. Control” comparisons, respectively (Figure 2A,B); among these, 189 DEGs were common in both comparisons. In addition, 1076 DEGs were uniquely identified in the “*S. prenanti* treatment vs. Control” comparison, and 214 DEGs were uniquely identified in the “*C. auratus* treatment vs. Control” comparison (Figure 3A).

In tail muscle tissue, a total of 1578 and 30 DEGs were identified in the “*S. prenanti* treatment vs. Control” and “*C. auratus* treatment vs. Control” comparisons, respectively (Figure 2C,D); among these, 11 DEGs were common in both comparisons. In addition, 1567 DEGs were uniquely identified in the “*S. prenanti* treatment vs. Control” comparison and 19 DEGs were uniquely identified in the “*C. auratus* treatment vs. Control” comparison (Figure 3B).

### 3.3 Gene Ontology Analyses of DEGs

**FIGURE 4:**
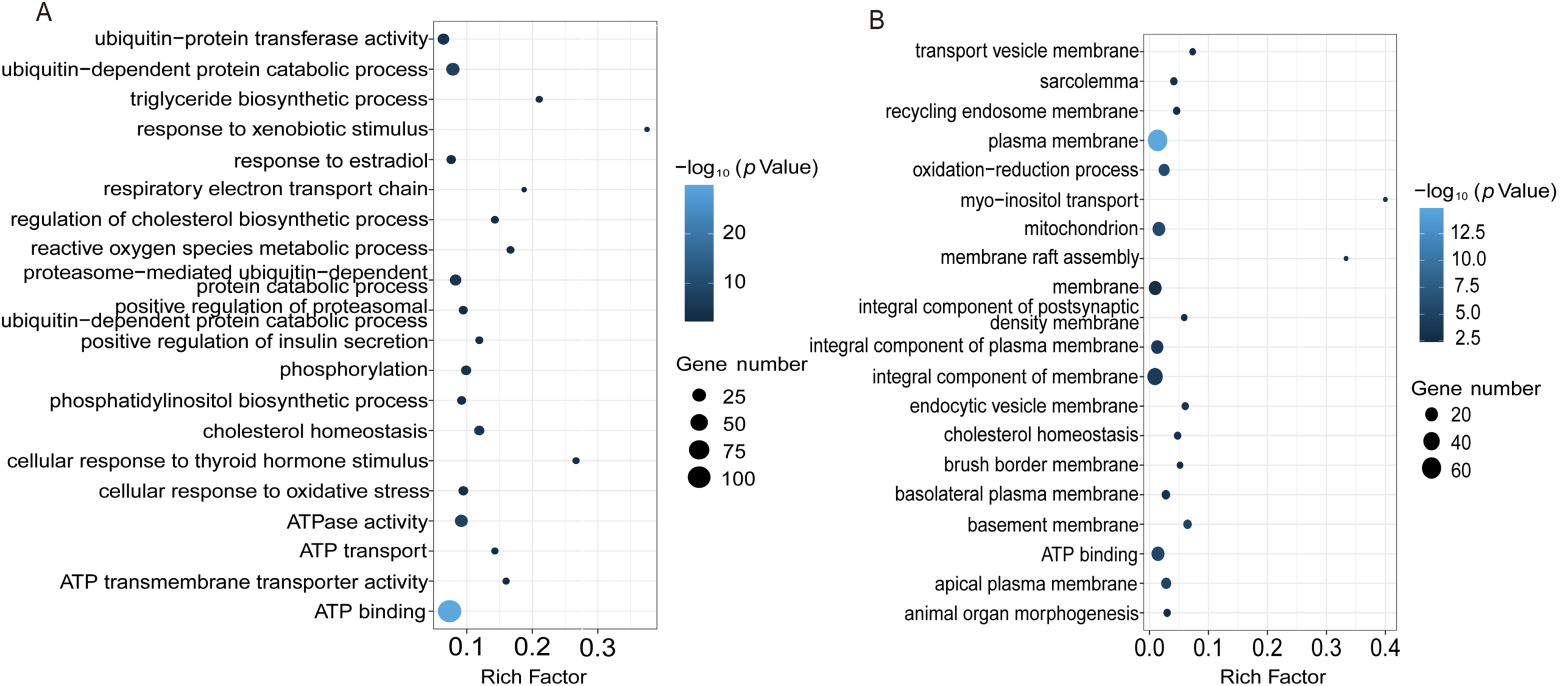
Gene ontology (GO) enrichment of DEGs from *Schizothorax prenanti* treatment (A) and *Carassius auratus* treatment (B) in liver tissue.

In liver tissue, GO terms related to stress response, endocrine, and energy were enriched in the “*S. prenanti* treatment vs. Control” comparison (Figure 4A; Table S3). For example, terms related to stress response and endocrine were enriched, including cholesterol homeostasis (10, 11.9%), regulation of cholesterol biosynthetic process (5, 14.3%), and phosphatidylinositol biosynthetic process (7, 9.2%). Terms related to energy were enriched, including ATP binding (108, 7.4%), ATP transport (4, 14.3%), respiratory electron transport chain (3, 18.8%), and reactive oxygen species metabolic process (5, 16.7%). In the “*C. auratus* treatment vs. Control” comparison, GO terms related to membrane, stress response, and energy were enriched (Figure 4B; Table S4). For example, terms related to membrane were enriched, including plasma membrane (63, 1.4%), integral component of membrane (34, 0.93%), membrane raft assembly (2, 33.33%), and apical plasma membrane (10, 2.8%). Terms related to stress response were enriched, including myo‐inositol transport (2, 40%) and cholesterol homeostasis (4, 4.8%). Terms related to energy were enriched, including ATP binding (21, 1.4%), mitochondrion (20, 1.6%), and oxidation–reduction process (13, 2.5%). Although some GO terms showed similar enrichment in both comparisons, the number of genes and enrich factors associated with stress response and energy were lower in the “*C. auratus* treatment vs. Control” comparison.

**FIGURE 5:**
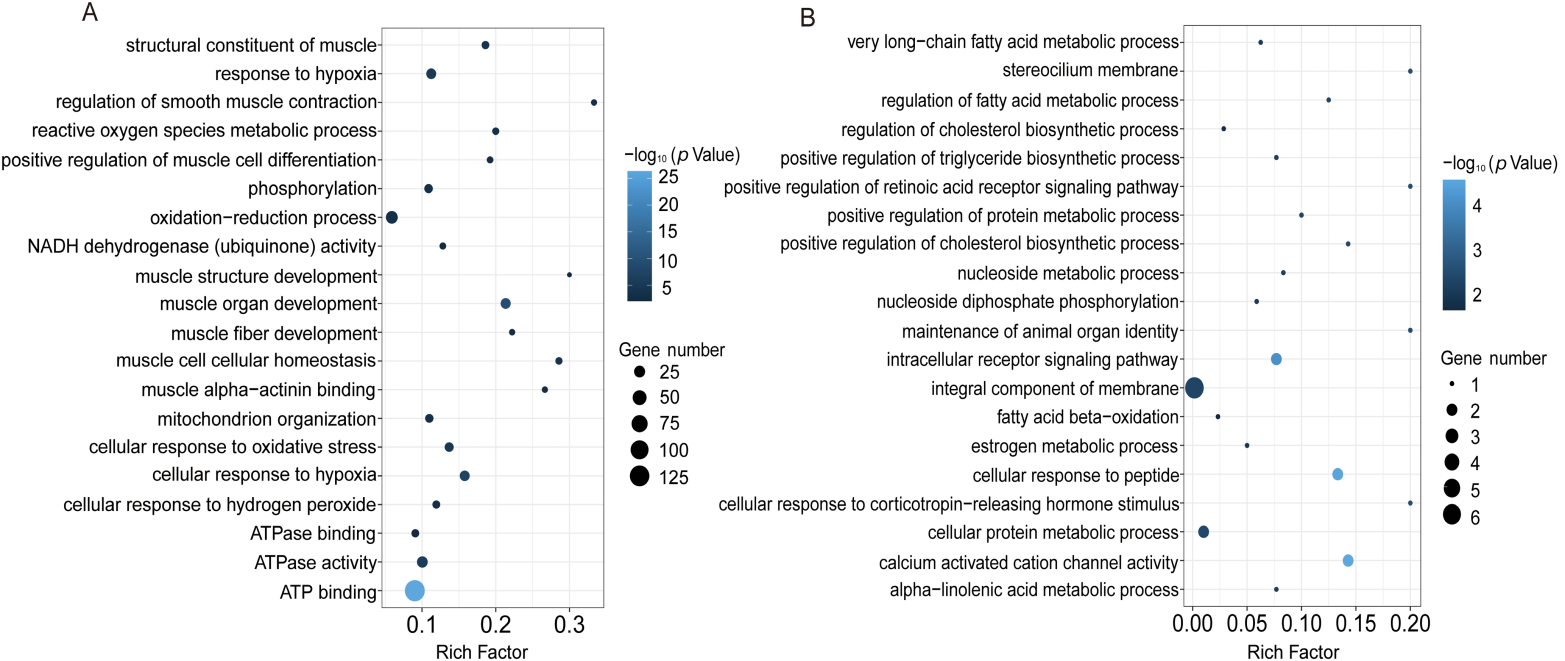
Gene ontology (GO) enrichment of DEGs from *S. prenanti* treatment (A) and *Carassius auratus* treatment (B) in tail muscle tissue.

In tail muscle tissue, GO terms related to muscle and energy were enriched in the “*S. prenanti* treatment vs. Control” comparison (Figure 5A; Table S5). For example, terms related to muscle were enriched, including muscle organ development (19, 21.3%), muscle cell cellular homeostasis (6, 28.6%), structural constituent of muscle (8, 18.6%), muscle fiber development (4, 22.2%), and muscle structure development (3, 30%). Additionally, terms related to energy were also enriched, including ATP binding (132, 9%), cellular response to hypoxia (18, 15.8%), phosphorylation (11, 10.9%), oxidation–reduction process (31, 5.9%), and reactive oxygen species metabolic process (6, 20%). In the “*C. auratus* treatment vs. Control” comparison, GO terms related to signaling pathway and membrane were enriched (Figure 5B; Table S6). For example, terms related to signaling pathways were enriched, including cellular response to peptide (2, 13.3%), intracellular receptor signaling pathway (2, 7.7%), and cellular response to corticotropin‐releasing hormone stimulus (1, 20%). Terms related to membrane were enriched, including stereocilium membrane (1, 20%) and integral component of membrane (6, 0.2%).

### 3.4 Enrichment Analyses of Kyoto Encyclopedia of Genes and Genomes Pathways of DEGs

**FIGURE 6:**
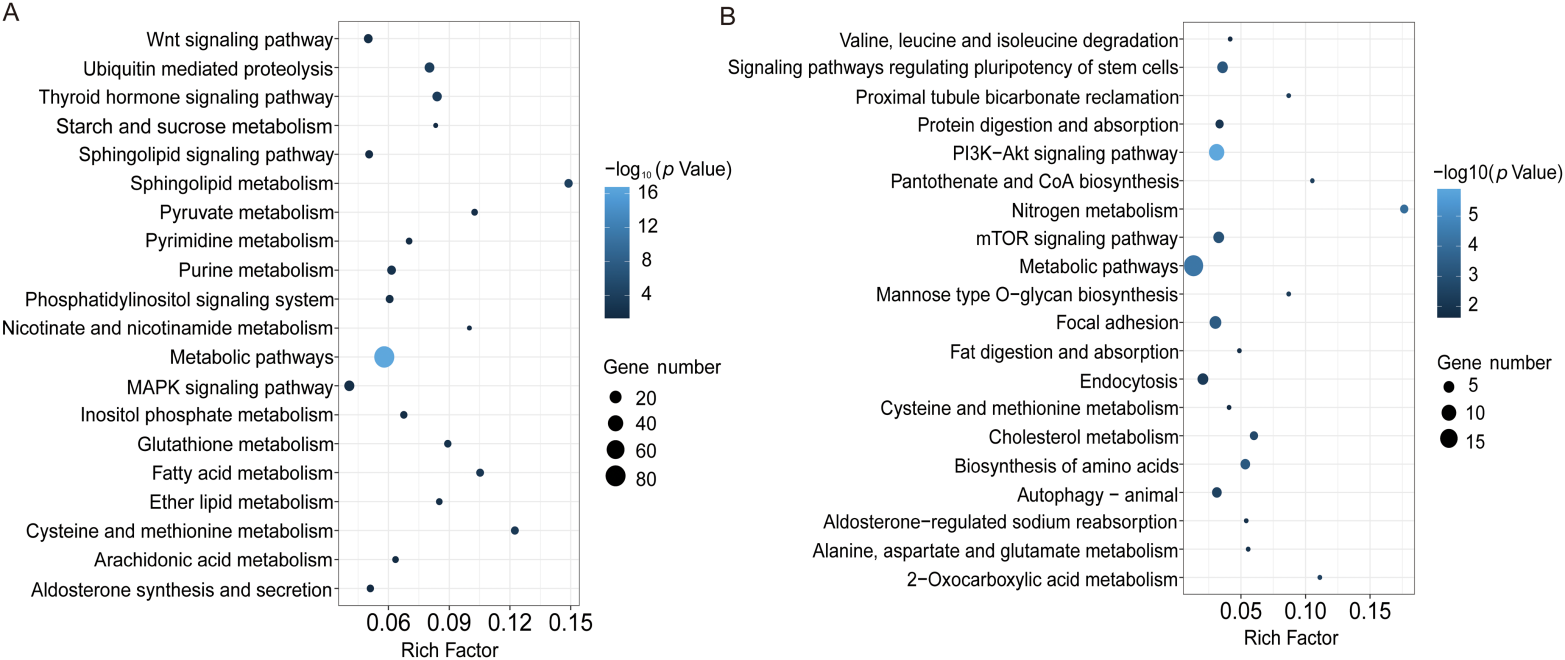
Enriched KEGG pathways of DEGs from *Schizothorax prenanti* treatment (A) and *Carassius auratus* treatment (B) in liver tissue.

In liver tissue, the significant pathways identified by Kyoto Encyclopedia of Genes and Genomes (KEGG) enrichment analysis for the “*S. prenanti* treatment vs. Control” comparison were related to stress response, endocrine, and energy metabolism (Figure 6A; Table S7). For example, KEGG pathways related to stress response and endocrine were enriched, including aldosterone synthesis and secretion (5, 5.1%), phosphatidylinositol signaling system (6, 6.1%), and thyroid hormone signaling pathway (10, 8.4%); KEGG pathways related to energy metabolism were enriched, including various metabolic (e.g., fatty acid [6, 10.5%]; pyruvate [4, 10.3%]; starch and sucrose [3, 8.3%]) pathways. However, KEGG pathways related to signaling pathway and energy metabolism were enriched in the “*C. auratus* treatment vs. Control” comparison (Figure 6B; Table S8). For example, KEGG pathways related to signaling pathways were enriched, including various signaling (e.g., PI3K‐Akt [11, 3.1%]; regulating pluripotency of stem cells [5, 3.6%]; and mTOR [5, 3.3%]) pathways; KEGG pathways related to energy metabolism were enriched, including various metabolism (e.g., cholesterol [3, 6%]; alanine, aspartate, and glutamate [2, 5.6%]) pathways. Although some KEGG pathways related to energy metabolism were enriched in both comparisons, the number of genes and enrich factors were lower in the “*C. auratus* treatment vs. Control” comparison.

**FIGURE 7:**
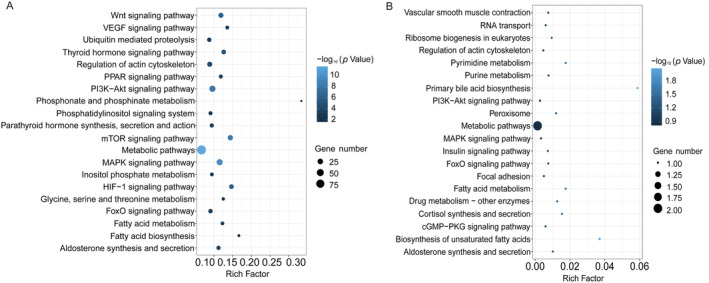
Enriched KEGG pathways of DEGs from *Schizothorax prenanti* treatment (A) and *Carassius auratus* treatment (B) in tail muscle tissue.

In tail muscle tissue, the significant pathways identified by KEGG enrichment analysis for the “*S. prenanti* treatment vs. Control” comparison were related to phenotype regulation, stress response, and energy metabolism (Figure 7A; Table S9). For example, KEGG pathways related to phenotype regulation were enriched, including regulation of actin cytoskeleton (19, 8.9%) and ubiquitin‐mediated proteolysis (12, 8.8%); KEGG pathways related to stress response were enriched, including aldosterone synthesis and secretion (11, 11.2%); KEGG pathways related to energy metabolism were enriched, including various metabolisms (e.g., fatty acid [7, 12.3%]; glycine, serine, and threonine [5, 12.5%]; phosphonate and phosphinate [2, 33.3%]). However, KEGG pathways related to signaling pathways and biosynthetic were enriched in the “*C. auratus* treatment vs. Control” comparison (Figure 7B; Table S10). For example, KEGG pathways related to signaling pathways were enriched, including various signaling (e.g., FoxO [1, 0.8%]; cGMP‐PKG [1, 0.6%]; MAPK [1, 0.3%] and PI3K‐Akt [1, 0.3%]) pathways; KEGG pathways related to biosynthetic were enriched, including various biosynthetic (e.g., primary bile acid [1, 5.9%]; unsaturated fatty acids [1, 3.7%]) pathways.

## 4 Discussion

Phenotypic plasticity is the ability of a single genotype to produce more than one alternative phenotype in response to environmental conditions (Mori et al. 2005). Phenotypic plasticity is a crucial adaptive mechanism that enables organisms to modify their traits in response to changes in their environment, facilitating the survival and reproduction of organisms in diverse habitats (Choi et al. 2023). A typical example of phenotypic plasticity is induced defenses, including prominent morphological modifications (Lima 1998). Instability of environmental conditions (e.g., periodicity of predation risk and different predators) and costs of defenses explain the inducible nature of the defensive morphs (Rozenberg et al. 2015). In this study, *R. chaochiaoensis* tadpoles developed different phenotypes induced by different fish kairomones and developed a prominent increase in TOL, TL, and TW induced by *S. prenanti* kairomone (Figure 1), suggesting that the *R. chaochiaoensis* tadpoles developed an adaptive strategy of increasing their body size. Increasing body size is the most common adaptive strategy from invertebrates to vertebrates (Carter et al. 2013; Martín‐Torrijos et al. 2016). For example, exposure to predatory fish kairomones can increase body size in *Daphnia exilis* as an adaptive response to predator‐induced stress (Carter et al. 2013). The Andean endemic amphibian *Nymphargus grandisonae* tadpoles have shown a prominent increase in tail length induced by rainbow trout *Oncorhynchus mykiss* (Martín‐Torrijos et al. 2016). Larger body size prevents prey from being swallowed by predators with limited mouth size and facilitates survival in environments with high predator abundance (Choi et al. 2023). However, this study found that *R. chaochiaoensis* tadpoles in the *C. auratus* treatment showed weaker phenotypic plasticity response, which was attributed to the short period of time that *C. auratus* was introduced into the environment in which *R. chaochiaoensis* tadpoles live, and *R. chaochiaoensis* tadpoles lacked a long co‐evolutionary history with *C. auratus*, and were unable to recognize the chemical cues that would allow them to efficiently produce phenotypic plasticity responses (Hettyey et al. 2016). Our study showed that the innate ability of tadpole prey to detect and respond to non‐native fish depends upon how long the prey and non‐native fish have been in contact with each other. Tadpoles exhibited stronger inducible phenotypic plasticity responses when exposed to native fish *S. prenanti*, which can be explained by local adaptation, as the tadpoles and the native fish share a sufficiently long co‐evolutionary history. Whereas tadpoles exposed to novel non‐native fish *C. auratus* exhibited weaker responses, this result indicated that time since arrival of the non‐native to the geographic region may be an important factor, as the *C. auratus* has been a novel translocated fish in the past 20 years or less. Nunes et al. (2014a, 2014b) suggested non‐native fish impose evolutionary changes in their prey within a few generations (for ~3–4 generations). Given that the generation time of *R. chaochiaoensis* is ~3 years (personal communication), their weaker phenotypic plasticity responses to the novel fish *C. auratus* apparently evolved within a few generations. The morphological observations of this study validate the prey naivety hypothesis.

The expression of distinct morphs requiring different levels of phenotypic changes may generally involve different numbers of genes (Matsunami et al. 2015). In this study, the number of differently expressed genes in the *S. prenanti* treatment was 3.1‐fold (liver) and 52.6‐fold (muscle) higher than that in the *C. auratus* treatment (Figures 2 and 3), which implied that *R. chaochiaoensis* tadpoles in the *S. prenanti* treatment had a stronger morphological change than the *C. auratus* treatment. In morphological observations, *R. chaochiaoensis* tadpoles in the *S. prenanti* treatment were found to have more trait variation, and the results of transcriptome analyses were consistent with morphological observations. In liver tissue and tail muscle, there were 189 and 11 common genes in both treatments, respectively, and these common genes indicated that some common GO terms and KEGG pathways were enriched under different kairomones induced, and GO and KEGG analyses showed that terms and pathways related to energy metabolism were enriched under both treatments. The liver is a major fat reserve organ, and changes in the expression of genes related to oxygen and energy in liver and muscle reflect increased metabolic rates associated with morphological changes, with animals increasing their metabolic rates and consuming more oxygen to fuel phenotypic changes (Zhu et al. 2019). However, the number of unique DEGs in liver tissue was 5 times higher, and the number of unique DEGs in tail muscle tissue was 82.5 times higher in the *S. prenanti* treatment than in the *C. auratus* treatment (Figure 3), suggesting that the *R. chaochiaoensis* tadpoles had more unique DEGs in the *S. prenanti* treatment. In GO and KEGG pathway enrichment analyses, unique DEGs in the *S. prenanti* treatment were enriched in terms and pathways related to stress response and muscle development (Figures 4, 5, 6, 7), whereas unique DEGs in the *C. auratus* treatment were enriched in terms and pathways related to signaling pathways and membranes (Figures 4, 5, 6, 7). Native anuran tadpoles may not be able to recognize novel introduced fish due to a lack of common evolutionary history (Hettyey et al. 2016), *S. prenanti*, a local native fish, shares a long co‐evolutionary history with native anurans, and *R. chaochiaoensis* tadpoles are able to sense kairomones released by *S. prenanti* and adopt the defense strategy of increasing body size, whereas *C. auratus* is a translocated fish introduced within the last 20 years or less, and *R. chaochiaoensis* tadpoles lack the co‐evolutionary history with *C. auratus* and are unable to sense kairomones released by *C. auratus*. Therefore, in the transcriptome analysis, as *R. chaochiaoensis* tadpoles sensed the kairomones released by *S. prenanti* and induced more gene expression related to stress response and locomotion escape, they could not sense the kairomones released by *C. auratus*, so only a small number of genes were changed in expression. The results of transcriptome analyses provide new molecular evidence for the prey naivety hypothesis.

As *R. chaochiaoensis* tadpoles are unable to perceive chemical cues from novel translocated fish, it may pose a serious ecological threat to native anuran larvae through direct predation or resource competition. In particular, the introduction of carnivorous or omnivorous non‐native fish, such as topmouth culter (*Culter alburnus*) and yellow‐cheek carp (*Elopichthys bambusa*), can pose significant ecological threats to the eggs and tadpoles with a long history of aquatic life (Wang et al. 2023). Xie Feng et al. surveyed Moon Lake in Shimian County during 1995 to 1999, and found considerable numbers of *Oreolalax schmidti* that were breeding (personal communication). The fishes (rainbow trout and Asian carp) were introduced into this lake in 2009 (unpublished data), and subsequent surveys in 2022–2023 showed that *Oreolalax schmidti* had absolutely disappeared from this lake. The introduction of non‐native fish in western alpine lakes and wetlands has occurred relatively recently, mostly beginning in the 1990s with China's Western Development strategy, and many fish introductions have taken place within the last decade (Wang et al. 2023). Amphibians can rapidly evolve anti‐predator abilities; however, it often takes around 30 years (Nunes et al. 2014b) or more than 3–4 generations (Hettyey et al. 2016) for them to develop responses to novel predators, and amphibians endemic to the western mountains of China typically have longer aquatic developmental periods and generation times (Fei, Ye, and Jiang 2012). Therefore, we propose the following conservation recommendations: (1) conduct a systematic assessment of the impacts of introduced non‐native fishes (especially carnivorous fishes) on threatened, narrowly distributed, and aquatic amphibians with long aquatic life stages; and (2) utilize a combination of traditional fishing methods and environmental DNA (eDNA) technology for the prevention, early warning, monitoring, and removal of introduced non‐native fish species.

A small sample size was used in this study to ensure a consistent genetic background of the tadpoles. However, it is important to note that the use of a smaller sample size may potentially compromise the statistical power and limit the strength of the conclusions drawn from this study. Therefore, future studies should consider employing a larger sample size with diverse genetic backgrounds to obtain more comprehensive and compelling findings. In addition, most studies on the effects of fish kairomones on aquatic animals have used water‐bearing fish kairomones from fish rearing tanks without quantifying the concentration of the kairomones (Petranka, Kats, and Sih 1987; Kats, Petranka, and Sih 1988). However, the concentration of kairomones may potentially influence the plasticity of morphological and life‐history traits in aquatic animals (Jin et al. 2024). In our study, we ensured that the fish in different treatment groups had identical body sizes and densities; however, due to the inherent differences between fish species, the concentration of kairomones may differ between the two treatment groups. Additionally, the kairomone concentrations in the two treatment groups may not be the optimal levels for inducing phenotypic plasticity in the tadpoles. Our study yielded promising results; by further quantifying kairomone concentrations and conducting gradient experiments to determine the optimal levels, we can gain a better understanding of the threshold for phenotypic plasticity responses in the tadpoles. Furthermore, we only confirmed the presence of both fish species in the study area without determining their density. However, the field surveys revealed that the translocated frequency of *C. auratus* was relatively high, with more than half (53.8%) of the aquatic systems in mountain ecosystems having introduced goldfish (unpublished data). Due to the differences in fish density and the duration of amphibian larvae exposure to predatory fish between insitu and ex‐situ environments, the extent of phenotypic plasticity in amphibian larvae may also differ. In the future, we will focus on the phenotypic plasticity responses of amphibian larvae in insitu environments.

## 5 Conclusion

This study aims to investigate the phenotypic plasticity and genetic responses of *R. chaochiaoensis* tadpoles to the kairomones of both native and non‐native fish species. The results indicate that under the induction of native fish kairomone, *R. chaochiaoensis* tadpoles exhibit increased TOL, TL, and TW. In contrast, there is a weaker phenotypic plasticity response in the tadpoles when exposed to the kairomone of translocated fish. At the transcriptional level, more DEGs were identified in the native fish treatment compared to the translocated fish treatment, and these genes are enriched in terms and pathways related to stress response, energy metabolism, and muscle development. This study provides new evidence for the prey naivety hypothesis from both phenotypic and transcriptional perspectives. Native anurans cannot recognize the chemical cues of novel introduced non‐native fish, and therefore become easy targets. Although the small sample size used in this study presents certain limitations, our research provides a solid foundation for future amphibian conservation efforts. It should focus on the impact of non‐native fish on threatened, narrowly distributed, and long‐aquatic life stage amphibians. Additionally, a combination of conventional fishing techniques and environmental DNA technology should be employed for the prevention, early detection, monitoring, and eradication of introduced non‐native fish species.

## Author Contributions

**Yuanfei Wang:** conceptualization (equal), data curation (equal), writing – original draft (lead). **Yudong Zhu:** methodology (equal), software (equal), writing – review and editing (equal). **Liuyang He:** software (equal). **Haoqi Yu:** visualization (equal). **Xiuqin Lin:** supervision (equal). **Jianghong Ran:** project administration (equal). **Feng Xie:** resources (equal), validation (equal), writing – review and editing (equal).

## Conflicts of Interest

The authors declare no conflicts of interest.

## Supporting information


Figure S1.

Table S1.

Table S2.

Table S3.

Table S4.

Table S5.

Table S6.

Table S7.

Table S8.

Table S9.

Table S10.

## Data Availability

The raw sequence data reported in this paper have been deposited in the Genome Sequence Archive (Genomics, Proteomics & Bioinformatics 2021) in the National Genomics Data Center (Nucleic Acids Res 2022), China National Center for Bioinformation/Beijing Institute of Genomics, Chinese Academy of Sciences (GSA: CRA016757) that are publicly accessible at https://ngdc.cncb.ac.cn/gsa. Other data are available from Figshare (DOI: https://doi.org/10.6084/m9.figshare.25941466.v1).
