# Supplementary figures and images for: Phenotypic and Transcriptomic Analysis Revealed a Lack of Risk Perception by Native Tadpoles Toward Novel Non‐Native Fish
Source: Ecol Evol. 2024 Oct 21;14(10):e70481. doi: 10.1002/ece3.70481 (PMC11493475; doi:10.1002/ece3.70481)

| 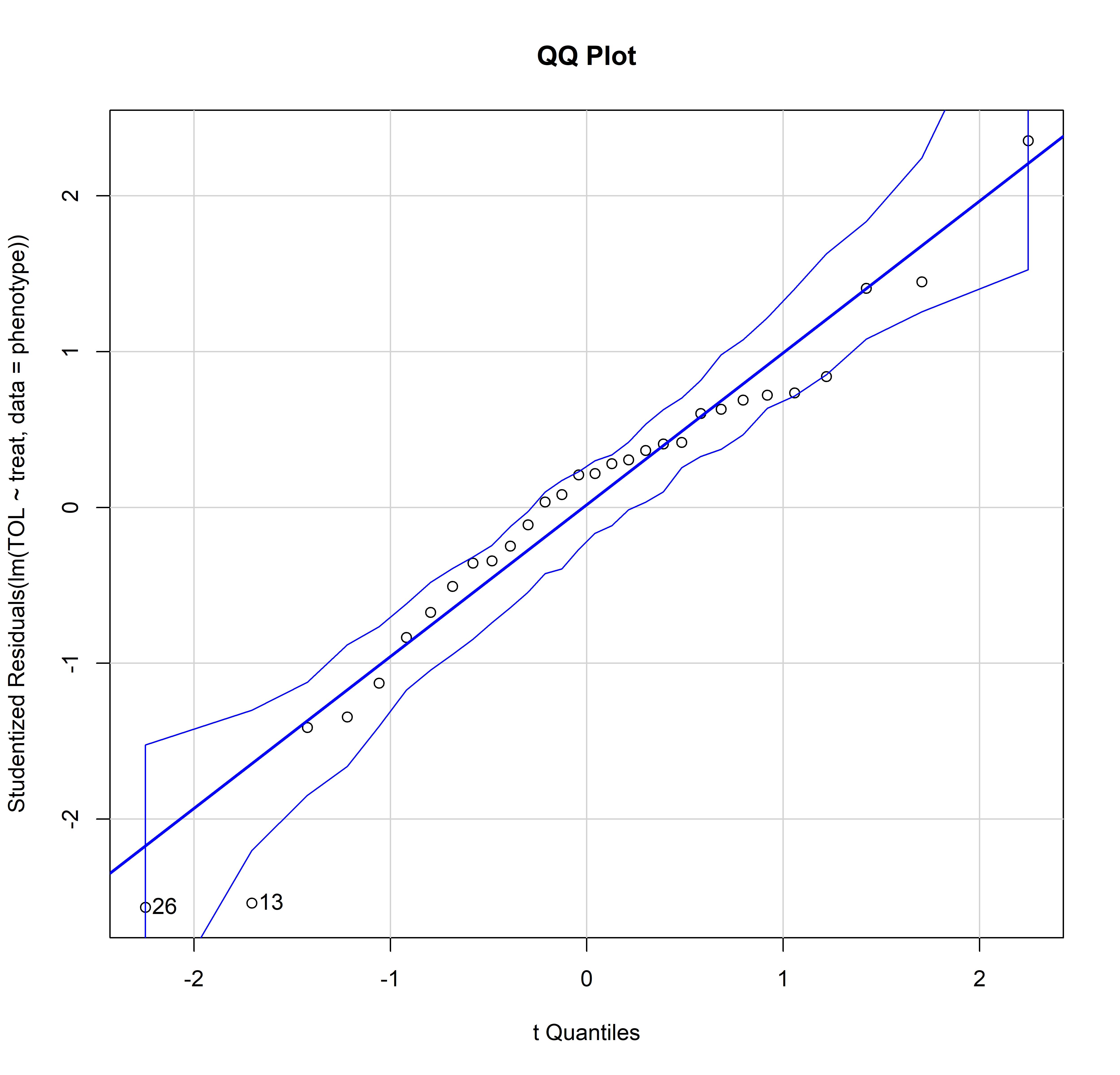 | 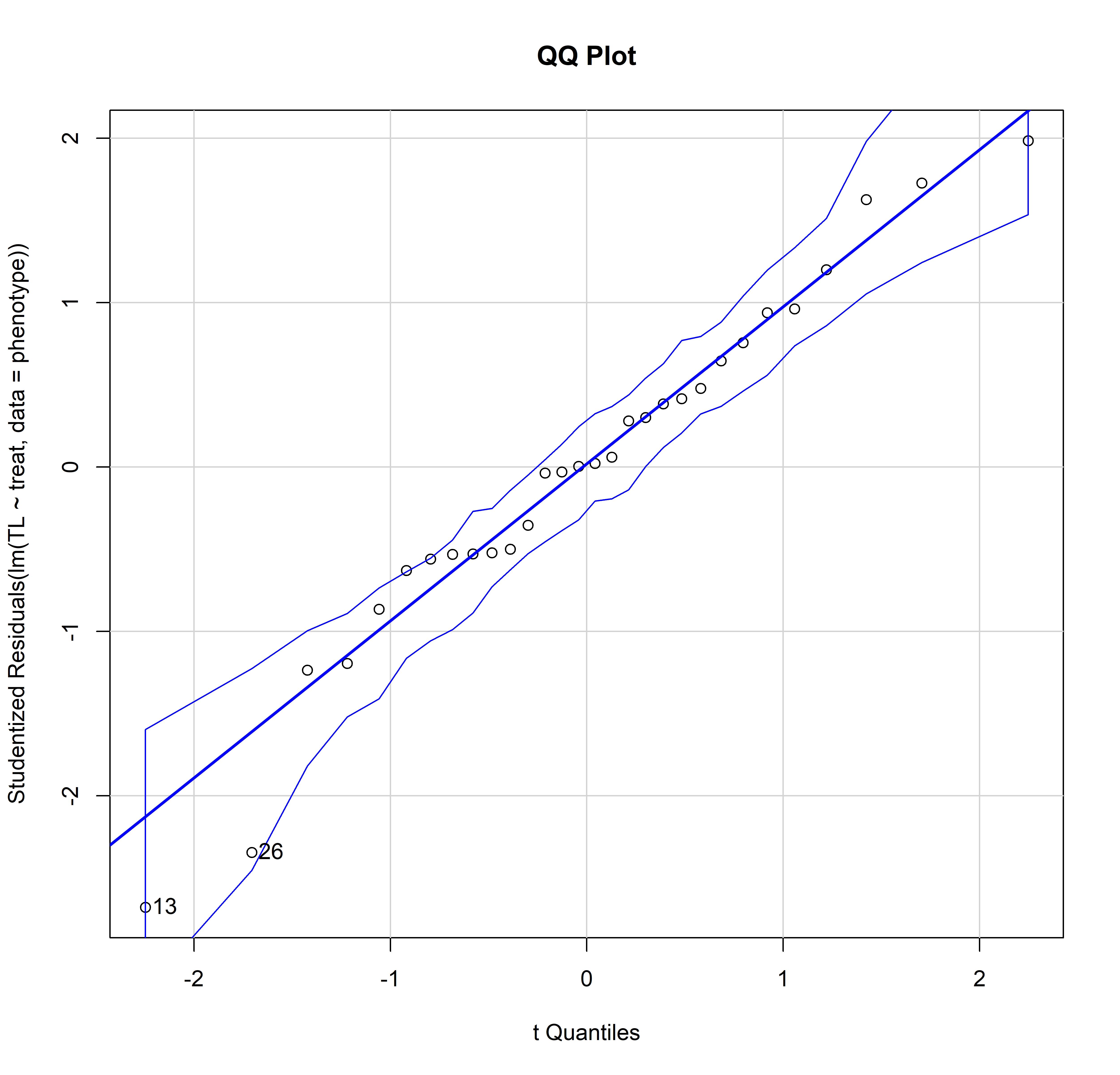 | 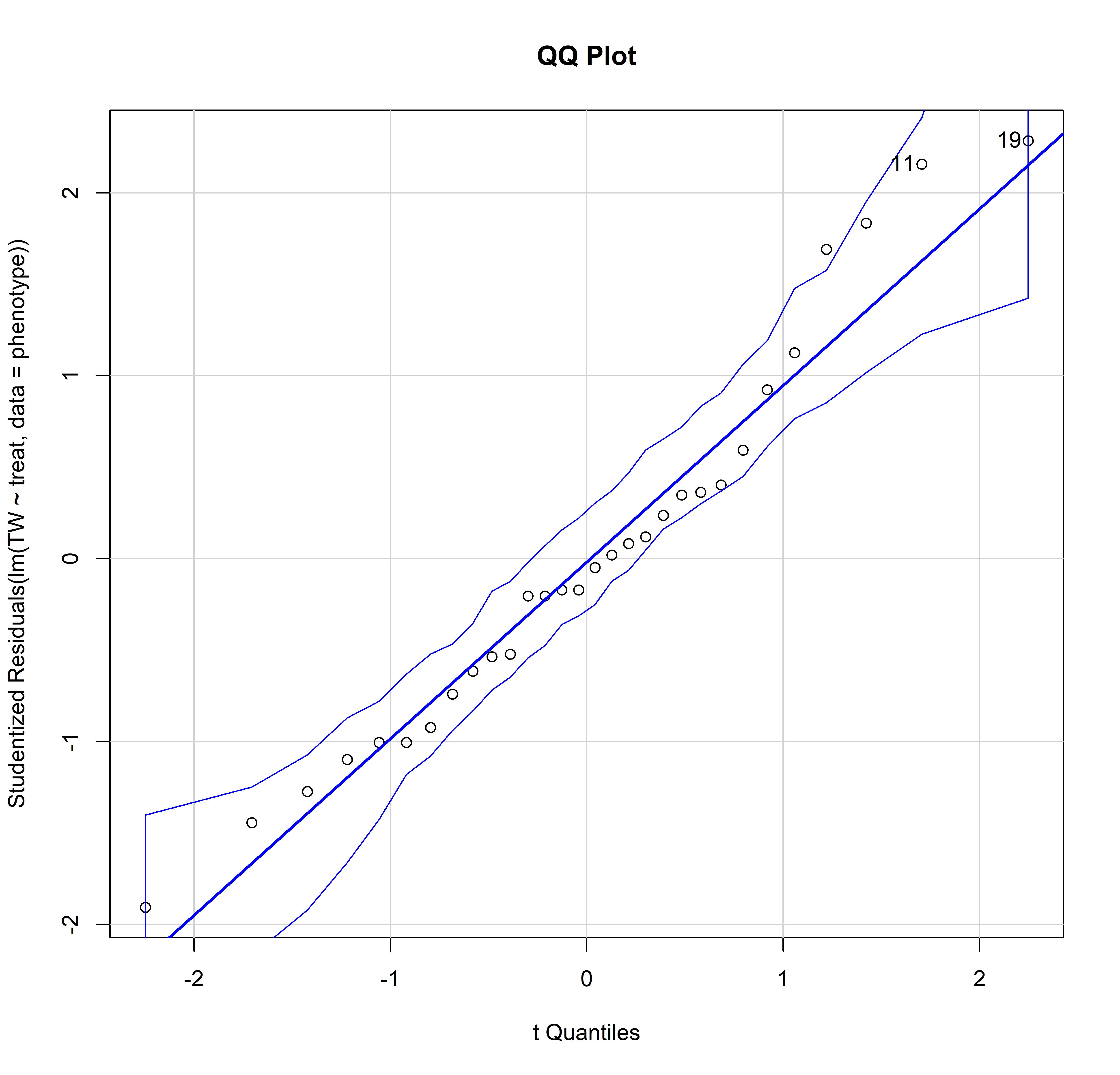 |
| --- | --- | --- |

**Figure_S1_SuppInfo.** QQ plot of the tadpole TOL, TL and TW for the test of the normal distribution.

Supplement: Supplementary file 1 — Figure S1. [file ECE3-14-e70481-s008.docx]
