# Supplementary material for: Phenotypic and Transcriptomic Analysis Revealed a Lack of Risk Perception by Native Tadpoles Toward Novel Non‐Native Fish
Source: Ecol Evol. 2024 Oct 21;14(10):e70481. doi: 10.1002/ece3.70481 (PMC11493475; doi:10.1002/ece3.70481)
Supplement: Supplementary file 2 — Table S1. [file ECE3-14-e70481-s005.docx]

**Table_S1_SuppInfo.** The descriptive statistics value of morphological trait.

| Trait | Df | *F* value | *P* value | Control  mean  (n=10) | *S. prenanti*  treatment mean (n=10) | *C.auratus* treatment mean (n=10) | Control  sd  (n=10) | *S. prenanti*  treatment sd (n=10) | *C.auratus* treatment sd (n=10) | Bartlett's  test  *P* value |
| --- | --- | --- | --- | --- | --- | --- | --- | --- | --- | --- |
| TOL | 2 | 12.59 | <0.001 | 4.587 | 5.678 | 5.188 | 0.499 | 0.344 | 0.587 | 0.3085 |
| TL | 2 | 16.69 | <0.001 | 3.049 | 3.8 | 3.255 | 0.32 | 0.158 | 0.379 | 0.05 |
| TW | 2 | 8.064 | 0.0018 | 0.401 | 0.539 | 0.387 | 0.102 | 0.113 | 0.059 | 0.1688 |

The statistical significance (*p* value) of tukey's post-hoc test:

| TOL | | | TL | | TW | |
| --- | --- | --- | --- | --- | --- | --- |
|  | Control | *S. prenanti* treatment | Control | *S. prenanti* treatment | Control | *S. prenanti* treatment |
| *S. prenanti* treatment | < 0.001 |  | < 0.001 |  | 0.007 |  |
| *C.auratus* treatment | 0.027 | 0.08 | 0.29 | 0.001 | 0.94 | 0.003 |
