# Supplementary material for: Phenotypic and Transcriptomic Analysis Revealed a Lack of Risk Perception by Native Tadpoles Toward Novel Non‐Native Fish
Source: Ecol Evol. 2024 Oct 21;14(10):e70481. doi: 10.1002/ece3.70481 (PMC11493475; doi:10.1002/ece3.70481)
Supplement: Supplementary file 3 — Table S2. [file ECE3-14-e70481-s007.docx]

**Table_S2_SuppInfo.** Average quality of sequencing sample data.

| Sample groups | Total reads count | Q30 quality percentage (%) | Base quality score | GC base percentage (%) |
| --- | --- | --- | --- | --- |
| Control - Liver | 48,482,784 | 93.56 | 37 | 48.14 |
| *C. auratus* treatment - Liver | 49,763,008 | 93.78 | 37 | 47.63 |
| *S. prenanti* treatment - Liver | 41,795,140 | 93.95 | 36 | 45.8 |
| Control - Muscle | 40,656,128 | 94.29 | 36 | 48.3 |
| *C. auratus* treatment - Muscle | 47,107,486 | 93.99 | 37 | 48.49 |
| *S. prenanti* treatment - Muscle | 48,685,442 | 93.82 | 37 | 49.48 |
