# Supplementary material for: Phenotypic and Transcriptomic Analysis Revealed a Lack of Risk Perception by Native Tadpoles Toward Novel Non‐Native Fish
Source: Ecol Evol. 2024 Oct 21;14(10):e70481. doi: 10.1002/ece3.70481 (PMC11493475; doi:10.1002/ece3.70481)
Supplement: Supplementary file 4 — Table S3. [file ECE3-14-e70481-s004.docx]

**Table_S3_SuppInfo.** Enriched GO terms of DEGs in the “*S. prenanti* treatment - Liver *vs* Control - Liver” comparison.

| Term | ID | Input number | Background number | P-Value | Corrected P-Value |
| --- | --- | --- | --- | --- | --- |
| protein binding | GO:0005515 | 564 | 11779 | 8.89E-132 | 3.41E-128 |
| cytosol | GO:0005829 | 284 | 5095 | 1.65E-61 | 3.17E-58 |
| cytoplasm | GO:0005737 | 231 | 4624 | 1.18E-40 | 1.52E-37 |
| RNA binding | GO:0003723 | 108 | 1366 | 4.80E-33 | 4.61E-30 |
| ATP binding | GO:0005524 | 108 | 1463 | 1.14E-30 | 8.79E-28 |
| nucleus | GO:0005634 | 225 | 5208 | 2.43E-30 | 1.56E-27 |
| nucleoplasm | GO:0005654 | 174 | 3630 | 1.22E-27 | 6.70E-25 |
| extracellular exosome | GO:0070062 | 125 | 2085 | 1.57E-27 | 7.52E-25 |
| mitochondrion | GO:0005739 | 93 | 1258 | 1.86E-26 | 7.95E-24 |
| membrane | GO:0016020 | 122 | 2075 | 3.75E-26 | 1.44E-23 |
| identical protein binding | GO:0042802 | 85 | 1456 | 4.07E-18 | 1.42E-15 |
| plasma membrane | GO:0005886 | 175 | 4619 | 2.67E-17 | 8.56E-15 |
| endoplasmic reticulum | GO:0005783 | 64 | 1018 | 2.94E-15 | 8.70E-13 |
| focal adhesion | GO:0005925 | 39 | 418 | 1.25E-14 | 3.44E-12 |
| lysosome | GO:0005764 | 31 | 262 | 2.18E-14 | 5.58E-12 |
| viral process | GO:0016032 | 38 | 456 | 6.95E-13 | 1.67E-10 |
| protein homodimerization activity | GO:0042803 | 45 | 660 | 3.51E-12 | 7.93E-10 |
| protein phosphorylation | GO:0006468 | 36 | 451 | 8.85E-12 | 1.89E-09 |
| integral component of membrane | GO:0016021 | 132 | 3643 | 1.15E-11 | 2.32E-09 |
| perinuclear region of cytoplasm | GO:0048471 | 45 | 697 | 1.93E-11 | 3.71E-09 |
| protein serine/threonine kinase activity | GO:0004674 | 31 | 357 | 3.57E-11 | 6.53E-09 |
| endoplasmic reticulum membrane | GO:0005789 | 52 | 942 | 1.17E-10 | 2.04E-08 |
| nucleolus | GO:0005730 | 48 | 839 | 2.02E-10 | 3.37E-08 |
| structural constituent of ribosome | GO:0003735 | 20 | 158 | 3.06E-10 | 4.90E-08 |
| nuclear speck | GO:0016607 | 31 | 400 | 4.84E-10 | 7.44E-08 |
| calcium ion binding | GO:0005509 | 42 | 693 | 5.58E-10 | 8.25E-08 |
| positive regulation of cell migration | GO:0030335 | 23 | 227 | 8.04E-10 | 1.14E-07 |
| SRP-dependent cotranslational protein targeting to membrane | GO:0006614 | 15 | 88 | 1.37E-09 | 1.88E-07 |
| Golgi apparatus | GO:0005794 | 51 | 1002 | 2.43E-09 | 3.22E-07 |
| mitochondrial matrix | GO:0005759 | 28 | 370 | 5.42E-09 | 6.94E-07 |
| nuclear-transcribed mRNA catabolic process, nonsense-mediated decay | GO:0000184 | 16 | 116 | 6.02E-09 | 7.46E-07 |
| translation | GO:0006412 | 19 | 173 | 7.15E-09 | 8.59E-07 |
| translational initiation | GO:0006413 | 16 | 126 | 1.74E-08 | 2.00E-06 |
| lysosomal membrane | GO:0005765 | 25 | 318 | 1.77E-08 | 2.00E-06 |
| negative regulation of cell population proliferation | GO:0008285 | 28 | 394 | 1.92E-08 | 2.10E-06 |
| ATPase activity | GO:0016887 | 21 | 229 | 2.17E-08 | 2.31E-06 |
| actin filament binding | GO:0051015 | 20 | 208 | 2.27E-08 | 2.35E-06 |
| protein autophosphorylation | GO:0046777 | 18 | 170 | 2.96E-08 | 3.00E-06 |
| enzyme binding | GO:0019899 | 26 | 353 | 3.15E-08 | 3.10E-06 |
| early endosome | GO:0005769 | 22 | 267 | 5.88E-08 | 5.64E-06 |
| actin cytoskeleton | GO:0015629 | 21 | 245 | 6.31E-08 | 5.80E-06 |
| ubiquitin-dependent protein catabolic process | GO:0006511 | 23 | 292 | 6.34E-08 | 5.80E-06 |
| RNA splicing | GO:0008380 | 17 | 160 | 6.82E-08 | 6.10E-06 |
| chaperone binding | GO:0051087 | 14 | 104 | 7.01E-08 | 6.12E-06 |
| protein-containing complex | GO:0032991 | 35 | 623 | 8.90E-08 | 7.60E-06 |
| viral transcription | GO:0019083 | 14 | 108 | 1.07E-07 | 8.93E-06 |
| mitochondrial inner membrane | GO:0005743 | 26 | 385 | 1.56E-07 | 1.28E-05 |
| nuclear membrane | GO:0031965 | 20 | 237 | 1.63E-07 | 1.31E-05 |
| actin binding | GO:0003779 | 21 | 266 | 2.27E-07 | 1.78E-05 |
| extracellular region | GO:0005576 | 70 | 1843 | 2.51E-07 | 1.93E-05 |
| mRNA splicing, via spliceosome | GO:0000398 | 20 | 246 | 2.84E-07 | 2.14E-05 |
| mRNA processing | GO:0006397 | 16 | 158 | 3.00E-07 | 2.22E-05 |
| intracellular membrane-bounded organelle | GO:0043231 | 39 | 788 | 3.70E-07 | 2.68E-05 |
| cadherin binding | GO:0045296 | 22 | 305 | 4.96E-07 | 3.53E-05 |
| small ribosomal subunit | GO:0015935 | 7 | 20 | 6.04E-07 | 4.22E-05 |
| magnesium ion binding | GO:0000287 | 18 | 214 | 7.01E-07 | 4.81E-05 |
| polysomal ribosome | GO:0042788 | 8 | 32 | 8.09E-07 | 5.45E-05 |
| neutrophil degranulation | GO:0043312 | 28 | 482 | 9.25E-07 | 6.13E-05 |
| metal ion binding | GO:0046872 | 80 | 2298 | 9.75E-07 | 6.35E-05 |
| cytosolic small ribosomal subunit | GO:0022627 | 9 | 47 | 1.17E-06 | 7.47E-05 |
| mRNA binding | GO:0003729 | 17 | 200 | 1.24E-06 | 7.82E-05 |
| intracellular protein transport | GO:0006886 | 20 | 273 | 1.30E-06 | 8.04E-05 |
| proteolysis | GO:0006508 | 26 | 434 | 1.32E-06 | 8.04E-05 |
| melanosome | GO:0042470 | 12 | 99 | 1.64E-06 | 9.85E-05 |
| cell-cell junction | GO:0005911 | 16 | 183 | 1.81E-06 | 0.000106821 |
| cell migration | GO:0016477 | 18 | 232 | 2.05E-06 | 0.000119499 |
| mitochondrial translational elongation | GO:0070125 | 11 | 86 | 2.77E-06 | 0.000156275 |
| mitochondrial translational termination | GO:0070126 | 11 | 86 | 2.77E-06 | 0.000156275 |
| apoptotic process | GO:0006915 | 29 | 546 | 3.19E-06 | 0.000177761 |
| protein polyubiquitination | GO:0000209 | 19 | 267 | 3.53E-06 | 0.000193556 |
| SH2 domain binding | GO:0042169 | 8 | 41 | 4.07E-06 | 0.0002204 |
| Golgi membrane | GO:0000139 | 31 | 616 | 4.19E-06 | 0.000223765 |
| regulation of apoptotic process | GO:0042981 | 16 | 197 | 4.36E-06 | 0.000229641 |
| SH3 domain binding | GO:0017124 | 13 | 131 | 4.69E-06 | 0.000243232 |
| fatty acid biosynthetic process | GO:0006633 | 8 | 42 | 4.77E-06 | 0.000244218 |
| proteasome-mediated ubiquitin-dependent protein catabolic process | GO:0043161 | 15 | 181 | 6.89E-06 | 0.000345355 |
| regulation of cellular response to heat | GO:1900034 | 10 | 77 | 6.92E-06 | 0.000345355 |
| mitochondrial intermembrane space | GO:0005758 | 10 | 78 | 7.68E-06 | 0.000378064 |
| actin filament organization | GO:0007015 | 12 | 118 | 8.61E-06 | 0.00041861 |
| protein deubiquitination | GO:0016579 | 18 | 262 | 9.92E-06 | 0.000476099 |
| sarcoplasmic reticulum membrane | GO:0033017 | 7 | 33 | 1.02E-05 | 0.000483731 |
| protein C-terminus binding | GO:0008022 | 15 | 189 | 1.11E-05 | 0.000522041 |
| intrinsic apoptotic signaling pathway in response to endoplasmic reticulum stress | GO:0070059 | 7 | 34 | 1.21E-05 | 0.000559848 |
| SMAD binding | GO:0046332 | 8 | 49 | 1.30E-05 | 0.00059618 |
| cholesterol homeostasis | GO:0042632 | 10 | 84 | 1.39E-05 | 0.000626697 |
| transcription factor binding | GO:0008134 | 20 | 325 | 1.49E-05 | 0.000665563 |
| recycling endosome | GO:0055037 | 12 | 127 | 1.70E-05 | 0.000751263 |
| microtubule binding | GO:0008017 | 17 | 252 | 2.16E-05 | 0.000942998 |
| response to drug | GO:0042493 | 18 | 280 | 2.29E-05 | 0.00098736 |
| ion channel binding | GO:0044325 | 12 | 133 | 2.60E-05 | 0.001109053 |
| TBP-class protein binding | GO:0017025 | 6 | 26 | 2.94E-05 | 0.001235162 |
| post-translational protein modification | GO:0043687 | 20 | 342 | 2.96E-05 | 0.001235162 |
| activation of JUN kinase activity | GO:0007257 | 7 | 40 | 3.06E-05 | 0.001264723 |
| spliceosomal complex | GO:0005681 | 10 | 93 | 3.10E-05 | 0.001266572 |
| protein stabilization | GO:0050821 | 14 | 183 | 3.17E-05 | 0.001276104 |
| apical part of cell | GO:0045177 | 9 | 74 | 3.19E-05 | 0.001276104 |
| lysosomal lumen | GO:0043202 | 10 | 94 | 3.37E-05 | 0.001326064 |
| extracellular matrix structural constituent | GO:0005201 | 12 | 137 | 3.40E-05 | 0.001326064 |
| unfolded protein binding | GO:0051082 | 11 | 115 | 3.42E-05 | 0.001326064 |
| liver development | GO:0001889 | 9 | 76 | 3.86E-05 | 0.001476125 |
| central nervous system development | GO:0007417 | 12 | 139 | 3.88E-05 | 0.001476125 |
| positive regulation of cell population proliferation | GO:0008284 | 25 | 501 | 3.97E-05 | 0.001496691 |
| proteolysis involved in cellular protein catabolic process | GO:0051603 | 6 | 28 | 4.23E-05 | 0.001550934 |
| podosome | GO:0002102 | 6 | 28 | 4.23E-05 | 0.001550934 |
| protein localization | GO:0008104 | 9 | 77 | 4.24E-05 | 0.001550934 |
| positive regulation of transcription by RNA polymerase II | GO:0045944 | 44 | 1159 | 4.53E-05 | 0.001632139 |
| cell junction | GO:0030054 | 14 | 190 | 4.64E-05 | 0.001632139 |
| glutamatergic synapse | GO:0098978 | 20 | 354 | 4.67E-05 | 0.001632139 |
| ruffle | GO:0001726 | 10 | 98 | 4.67E-05 | 0.001632139 |
| integrin binding | GO:0005178 | 12 | 142 | 4.71E-05 | 0.001632139 |
| basolateral plasma membrane | GO:0016323 | 15 | 216 | 4.76E-05 | 0.001632139 |
| protein kinase activity | GO:0004672 | 15 | 216 | 4.76E-05 | 0.001632139 |
| Rab GTPase binding | GO:0017137 | 12 | 143 | 5.01E-05 | 0.001704399 |
| DNA damage response, signal transduction by p53 class mediator | GO:0030330 | 5 | 17 | 5.23E-05 | 0.001760959 |
| phosphorylation | GO:0016310 | 10 | 101 | 5.90E-05 | 0.001962978 |
| adult behavior | GO:0030534 | 6 | 30 | 5.93E-05 | 0.001962978 |
| platelet degranulation | GO:0002576 | 11 | 124 | 6.44E-05 | 0.002103811 |
| microtubule | GO:0005874 | 18 | 305 | 6.52E-05 | 0.002103811 |
| protein ubiquitination | GO:0016567 | 24 | 486 | 6.57E-05 | 0.002103811 |
| amino acid binding | GO:0016597 | 5 | 18 | 6.57E-05 | 0.002103811 |
| response to toxic substance | GO:0009636 | 9 | 82 | 6.64E-05 | 0.0021066 |
| positive regulation of apoptotic process | GO:0043065 | 19 | 335 | 6.82E-05 | 0.002147141 |
| nuclear body | GO:0016604 | 18 | 307 | 7.06E-05 | 0.002203672 |
| vesicle | GO:0031982 | 13 | 173 | 7.12E-05 | 0.002206295 |
| autophagy | GO:0006914 | 11 | 126 | 7.36E-05 | 0.002262721 |
| cell projection | GO:0042995 | 11 | 127 | 7.86E-05 | 0.00239742 |
| 14-3-3 protein binding | GO:0071889 | 6 | 32 | 8.13E-05 | 0.002451983 |
| cellular amino acid metabolic process | GO:0006520 | 5 | 19 | 8.17E-05 | 0.002451983 |
| erythrocyte differentiation | GO:0030218 | 7 | 48 | 8.65E-05 | 0.002575434 |
| actin filament polymerization | GO:0030041 | 6 | 33 | 9.46E-05 | 0.002793714 |
| cell cycle arrest | GO:0007050 | 11 | 130 | 9.55E-05 | 0.002798746 |
| cytoskeleton | GO:0005856 | 21 | 406 | 0.000100215 | 0.002916118 |
| ubiquitin-protein transferase activity | GO:0004842 | 15 | 233 | 0.000105776 | 0.003054775 |
| regulation of cell growth | GO:0001558 | 8 | 68 | 0.00010799 | 0.003072509 |
| response to nutrient | GO:0007584 | 8 | 68 | 0.00010799 | 0.003072509 |
| glutathione metabolic process | GO:0006749 | 6 | 34 | 0.00010944 | 0.003090878 |
| kinase activity | GO:0016301 | 9 | 89 | 0.000118287 | 0.003316358 |
| regulation of macroautophagy | GO:0016241 | 8 | 70 | 0.00012984 | 0.003587888 |
| negative regulation of ERK1 and ERK2 cascade | GO:0070373 | 8 | 70 | 0.00012984 | 0.003587888 |
| lamellipodium | GO:0030027 | 13 | 185 | 0.000133738 | 0.003669197 |
| apical plasma membrane | GO:0016324 | 19 | 354 | 0.000134813 | 0.003672454 |
| ribosome binding | GO:0043022 | 7 | 52 | 0.000136068 | 0.003680543 |
| negative regulation of angiogenesis | GO:0016525 | 9 | 91 | 0.000138206 | 0.003712244 |
| endoplasmic reticulum lumen | GO:0005788 | 17 | 296 | 0.000140283 | 0.003741858 |
| peptidyl-serine phosphorylation | GO:0018105 | 12 | 161 | 0.000143621 | 0.00380447 |
| protein heterodimerization activity | GO:0046982 | 17 | 297 | 0.000145716 | 0.003833531 |
| cortical actin cytoskeleton | GO:0030864 | 7 | 53 | 0.000151505 | 0.003958703 |
| extracellular space | GO:0005615 | 53 | 1572 | 0.000152954 | 0.003969569 |
| postsynapse | GO:0098794 | 9 | 93 | 0.000160851 | 0.004146507 |
| regulation of small GTPase mediated signal transduction | GO:0051056 | 11 | 139 | 0.000165436 | 0.004236275 |
| positive regulation of gene expression | GO:0010628 | 20 | 391 | 0.000167163 | 0.004252139 |
| brush border | GO:0005903 | 7 | 54 | 0.000168329 | 0.004253628 |
| small GTPase mediated signal transduction | GO:0007264 | 9 | 94 | 0.000173284 | 0.004350225 |
| calcium ion import | GO:0070509 | 5 | 23 | 0.000177288 | 0.00440167 |
| cell cycle | GO:0007049 | 15 | 245 | 0.000177625 | 0.00440167 |
| cellular response to oxidative stress | GO:0034599 | 9 | 95 | 0.000186507 | 0.004555185 |
| mitochondrial large ribosomal subunit | GO:0005762 | 7 | 55 | 0.000186632 | 0.004555185 |
| protein folding | GO:0006457 | 12 | 166 | 0.000187465 | 0.004555185 |
| ADP binding | GO:0043531 | 6 | 38 | 0.000188564 | 0.004555185 |
| transforming growth factor beta receptor signaling pathway | GO:0007179 | 9 | 96 | 0.000200559 | 0.00479091 |
| regulation of alternative mRNA splicing, via spliceosome | GO:0000381 | 8 | 75 | 0.000200817 | 0.00479091 |
| protein kinase binding | GO:0019901 | 22 | 461 | 0.000205814 | 0.004822891 |
| positive regulation of extrinsic apoptotic signaling pathway via death domain receptors | GO:1902043 | 4 | 12 | 0.000205924 | 0.004822891 |
| immunological synapse formation | GO:0001771 | 4 | 12 | 0.000205924 | 0.004822891 |
| regulation of cell migration | GO:0030334 | 9 | 97 | 0.000215479 | 0.005016091 |
| intracellular signal transduction | GO:0035556 | 19 | 369 | 0.000222577 | 0.005150105 |
| collagen-containing extracellular matrix | GO:0062023 | 19 | 370 | 0.000229895 | 0.005287579 |
| extracellular matrix organization | GO:0030198 | 15 | 252 | 0.000236594 | 0.005409272 |
| glycosphingolipid metabolic process | GO:0006687 | 6 | 40 | 0.000242193 | 0.005504527 |
| cellular response to interleukin-4 | GO:0071353 | 5 | 25 | 0.000249027 | 0.005626554 |
| clathrin-coated pit | GO:0005905 | 7 | 58 | 0.000251385 | 0.005646608 |
| ficolin-1-rich granule lumen | GO:1904813 | 10 | 123 | 0.000265063 | 0.005887886 |
| alpha-linolenic acid metabolic process | GO:0036109 | 4 | 13 | 0.000265192 | 0.005887886 |
| tubulin binding | GO:0015631 | 7 | 59 | 0.000276592 | 0.006050582 |
| cell redox homeostasis | GO:0045454 | 7 | 59 | 0.000276592 | 0.006050582 |
| cytoplasmic vesicle | GO:0031410 | 16 | 285 | 0.000277246 | 0.006050582 |
| zinc ion binding | GO:0008270 | 32 | 820 | 0.000304709 | 0.00659065 |
| cytoplasmic translation | GO:0002181 | 6 | 42 | 0.00030714 | 0.00659065 |
| mRNA transport | GO:0051028 | 6 | 42 | 0.00030714 | 0.00659065 |
| cellular response to nutrient levels | GO:0031669 | 4 | 14 | 0.000335783 | 0.007165236 |
| ribosome biogenesis | GO:0042254 | 5 | 27 | 0.000340907 | 0.007234376 |
| signal transduction | GO:0007165 | 37 | 1013 | 0.00036521 | 0.007690632 |
| Sec61 translocon complex | GO:0005784 | 3 | 5 | 0.000369809 | 0.007690632 |
| positive regulation of cellular metabolic process | GO:0031325 | 3 | 5 | 0.000369809 | 0.007690632 |
| endosome | GO:0005768 | 16 | 293 | 0.000370416 | 0.007690632 |
| regulation of protein stability | GO:0031647 | 8 | 83 | 0.000378337 | 0.007812861 |
| ubiquitin protein ligase binding | GO:0031625 | 16 | 294 | 0.00038377 | 0.007865888 |
| sarcoplasmic reticulum | GO:0016529 | 6 | 44 | 0.000385 | 0.007865888 |
| cysteine-type peptidase activity | GO:0008234 | 5 | 28 | 0.000395461 | 0.008036847 |
| membrane raft | GO:0045121 | 14 | 237 | 0.000405327 | 0.008194009 |
| nuclear envelope | GO:0005635 | 12 | 182 | 0.000412144 | 0.008288188 |
| cellular response to thyroid hormone stimulus | GO:0097067 | 4 | 15 | 0.000418869 | 0.008379568 |
| ubiquitin ligase complex | GO:0000151 | 9 | 107 | 0.000422243 | 0.008403298 |
| response to organic cyclic compound | GO:0014070 | 6 | 45 | 0.000429302 | 0.008499733 |
| fatty acid metabolic process | GO:0006631 | 7 | 64 | 0.000434817 | 0.008548164 |
| GTPase activator activity | GO:0005096 | 15 | 268 | 0.000437877 | 0.008548164 |
| peptidase activity | GO:0008233 | 8 | 85 | 0.000438424 | 0.008548164 |
| signaling receptor complex adaptor activity | GO:0030159 | 5 | 29 | 0.000456351 | 0.008852757 |
| peptidyl-threonine phosphorylation | GO:0018107 | 7 | 65 | 0.000473747 | 0.009102676 |
| negative regulation of protein kinase B signaling | GO:0051898 | 6 | 46 | 0.000477479 | 0.009102676 |
| nuclear matrix | GO:0016363 | 9 | 109 | 0.000478714 | 0.009102676 |
| integral component of endoplasmic reticulum membrane | GO:0030176 | 9 | 109 | 0.000478714 | 0.009102676 |
| actin filament | GO:0005884 | 8 | 87 | 0.000506034 | 0.009522073 |
| extracellular matrix disassembly | GO:0022617 | 7 | 66 | 0.000515405 | 0.009522073 |
| platelet alpha granule lumen | GO:0031093 | 7 | 66 | 0.000515405 | 0.009522073 |
| fatty acid homeostasis | GO:0055089 | 4 | 16 | 0.000515645 | 0.009522073 |
| cellular response to hepatocyte growth factor stimulus | GO:0035729 | 4 | 16 | 0.000515645 | 0.009522073 |
| organelle membrane | GO:0031090 | 4 | 16 | 0.000515645 | 0.009522073 |
| multicellular organism development | GO:0007275 | 21 | 462 | 0.000522057 | 0.009539527 |
| reactive oxygen species metabolic process | GO:0072593 | 5 | 30 | 0.000524041 | 0.009539527 |
| platelet-derived growth factor receptor signaling pathway | GO:0048008 | 5 | 30 | 0.000524041 | 0.009539527 |
| cell surface | GO:0009986 | 25 | 598 | 0.000527933 | 0.009565046 |
| regulation of mRNA stability | GO:0043488 | 9 | 111 | 0.000541241 | 0.0095898 |
| protein localization to bicellular tight junction | GO:1902396 | 3 | 6 | 0.000546776 | 0.0095898 |
| cellular response to nutrient | GO:0031670 | 3 | 6 | 0.000546776 | 0.0095898 |
| acetyl-CoA metabolic process | GO:0006084 | 3 | 6 | 0.000546776 | 0.0095898 |
| hemoglobin biosynthetic process | GO:0042541 | 3 | 6 | 0.000546776 | 0.0095898 |
| osteoclast fusion | GO:0072675 | 3 | 6 | 0.000546776 | 0.0095898 |
| telomerase activity | GO:0003720 | 3 | 6 | 0.000546776 | 0.0095898 |
| response to glucose | GO:0009749 | 7 | 67 | 0.000559926 | 0.009775803 |
| aging | GO:0007568 | 11 | 163 | 0.000590579 | 0.010218084 |
| heparin binding | GO:0008201 | 11 | 163 | 0.000590579 | 0.010218084 |
| positive regulation of GTPase activity | GO:0043547 | 16 | 307 | 0.000598905 | 0.010315665 |
| low-density lipoprotein particle binding | GO:0030169 | 4 | 17 | 0.000627317 | 0.010756813 |
| regulation of cell adhesion | GO:0030155 | 6 | 49 | 0.000647601 | 0.011006355 |
| phosphatase binding | GO:0019902 | 6 | 49 | 0.000647601 | 0.011006355 |
| stress fiber | GO:0001725 | 7 | 69 | 0.00065812 | 0.011135861 |
| DNA damage checkpoint | GO:0000077 | 5 | 32 | 0.000681719 | 0.011434421 |
| miRNA binding | GO:0035198 | 5 | 32 | 0.000681719 | 0.011434421 |
| calcium ion transmembrane transport | GO:0070588 | 9 | 115 | 0.000686417 | 0.011463157 |
| positive regulation of protein binding | GO:0032092 | 7 | 70 | 0.000712082 | 0.011815155 |
| positive regulation of protein serine/threonine kinase activity | GO:0071902 | 6 | 50 | 0.000713646 | 0.011815155 |
| proline-rich region binding | GO:0070064 | 4 | 18 | 0.000755107 | 0.012394724 |
| postsynaptic cytosol | GO:0099524 | 4 | 18 | 0.000755107 | 0.012394724 |
| basement membrane | GO:0005604 | 8 | 93 | 0.000760986 | 0.012438067 |
| molecular adaptor activity | GO:0060090 | 7 | 71 | 0.000769487 | 0.012478273 |
| negative regulation of RNA polymerase II regulatory region sequence-specific DNA binding | GO:1903026 | 3 | 7 | 0.000769943 | 0.012478273 |
| positive regulation of actin filament polymerization | GO:0030838 | 6 | 51 | 0.00078478 | 0.012612304 |
| cellular response to UV | GO:0034644 | 6 | 51 | 0.00078478 | 0.012612304 |
| protein-containing complex binding | GO:0044877 | 17 | 349 | 0.000840387 | 0.013449692 |
| positive regulation of neuron apoptotic process | GO:0043525 | 6 | 52 | 0.000861263 | 0.013726598 |
| positive regulation of vascular associated smooth muscle cell proliferation | GO:1904707 | 5 | 34 | 0.000872413 | 0.01373336 |
| positive regulation of telomerase activity | GO:0051973 | 5 | 34 | 0.000872413 | 0.01373336 |
| amino acid transmembrane transport | GO:0003333 | 5 | 34 | 0.000872413 | 0.01373336 |
| mRNA 3'-UTR binding | GO:0003730 | 7 | 73 | 0.000895238 | 0.014035147 |
| triglyceride biosynthetic process | GO:0019432 | 4 | 19 | 0.000900239 | 0.014035579 |
| defense response to virus | GO:0051607 | 12 | 200 | 0.000902574 | 0.014035579 |
| integral component of plasma membrane | GO:0005887 | 45 | 1380 | 0.000915713 | 0.014182474 |
| signaling receptor binding | GO:0005102 | 17 | 353 | 0.000946205 | 0.014595881 |
| positive regulation of proteasomal ubiquitin-dependent protein catabolic process | GO:0032436 | 7 | 74 | 0.000963903 | 0.014750408 |
| electron transfer activity | GO:0009055 | 7 | 74 | 0.000963903 | 0.014750408 |
| regulation of cholesterol biosynthetic process | GO:0045540 | 5 | 35 | 0.00098141 | 0.014791667 |
| brain development | GO:0007420 | 13 | 231 | 0.000981702 | 0.014791667 |
| sarcolemma | GO:0042383 | 8 | 97 | 0.000982003 | 0.014791667 |
| integrin-mediated signaling pathway | GO:0007229 | 8 | 97 | 0.000982003 | 0.014791667 |
| terminal bouton | GO:0043195 | 6 | 54 | 0.001031344 | 0.015012314 |
| cytoskeletal protein binding | GO:0008092 | 6 | 54 | 0.001031344 | 0.015012314 |
| response to endoplasmic reticulum stress | GO:0034976 | 7 | 75 | 0.001036644 | 0.015012314 |
| calcium ion transport | GO:0006816 | 7 | 75 | 0.001036644 | 0.015012314 |
| GDP metabolic process | GO:0046710 | 3 | 8 | 0.001043553 | 0.015012314 |
| central nervous system neuron axonogenesis | GO:0021955 | 3 | 8 | 0.001043553 | 0.015012314 |
| sodium channel inhibitor activity | GO:0019871 | 3 | 8 | 0.001043553 | 0.015012314 |
| TORC1 complex | GO:0031931 | 3 | 8 | 0.001043553 | 0.015012314 |
| TORC1 signaling | GO:0038202 | 3 | 8 | 0.001043553 | 0.015012314 |
| poly(G) binding | GO:0034046 | 3 | 8 | 0.001043553 | 0.015012314 |
| response to xenobiotic stimulus | GO:0009410 | 3 | 8 | 0.001043553 | 0.015012314 |
| Atg1/ULK1 kinase complex | GO:1990316 | 3 | 8 | 0.001043553 | 0.015012314 |
| barbed-end actin filament capping | GO:0051016 | 4 | 20 | 0.001063945 | 0.015135603 |
| lysosomal transport | GO:0007041 | 4 | 20 | 0.001063945 | 0.015135603 |
| telomerase holoenzyme complex | GO:0005697 | 4 | 20 | 0.001063945 | 0.015135603 |
| intracellular anatomical structure | GO:0005622 | 9 | 123 | 0.001072218 | 0.015197016 |
| one-carbon metabolic process | GO:0006730 | 5 | 36 | 0.001100199 | 0.015536263 |
| neuromuscular junction | GO:0031594 | 7 | 76 | 0.001113628 | 0.01561112 |
| phosphatidylinositol biosynthetic process | GO:0006661 | 7 | 76 | 0.001113628 | 0.01561112 |
| IRE1-mediated unfolded protein response | GO:0036498 | 6 | 55 | 0.001125491 | 0.015685193 |
| regulation of cell shape | GO:0008360 | 10 | 150 | 0.00112708 | 0.015685193 |
| Wnt signaling pathway, calcium modulating pathway | GO:0007223 | 5 | 37 | 0.001229311 | 0.017046149 |
| protein localization to plasma membrane | GO:0072659 | 10 | 152 | 0.001237944 | 0.017104115 |
| translation initiation factor binding | GO:0031369 | 4 | 21 | 0.001247453 | 0.01710844 |
| vacuolar membrane | GO:0005774 | 4 | 21 | 0.001247453 | 0.01710844 |
| mRNA export from nucleus | GO:0006406 | 8 | 101 | 0.00125162 | 0.01710844 |
| neural tube closure | GO:0001843 | 7 | 78 | 0.001281009 | 0.017448073 |
| ion transmembrane transport | GO:0034220 | 12 | 209 | 0.001289729 | 0.017504772 |
| protein domain specific binding | GO:0019904 | 13 | 239 | 0.00131373 | 0.017767738 |
| negative regulation of gene expression | GO:0010629 | 12 | 210 | 0.001340147 | 0.017979994 |
| positive regulation of substrate adhesion-dependent cell spreading | GO:1900026 | 5 | 38 | 0.001369283 | 0.017979994 |
| transcription preinitiation complex | GO:0097550 | 3 | 9 | 0.001371554 | 0.017979994 |
| positive regulation of cell size | GO:0045793 | 3 | 9 | 0.001371554 | 0.017979994 |
| nucleotide metabolic process | GO:0009117 | 3 | 9 | 0.001371554 | 0.017979994 |
| cotranslational protein targeting to membrane | GO:0006613 | 3 | 9 | 0.001371554 | 0.017979994 |
| sequestering of actin monomers | GO:0042989 | 3 | 9 | 0.001371554 | 0.017979994 |
| negative regulation of sodium ion transmembrane transporter activity | GO:2000650 | 3 | 9 | 0.001371554 | 0.017979994 |
| guanylate kinase activity | GO:0004385 | 3 | 9 | 0.001371554 | 0.017979994 |
| phosphatidylserine binding | GO:0001786 | 6 | 58 | 0.001447753 | 0.018841548 |
| telomerase RNA binding | GO:0070034 | 4 | 22 | 0.001451991 | 0.018841548 |
| actin polymerization or depolymerization | GO:0008154 | 4 | 22 | 0.001451991 | 0.018841548 |
| fibrillar center | GO:0001650 | 9 | 129 | 0.001463769 | 0.018930432 |
| ribonucleoprotein complex | GO:1990904 | 10 | 156 | 0.001486367 | 0.019141717 |
| protein N-terminus binding | GO:0047485 | 8 | 104 | 0.001490074 | 0.019141717 |
| negative regulation of transcription by RNA polymerase II | GO:0000122 | 30 | 832 | 0.001530025 | 0.019589417 |
| cellular response to growth factor stimulus | GO:0071363 | 6 | 59 | 0.001569418 | 0.019960707 |
| rough endoplasmic reticulum | GO:0005791 | 6 | 59 | 0.001569418 | 0.019960707 |
| response to estradiol | GO:0032355 | 8 | 105 | 0.001577097 | 0.019992174 |
| microtubule cytoskeleton | GO:0015630 | 10 | 158 | 0.001624947 | 0.020530992 |
| positive regulation of catalytic activity | GO:0043085 | 7 | 82 | 0.001674376 | 0.020935678 |
| positive regulation of dendritic spine development | GO:0060999 | 4 | 23 | 0.001678779 | 0.020935678 |
| phagophore assembly site | GO:0000407 | 4 | 23 | 0.001678779 | 0.020935678 |
| response to interferon-gamma | GO:0034341 | 4 | 23 | 0.001678779 | 0.020935678 |
| cellular response to glucose stimulus | GO:0071333 | 6 | 60 | 0.0016987 | 0.02111556 |
| GMP metabolic process | GO:0046037 | 3 | 10 | 0.001757608 | 0.02129644 |
| cellular response to leptin stimulus | GO:0044320 | 3 | 10 | 0.001757608 | 0.02129644 |
| cellular response to leucine | GO:0071233 | 3 | 10 | 0.001757608 | 0.02129644 |
| positive regulation of cellular component movement | GO:0051272 | 3 | 10 | 0.001757608 | 0.02129644 |
| cellular response to leucine starvation | GO:1990253 | 3 | 10 | 0.001757608 | 0.02129644 |
| positive regulation of endoplasmic reticulum stress-induced intrinsic apoptotic signaling pathway | GO:1902237 | 3 | 10 | 0.001757608 | 0.02129644 |
| negative regulation of cell size | GO:0045792 | 3 | 10 | 0.001757608 | 0.02129644 |
| amyloid fibril formation | GO:1990000 | 3 | 10 | 0.001757608 | 0.02129644 |
| cell adhesion | GO:0007155 | 20 | 478 | 0.001809017 | 0.021850417 |
| Wnt signaling pathway | GO:0016055 | 11 | 189 | 0.001826508 | 0.021992526 |
| methylated histone binding | GO:0035064 | 6 | 61 | 0.001835905 | 0.022036594 |
| response to nutrient levels | GO:0031667 | 5 | 41 | 0.001859809 | 0.02225398 |
| endosome membrane | GO:0010008 | 12 | 220 | 0.001939993 | 0.02314135 |
| proton transmembrane transport | GO:1902600 | 8 | 109 | 0.001966214 | 0.023381516 |
| histone binding | GO:0042393 | 10 | 163 | 0.002017607 | 0.023918602 |
| hydrolase activity | GO:0016787 | 7 | 85 | 0.002026483 | 0.023949914 |
| positive regulation of insulin secretion | GO:0032024 | 5 | 42 | 0.002048697 | 0.024138172 |
| COPII vesicle coating | GO:0048208 | 6 | 63 | 0.002135312 | 0.025058604 |
| insulin receptor signaling pathway | GO:0008286 | 7 | 86 | 0.002155744 | 0.025058604 |
| negative regulation of peptidyl-serine phosphorylation | GO:0033137 | 4 | 25 | 0.002203936 | 0.025058604 |
| ATP transmembrane transporter activity | GO:0005347 | 4 | 25 | 0.002203936 | 0.025058604 |
| spliceosomal complex assembly | GO:0000245 | 4 | 25 | 0.002203936 | 0.025058604 |
| sphingolipid metabolic process | GO:0006665 | 4 | 25 | 0.002203936 | 0.025058604 |
| anoikis | GO:0043276 | 3 | 11 | 0.002205105 | 0.025058604 |
| actin nucleation | GO:0045010 | 3 | 11 | 0.002205105 | 0.025058604 |
| synaptic vesicle docking | GO:0016081 | 3 | 11 | 0.002205105 | 0.025058604 |
| mesodermal cell differentiation | GO:0048333 | 3 | 11 | 0.002205105 | 0.025058604 |
| protein localization to chromosome, telomeric region | GO:0070198 | 3 | 11 | 0.002205105 | 0.025058604 |
| ribonucleoprotein granule | GO:0035770 | 3 | 11 | 0.002205105 | 0.025058604 |
| regulation of RNA splicing | GO:0043484 | 5 | 43 | 0.002251203 | 0.025431976 |
| protein targeting | GO:0006605 | 5 | 43 | 0.002251203 | 0.025431976 |
| negative regulation of neuron apoptotic process | GO:0043524 | 9 | 138 | 0.002258697 | 0.025441809 |
| mitochondrial outer membrane | GO:0005741 | 10 | 166 | 0.00228771 | 0.025693253 |
| neuron differentiation | GO:0030182 | 9 | 139 | 0.002364854 | 0.02648223 |
| transcription factor TFIID complex | GO:0005669 | 5 | 44 | 0.002467891 | 0.027238466 |
| T cell activation | GO:0042110 | 5 | 44 | 0.002467891 | 0.027238466 |
| platelet aggregation | GO:0070527 | 5 | 44 | 0.002467891 | 0.027238466 |
| cellular response to amino acid starvation | GO:0034198 | 5 | 44 | 0.002467891 | 0.027238466 |
| positive regulation of protein secretion | GO:0050714 | 5 | 44 | 0.002467891 | 0.027238466 |
| late endosome | GO:0005770 | 9 | 140 | 0.002474935 | 0.027238466 |
| regulation of actin filament polymerization | GO:0030833 | 4 | 26 | 0.002504691 | 0.0274872 |
| secretory granule lumen | GO:0034774 | 8 | 114 | 0.002554744 | 0.027956611 |
| endocytic vesicle membrane | GO:0030666 | 6 | 66 | 0.002651629 | 0.02867216 |
| tRNA binding | GO:0000049 | 6 | 66 | 0.002651629 | 0.02867216 |
| glucose homeostasis | GO:0042593 | 8 | 115 | 0.002687425 | 0.02867216 |
| regulation of megakaryocyte differentiation | GO:0045652 | 5 | 45 | 0.002699323 | 0.02867216 |
| tau protein binding | GO:0048156 | 5 | 45 | 0.002699323 | 0.02867216 |
| T-tubule | GO:0030315 | 5 | 45 | 0.002699323 | 0.02867216 |
| negative regulation of neural precursor cell proliferation | GO:2000178 | 3 | 12 | 0.002717174 | 0.02867216 |
| early phagosome | GO:0032009 | 3 | 12 | 0.002717174 | 0.02867216 |
| fatty-acyl-CoA biosynthetic process | GO:0046949 | 3 | 12 | 0.002717174 | 0.02867216 |
| sequence-specific mRNA binding | GO:1990825 | 3 | 12 | 0.002717174 | 0.02867216 |
| regulation of cell-matrix adhesion | GO:0001952 | 3 | 12 | 0.002717174 | 0.02867216 |
| hepatocyte growth factor receptor signaling pathway | GO:0048012 | 3 | 12 | 0.002717174 | 0.02867216 |
| TORC2 complex | GO:0031932 | 3 | 12 | 0.002717174 | 0.02867216 |
| azurophil granule lumen | GO:0035578 | 7 | 90 | 0.002737536 | 0.028807876 |
| nuclear outer membrane | GO:0005640 | 4 | 27 | 0.002832462 | 0.029644377 |
| mitochondrial small ribosomal subunit | GO:0005763 | 4 | 27 | 0.002832462 | 0.029644377 |
| xenobiotic metabolic process | GO:0006805 | 7 | 91 | 0.002900188 | 0.030270715 |
| negative regulation of transcription, DNA-templated | GO:0045892 | 21 | 536 | 0.002979666 | 0.031015984 |
| transmembrane transport | GO:0055085 | 14 | 296 | 0.002997862 | 0.03112105 |
| cellular_component | GO:0005575 | 18 | 433 | 0.003186699 | 0.032832811 |
| ATP transport | GO:0015867 | 4 | 28 | 0.003188398 | 0.032832811 |
| endodermal cell differentiation | GO:0035987 | 4 | 28 | 0.003188398 | 0.032832811 |
| inositol phosphate metabolic process | GO:0043647 | 5 | 47 | 0.00320868 | 0.032865441 |
| negative regulation of Wnt signaling pathway | GO:0030178 | 5 | 47 | 0.00320868 | 0.032865441 |
| chaperone complex | GO:0101031 | 3 | 13 | 0.003296693 | 0.033061618 |
| response to arsenic-containing substance | GO:0046685 | 3 | 13 | 0.003296693 | 0.033061618 |
| actin filament severing | GO:0051014 | 3 | 13 | 0.003296693 | 0.033061618 |
| regulation of insulin receptor signaling pathway | GO:0046626 | 3 | 13 | 0.003296693 | 0.033061618 |
| protein neddylation | GO:0045116 | 3 | 13 | 0.003296693 | 0.033061618 |
| negative regulation of vascular endothelial growth factor receptor signaling pathway | GO:0030948 | 3 | 13 | 0.003296693 | 0.033061618 |
| cullin-RING ubiquitin ligase complex | GO:0031461 | 3 | 13 | 0.003296693 | 0.033061618 |
| response to magnesium ion | GO:0032026 | 3 | 13 | 0.003296693 | 0.033061618 |
| replication fork processing | GO:0031297 | 4 | 29 | 0.003573631 | 0.035652773 |
| regulation of Rho protein signal transduction | GO:0035023 | 4 | 29 | 0.003573631 | 0.035652773 |
| tumor necrosis factor-mediated signaling pathway | GO:0033209 | 8 | 121 | 0.003600284 | 0.035825622 |
| centriolar satellite | GO:0034451 | 7 | 95 | 0.003625869 | 0.035986981 |
| negative regulation of DNA-binding transcription factor activity | GO:0043433 | 6 | 71 | 0.003712802 | 0.036754829 |
| cell-matrix adhesion | GO:0007160 | 7 | 96 | 0.00382712 | 0.037789119 |
| growth hormone receptor signaling pathway via JAK-STAT | GO:0060397 | 3 | 14 | 0.003946301 | 0.03829466 |
| regulation of release of sequestered calcium ion into cytosol | GO:0051279 | 3 | 14 | 0.003946301 | 0.03829466 |
| bile acid metabolic process | GO:0008206 | 3 | 14 | 0.003946301 | 0.03829466 |
| production of miRNAs involved in gene silencing by miRNA | GO:0035196 | 3 | 14 | 0.003946301 | 0.03829466 |
| activation of MAPK activity | GO:0000187 | 8 | 123 | 0.003952585 | 0.03829466 |
| cellular response to starvation | GO:0009267 | 6 | 72 | 0.003958079 | 0.03829466 |
| double-stranded RNA binding | GO:0003725 | 6 | 72 | 0.003958079 | 0.03829466 |
| filopodium | GO:0030175 | 6 | 72 | 0.003958079 | 0.03829466 |
| protein targeting to mitochondrion | GO:0006626 | 4 | 30 | 0.003989269 | 0.038394386 |
| adipose tissue development | GO:0060612 | 4 | 30 | 0.003989269 | 0.038394386 |
| chromatin binding | GO:0003682 | 18 | 443 | 0.003998374 | 0.038394386 |
| cellular response to insulin stimulus | GO:0032869 | 7 | 97 | 0.004036734 | 0.038666074 |
| cytoplasmic microtubule organization | GO:0031122 | 5 | 50 | 0.004097408 | 0.039149615 |
| peptidyl-tyrosine phosphorylation | GO:0018108 | 8 | 124 | 0.004138416 | 0.03944331 |
| positive regulation of translation | GO:0045727 | 6 | 73 | 0.004215141 | 0.039976189 |
| positive regulation of protein catabolic process | GO:0045732 | 6 | 73 | 0.004215141 | 0.039976189 |
| neuronal cell body | GO:0043025 | 16 | 376 | 0.004244468 | 0.040155174 |
| endocytosis | GO:0006897 | 10 | 182 | 0.004258388 | 0.040187884 |
| axon | GO:0030424 | 14 | 310 | 0.004423992 | 0.041060704 |
| social behavior | GO:0035176 | 5 | 51 | 0.00442915 | 0.041060704 |
| neurotransmitter secretion | GO:0007269 | 5 | 51 | 0.00442915 | 0.041060704 |
| cellular response to amino acid stimulus | GO:0071230 | 5 | 51 | 0.00442915 | 0.041060704 |
| response to zinc ion | GO:0010043 | 4 | 31 | 0.004436395 | 0.041060704 |
| positive regulation of protein targeting to mitochondrion | GO:1903955 | 4 | 31 | 0.004436395 | 0.041060704 |
| mitochondrial translation | GO:0032543 | 4 | 31 | 0.004436395 | 0.041060704 |
| maintenance of blood-brain barrier | GO:0035633 | 4 | 31 | 0.004436395 | 0.041060704 |
| circadian rhythm | GO:0007623 | 6 | 74 | 0.004484322 | 0.041404524 |
| positive regulation of phosphoprotein phosphatase activity | GO:0032516 | 3 | 15 | 0.004668404 | 0.041994515 |
| lipase activity | GO:0016298 | 3 | 15 | 0.004668404 | 0.041994515 |
| positive regulation of telomerase RNA localization to Cajal body | GO:1904874 | 3 | 15 | 0.004668404 | 0.041994515 |
| protein kinase A signaling | GO:0010737 | 3 | 15 | 0.004668404 | 0.041994515 |
| positive regulation of glial cell proliferation | GO:0060252 | 3 | 15 | 0.004668404 | 0.041994515 |
| glutathione biosynthetic process | GO:0006750 | 3 | 15 | 0.004668404 | 0.041994515 |
| negative regulation of cell-cell adhesion | GO:0022408 | 3 | 15 | 0.004668404 | 0.041994515 |
| negative regulation of macroautophagy | GO:0016242 | 3 | 15 | 0.004668404 | 0.041994515 |
| vocalization behavior | GO:0071625 | 3 | 15 | 0.004668404 | 0.041994515 |
| formation of cytoplasmic translation initiation complex | GO:0001732 | 3 | 15 | 0.004668404 | 0.041994515 |
| perikaryon | GO:0043204 | 9 | 155 | 0.004668487 | 0.041994515 |
| Z disc | GO:0030018 | 8 | 127 | 0.004736467 | 0.042506475 |
| nuclear receptor transcription coactivator activity | GO:0030374 | 5 | 52 | 0.00477957 | 0.042594727 |
| positive regulation of fat cell differentiation | GO:0045600 | 5 | 52 | 0.00477957 | 0.042594727 |
| serine-type peptidase activity | GO:0008236 | 5 | 52 | 0.00477957 | 0.042594727 |
| cellular response to DNA damage stimulus | GO:0006974 | 12 | 248 | 0.004861408 | 0.043223766 |
| ceramide biosynthetic process | GO:0046513 | 4 | 32 | 0.004916069 | 0.043408323 |
| positive regulation of G1/S transition of mitotic cell cycle | GO:1900087 | 4 | 32 | 0.004916069 | 0.043408323 |
| positive regulation of Ras protein signal transduction | GO:0046579 | 4 | 32 | 0.004916069 | 0.043408323 |
| transcription by RNA polymerase II | GO:0006366 | 10 | 187 | 0.005092041 | 0.044859017 |
| Cajal body | GO:0015030 | 5 | 53 | 0.005149221 | 0.045258946 |
| endoplasmic reticulum to Golgi vesicle-mediated transport | GO:0006888 | 10 | 188 | 0.005273141 | 0.046237452 |
| postsynaptic density | GO:0014069 | 12 | 251 | 0.005315645 | 0.046237452 |
| negative regulation of protein binding | GO:0032091 | 6 | 77 | 0.005367914 | 0.046237452 |
| sodium ion transport | GO:0006814 | 6 | 77 | 0.005367914 | 0.046237452 |
| triglyceride metabolic process | GO:0006641 | 4 | 33 | 0.005429323 | 0.046237452 |
| wound healing, spreading of cells | GO:0044319 | 3 | 16 | 0.005465192 | 0.046237452 |
| regulation of JNK cascade | GO:0046328 | 3 | 16 | 0.005465192 | 0.046237452 |
| negative regulation of single stranded viral RNA replication via double stranded DNA intermediate | GO:0045869 | 3 | 16 | 0.005465192 | 0.046237452 |
| positive regulation of DNA-templated transcription, elongation | GO:0032786 | 3 | 16 | 0.005465192 | 0.046237452 |
| respiratory electron transport chain | GO:0022904 | 3 | 16 | 0.005465192 | 0.046237452 |
| positive regulation of vascular associated smooth muscle cell migration | GO:1904754 | 3 | 16 | 0.005465192 | 0.046237452 |
| mammary gland alveolus development | GO:0060749 | 3 | 16 | 0.005465192 | 0.046237452 |
| MHC class II protein complex binding | GO:0023026 | 3 | 16 | 0.005465192 | 0.046237452 |
| miRNA metabolic process | GO:0010586 | 3 | 16 | 0.005465192 | 0.046237452 |
| acyl-CoA ligase activity | GO:0003996 | 3 | 16 | 0.005465192 | 0.046237452 |
| apolipoprotein binding | GO:0034185 | 3 | 16 | 0.005465192 | 0.046237452 |
| positive regulation of proteasomal protein catabolic process | GO:1901800 | 3 | 16 | 0.005465192 | 0.046237452 |
| pyridoxal phosphate binding | GO:0030170 | 5 | 54 | 0.005538653 | 0.046653434 |
| calcium channel activity | GO:0005262 | 5 | 54 | 0.005538653 | 0.046653434 |
| protein transport | GO:0015031 | 14 | 320 | 0.00574579 | 0.0482923 |
| chromosome, telomeric region | GO:0000781 | 5 | 55 | 0.005948408 | 0.049479058 |
| tertiary granule lumen | GO:1904724 | 5 | 55 | 0.005948408 | 0.049479058 |
| positive regulation of endothelial cell migration | GO:0010595 | 5 | 55 | 0.005948408 | 0.049479058 |
| response to calcium ion | GO:0051592 | 5 | 55 | 0.005948408 | 0.049479058 |
| stereocilium | GO:0032420 | 4 | 34 | 0.005977163 | 0.049479058 |
| GTPase binding | GO:0051020 | 4 | 34 | 0.005977163 | 0.049479058 |
| response to cocaine | GO:0042220 | 4 | 34 | 0.005977163 | 0.049479058 |
