# Supplementary material for: Phenotypic and Transcriptomic Analysis Revealed a Lack of Risk Perception by Native Tadpoles Toward Novel Non‐Native Fish
Source: Ecol Evol. 2024 Oct 21;14(10):e70481. doi: 10.1002/ece3.70481 (PMC11493475; doi:10.1002/ece3.70481)
Supplement: Supplementary file 5 — Table S4. [file ECE3-14-e70481-s011.docx]

**Table_S4_SuppInfo.** Enriched GO terms of DEGs in the “*C. auratus* treatment - Liver *vs* Control - Liver” comparison.

| Term | ID | Input number | Background number | P-Value | Corrected P-Value |
| --- | --- | --- | --- | --- | --- |
| protein binding | GO:0005515 | 123 | 11779 | 5.18E-25 | 7.81E-22 |
| extracellular exosome | GO:0070062 | 48 | 2085 | 2.58E-20 | 1.94E-17 |
| plasma membrane | GO:0005886 | 63 | 4619 | 1.29E-15 | 6.48E-13 |
| extracellular region | GO:0005576 | 35 | 1843 | 1.85E-12 | 6.98E-10 |
| extracellular space | GO:0005615 | 32 | 1572 | 3.27E-12 | 9.84E-10 |
| collagen-containing extracellular matrix | GO:0062023 | 15 | 370 | 4.43E-10 | 1.11E-07 |
| cell surface | GO:0009986 | 17 | 598 | 5.32E-09 | 1.15E-06 |
| cytoplasm | GO:0005737 | 50 | 4624 | 9.47E-09 | 1.78E-06 |
| extracellular matrix structural constituent | GO:0005201 | 9 | 137 | 3.07E-08 | 5.14E-06 |
| cytosol | GO:0005829 | 48 | 5095 | 1.27E-06 | 0.000191 |
| oxidation-reduction process | GO:0055114 | 13 | 525 | 1.60E-06 | 0.00022 |
| mitochondrion | GO:0005739 | 20 | 1258 | 2.36E-06 | 0.000296 |
| cell adhesion | GO:0007155 | 12 | 478 | 3.56E-06 | 0.000412 |
| extracellular matrix organization | GO:0030198 | 9 | 252 | 4.06E-06 | 0.000437 |
| ATP binding | GO:0005524 | 21 | 1463 | 6.16E-06 | 0.00059 |
| calcium ion binding | GO:0005509 | 14 | 693 | 6.30E-06 | 0.00059 |
| cell-cell adhesion | GO:0098609 | 7 | 142 | 6.66E-06 | 0.00059 |
| basement membrane | GO:0005604 | 6 | 93 | 7.22E-06 | 0.000604 |
| brush border | GO:0005903 | 5 | 54 | 8.33E-06 | 0.00066 |
| apical plasma membrane | GO:0016324 | 10 | 354 | 8.75E-06 | 0.00066 |
| protein localization to plasma membrane | GO:0072659 | 7 | 152 | 1.02E-05 | 0.000731 |
| perinuclear region of cytoplasm | GO:0048471 | 13 | 697 | 3.07E-05 | 0.002103 |
| one-carbon metabolic process | GO:0006730 | 4 | 36 | 3.60E-05 | 0.00236 |
| neuromuscular junction | GO:0031594 | 5 | 76 | 3.93E-05 | 0.002466 |
| hemidesmosome assembly | GO:0031581 | 3 | 12 | 4.30E-05 | 0.00249 |
| collagen-activated tyrosine kinase receptor signaling pathway | GO:0038063 | 3 | 12 | 4.30E-05 | 0.00249 |
| early endosome | GO:0005769 | 8 | 267 | 4.72E-05 | 0.002635 |
| actin filament binding | GO:0051015 | 7 | 208 | 7.02E-05 | 0.003654 |
| integrin binding | GO:0005178 | 6 | 142 | 7.03E-05 | 0.003654 |
| integral component of membrane | GO:0016021 | 34 | 3643 | 7.77E-05 | 0.003904 |
| endosome | GO:0005768 | 8 | 293 | 8.86E-05 | 0.004308 |
| integral component of plasma membrane | GO:0005887 | 18 | 1380 | 9.82E-05 | 0.004542 |
| signal transduction | GO:0007165 | 15 | 1013 | 9.95E-05 | 0.004542 |
| adherens junction | GO:0005912 | 6 | 155 | 0.000112 | 0.004956 |
| presynapse | GO:0098793 | 5 | 109 | 0.000199 | 0.008564 |
| rough endoplasmic reticulum | GO:0005791 | 4 | 59 | 0.000216 | 0.009043 |
| myosin binding | GO:0017022 | 3 | 23 | 0.000236 | 0.009628 |
| endocytosis | GO:0006897 | 6 | 182 | 0.000259 | 0.010281 |
| actin filament organization | GO:0007015 | 5 | 118 | 0.000283 | 0.010938 |
| endocytic vesicle membrane | GO:0030666 | 4 | 66 | 0.000324 | 0.012215 |
| regulation of cell growth | GO:0001558 | 4 | 68 | 0.000361 | 0.013275 |
| regulation of extracellular matrix disassembly | GO:0010715 | 2 | 5 | 0.000443 | 0.014867 |
| creatine kinase activity | GO:0004111 | 2 | 5 | 0.000443 | 0.014867 |
| myo-inositol transport | GO:0015798 | 2 | 5 | 0.000443 | 0.014867 |
| positive regulation of kinase activity | GO:0033674 | 4 | 72 | 0.000444 | 0.014867 |
| sodium ion transport | GO:0006814 | 4 | 77 | 0.000565 | 0.016748 |
| endoplasmic reticulum lumen | GO:0005788 | 7 | 296 | 0.000567 | 0.016748 |
| excretion | GO:0007588 | 3 | 32 | 0.000577 | 0.016748 |
| neuron differentiation | GO:0030182 | 5 | 139 | 0.000583 | 0.016748 |
| membrane raft assembly | GO:0001765 | 2 | 6 | 0.000589 | 0.016748 |
| morphogenesis of a polarized epithelium | GO:0001738 | 2 | 6 | 0.000589 | 0.016748 |
| positive regulation of protein localization to membrane | GO:1905477 | 2 | 6 | 0.000589 | 0.016748 |
| cardiac myofibril | GO:0097512 | 2 | 6 | 0.000589 | 0.016748 |
| lipid catabolic process | GO:0016042 | 4 | 79 | 0.00062 | 0.017133 |
| basolateral plasma membrane | GO:0016323 | 6 | 216 | 0.000625 | 0.017133 |
| positive regulation of cell population proliferation | GO:0008284 | 9 | 501 | 0.000676 | 0.01799 |
| tissue development | GO:0009888 | 3 | 34 | 0.00068 | 0.01799 |
| collagen type IV trimer | GO:0005587 | 2 | 7 | 0.000755 | 0.018652 |
| membrane attack complex | GO:0005579 | 2 | 7 | 0.000755 | 0.018652 |
| glucose:sodium symporter activity | GO:0005412 | 2 | 7 | 0.000755 | 0.018652 |
| creatine metabolic process | GO:0006600 | 2 | 7 | 0.000755 | 0.018652 |
| cholesterol homeostasis | GO:0042632 | 4 | 84 | 0.000773 | 0.018784 |
| recycling endosome membrane | GO:0055038 | 4 | 87 | 0.000876 | 0.020851 |
| cell migration | GO:0016477 | 6 | 232 | 0.000898 | 0.020851 |
| focal adhesion | GO:0005925 | 8 | 418 | 0.0009 | 0.020851 |
| muscle filament sliding | GO:0030049 | 3 | 38 | 0.000921 | 0.020851 |
| regulation of glucose import | GO:0046324 | 2 | 8 | 0.000941 | 0.020851 |
| mammary gland epithelium development | GO:0061180 | 2 | 8 | 0.000941 | 0.020851 |
| COPII-coated ER to Golgi transport vesicle | GO:0030134 | 3 | 40 | 0.001059 | 0.02203 |
| peptide hormone binding | GO:0017046 | 3 | 40 | 0.001059 | 0.02203 |
| endocytic recycling | GO:0032456 | 3 | 40 | 0.001059 | 0.02203 |
| bicarbonate transport | GO:0015701 | 3 | 41 | 0.001133 | 0.02203 |
| transport vesicle membrane | GO:0030658 | 3 | 41 | 0.001133 | 0.02203 |
| homophilic cell adhesion via plasma membrane adhesion molecules | GO:0007156 | 5 | 162 | 0.001133 | 0.02203 |
| glomerular visceral epithelial cell development | GO:0072015 | 2 | 9 | 0.001146 | 0.02203 |
| manganese ion transmembrane transport | GO:0071421 | 2 | 9 | 0.001146 | 0.02203 |
| glomerular basement membrane development | GO:0032836 | 2 | 9 | 0.001146 | 0.02203 |
| actin filament bundle | GO:0032432 | 2 | 9 | 0.001146 | 0.02203 |
| lysosomal lumen | GO:0043202 | 4 | 94 | 0.001155 | 0.02203 |
| actin cytoskeleton | GO:0015629 | 6 | 245 | 0.001179 | 0.022216 |
| fatty acid biosynthetic process | GO:0006633 | 3 | 42 | 0.001209 | 0.022499 |
| sarcolemma | GO:0042383 | 4 | 97 | 0.001291 | 0.023446 |
| integrin-mediated signaling pathway | GO:0007229 | 4 | 97 | 0.001291 | 0.023446 |
| antigen processing and presentation of exogenous peptide antigen via MHC class II | GO:0019886 | 4 | 98 | 0.001339 | 0.024026 |
| tetrahydrofolate interconversion | GO:0035999 | 2 | 10 | 0.001372 | 0.024316 |
| protein autophosphorylation | GO:0046777 | 5 | 170 | 0.001394 | 0.024428 |
| identical protein binding | GO:0042802 | 16 | 1456 | 0.001469 | 0.025445 |
| glutamatergic synapse | GO:0098978 | 7 | 354 | 0.001564 | 0.026761 |
| regulation of postsynaptic membrane neurotransmitter receptor levels | GO:0099072 | 2 | 11 | 0.001616 | 0.026761 |
| morphogenesis of embryonic epithelium | GO:0016331 | 2 | 11 | 0.001616 | 0.026761 |
| phosphatidylserine 1-acylhydrolase activity | GO:0052739 | 2 | 11 | 0.001616 | 0.026761 |
| positive regulation of fibroblast proliferation | GO:0048146 | 3 | 48 | 0.001739 | 0.028485 |
| membrane | GO:0016020 | 20 | 2075 | 0.001835 | 0.0292 |
| PERK-mediated unfolded protein response | GO:0036499 | 2 | 12 | 0.00188 | 0.0292 |
| negative regulation of reactive oxygen species biosynthetic process | GO:1903427 | 2 | 12 | 0.00188 | 0.0292 |
| neurotransmitter uptake | GO:0001504 | 2 | 12 | 0.00188 | 0.0292 |
| phospholipase A1 activity | GO:0008970 | 2 | 12 | 0.00188 | 0.0292 |
| integral component of postsynaptic density membrane | GO:0099061 | 3 | 51 | 0.00205 | 0.031528 |
| complement activation, alternative pathway | GO:0006957 | 2 | 13 | 0.002162 | 0.032911 |
| negative regulation of cell migration | GO:0030336 | 4 | 115 | 0.002354 | 0.035371 |
| positive regulation of interleukin-8 production | GO:0032757 | 3 | 54 | 0.002394 | 0.035371 |
| transmembrane receptor protein tyrosine kinase activity | GO:0004714 | 3 | 54 | 0.002394 | 0.035371 |
| S100 protein binding | GO:0044548 | 2 | 14 | 0.002463 | 0.036042 |
| positive regulation of gene expression | GO:0010628 | 7 | 391 | 0.002701 | 0.039133 |
| lipase activity | GO:0016298 | 2 | 15 | 0.002783 | 0.039571 |
| vesicle budding from membrane | GO:0006900 | 2 | 15 | 0.002783 | 0.039571 |
| brush border membrane | GO:0031526 | 3 | 58 | 0.002905 | 0.040915 |
| cell redox homeostasis | GO:0045454 | 3 | 59 | 0.003042 | 0.042004 |
| dendrite self-avoidance | GO:0070593 | 2 | 16 | 0.003122 | 0.042004 |
| carbonate dehydratase activity | GO:0004089 | 2 | 16 | 0.003122 | 0.042004 |
| apolipoprotein binding | GO:0034185 | 2 | 16 | 0.003122 | 0.042004 |
| extracellular matrix structural constituent conferring compression resistance | GO:0030021 | 2 | 16 | 0.003122 | 0.042004 |
| receptor complex | GO:0043235 | 5 | 207 | 0.003204 | 0.042728 |
| adenylate cyclase-modulating G protein-coupled receptor signaling pathway | GO:0007188 | 3 | 61 | 0.003329 | 0.043699 |
| ion transmembrane transport | GO:0034220 | 5 | 209 | 0.003335 | 0.043699 |
| endocytic vesicle | GO:0030139 | 3 | 62 | 0.003478 | 0.044803 |
| low-density lipoprotein particle binding | GO:0030169 | 2 | 17 | 0.003478 | 0.044803 |
| magnesium ion binding | GO:0000287 | 5 | 214 | 0.003678 | 0.046977 |
| actin filament-based movement | GO:0030048 | 2 | 18 | 0.003853 | 0.047989 |
| cytolysis | GO:0019835 | 2 | 18 | 0.003853 | 0.047989 |
| cadherin binding involved in cell-cell adhesion | GO:0098641 | 2 | 18 | 0.003853 | 0.047989 |
| animal organ morphogenesis | GO:0009887 | 4 | 134 | 0.004007 | 0.049492 |
