# Supplementary material for: Phenotypic and Transcriptomic Analysis Revealed a Lack of Risk Perception by Native Tadpoles Toward Novel Non‐Native Fish
Source: Ecol Evol. 2024 Oct 21;14(10):e70481. doi: 10.1002/ece3.70481 (PMC11493475; doi:10.1002/ece3.70481)
Supplement: Supplementary file 6 — Table S5. [file ECE3-14-e70481-s001.docx]

**Table_S5_SuppInfo.** Enriched GO terms of DEGs in the “*S. prenanti* treatment - Muscle *vs* Control - Muscle” comparison.

| Term | ID | Input number | Background number | P-Value | Corrected P-Value |
| --- | --- | --- | --- | --- | --- |
| protein binding | GO:0005515 | 824 | 11779 | 5.60E-168 | 2.60E-164 |
| cytosol | GO:0005829 | 381 | 5095 | 3.26E-63 | 7.58E-60 |
| cytoplasm | GO:0005737 | 347 | 4624 | 6.04E-57 | 9.35E-54 |
| plasma membrane | GO:0005886 | 340 | 4619 | 8.89E-54 | 1.03E-50 |
| nucleus | GO:0005634 | 336 | 5208 | 3.36E-41 | 3.12E-38 |
| nucleoplasm | GO:0005654 | 263 | 3630 | 3.94E-39 | 3.05E-36 |
| membrane | GO:0016020 | 171 | 2075 | 9.14E-31 | 6.07E-28 |
| axon | GO:0030424 | 61 | 310 | 5.98E-28 | 3.47E-25 |
| ATP binding | GO:0005524 | 132 | 1463 | 4.54E-27 | 2.34E-24 |
| Golgi apparatus | GO:0005794 | 96 | 1002 | 1.66E-21 | 7.73E-19 |
| extracellular exosome | GO:0070062 | 149 | 2085 | 3.97E-21 | 1.68E-18 |
| RNA binding | GO:0003723 | 110 | 1366 | 3.56E-19 | 1.38E-16 |
| identical protein binding | GO:0042802 | 114 | 1456 | 5.46E-19 | 1.95E-16 |
| metal ion binding | GO:0046872 | 151 | 2298 | 2.88E-18 | 9.57E-16 |
| neuronal cell body | GO:0043025 | 52 | 376 | 5.21E-18 | 1.62E-15 |
| dendrite | GO:0030425 | 54 | 420 | 2.09E-17 | 6.07E-15 |
| protein phosphorylation | GO:0006468 | 54 | 451 | 3.28E-16 | 8.98E-14 |
| signal transduction | GO:0007165 | 84 | 1013 | 1.55E-15 | 3.99E-13 |
| perinuclear region of cytoplasm | GO:0048471 | 67 | 697 | 2.09E-15 | 5.12E-13 |
| negative regulation of transcription by RNA polymerase II | GO:0000122 | 74 | 832 | 2.97E-15 | 6.89E-13 |
| glutamatergic synapse | GO:0098978 | 45 | 354 | 1.43E-14 | 3.17E-12 |
| protein-containing complex | GO:0032991 | 61 | 623 | 1.83E-14 | 3.86E-12 |
| synapse | GO:0045202 | 49 | 420 | 1.92E-14 | 3.88E-12 |
| positive regulation of transcription by RNA polymerase II | GO:0045944 | 88 | 1159 | 3.64E-14 | 7.06E-12 |
| integral component of plasma membrane | GO:0005887 | 98 | 1380 | 5.53E-14 | 1.03E-11 |
| focal adhesion | GO:0005925 | 48 | 418 | 6.06E-14 | 1.08E-11 |
| neuron projection | GO:0043005 | 42 | 336 | 1.80E-13 | 3.09E-11 |
| growth cone | GO:0030426 | 27 | 140 | 5.86E-13 | 9.72E-11 |
| positive regulation of transcription, DNA-templated | GO:0045893 | 54 | 556 | 7.89E-13 | 1.26E-10 |
| cytoskeleton | GO:0005856 | 44 | 406 | 3.97E-12 | 6.15E-10 |
| cell adhesion | GO:0007155 | 48 | 478 | 5.08E-12 | 7.61E-10 |
| integral component of membrane | GO:0016021 | 184 | 3643 | 1.03E-11 | 1.49E-09 |
| nervous system development | GO:0007399 | 37 | 305 | 1.08E-11 | 1.53E-09 |
| negative regulation of transcription, DNA-templated | GO:0045892 | 50 | 536 | 2.11E-11 | 2.84E-09 |
| axon guidance | GO:0007411 | 31 | 222 | 2.14E-11 | 2.84E-09 |
| nuclear chromatin | GO:0000790 | 69 | 915 | 3.03E-11 | 3.92E-09 |
| viral process | GO:0016032 | 45 | 456 | 3.97E-11 | 4.99E-09 |
| nuclear speck | GO:0016607 | 41 | 400 | 1.02E-10 | 1.25E-08 |
| mitochondrion | GO:0005739 | 83 | 1258 | 1.57E-10 | 1.87E-08 |
| protein serine/threonine kinase activity | GO:0004674 | 38 | 357 | 1.84E-10 | 2.14E-08 |
| endosome | GO:0005768 | 34 | 293 | 2.15E-10 | 2.44E-08 |
| muscle organ development | GO:0007517 | 19 | 89 | 3.48E-10 | 3.85E-08 |
| microtubule binding | GO:0008017 | 31 | 252 | 3.69E-10 | 3.99E-08 |
| Golgi membrane | GO:0000139 | 51 | 616 | 6.49E-10 | 6.85E-08 |
| cell cycle arrest | GO:0007050 | 22 | 130 | 7.18E-10 | 7.41E-08 |
| sarcolemma | GO:0042383 | 19 | 97 | 1.23E-09 | 1.24E-07 |
| intracellular signal transduction | GO:0035556 | 37 | 369 | 1.43E-09 | 1.42E-07 |
| microtubule | GO:0005874 | 33 | 305 | 1.97E-09 | 1.91E-07 |
| axon cytoplasm | GO:1904115 | 15 | 57 | 2.17E-09 | 2.06E-07 |
| Z disc | GO:0030018 | 21 | 127 | 2.51E-09 | 2.34E-07 |
| intracellular membrane-bounded organelle | GO:0043231 | 58 | 788 | 2.66E-09 | 2.42E-07 |
| extracellular region | GO:0005576 | 104 | 1843 | 2.80E-09 | 2.46E-07 |
| DNA-binding transcription factor activity, RNA polymerase II-specific | GO:0000981 | 62 | 874 | 2.81E-09 | 2.46E-07 |
| perikaryon | GO:0043204 | 23 | 155 | 2.86E-09 | 2.46E-07 |
| neuron projection development | GO:0031175 | 20 | 116 | 3.18E-09 | 2.69E-07 |
| calmodulin binding | GO:0005516 | 26 | 200 | 3.37E-09 | 2.80E-07 |
| brain development | GO:0007420 | 28 | 231 | 3.47E-09 | 2.83E-07 |
| zinc ion binding | GO:0008270 | 59 | 820 | 4.21E-09 | 3.38E-07 |
| extracellular matrix organization | GO:0030198 | 29 | 252 | 5.32E-09 | 4.18E-07 |
| cytoplasmic vesicle | GO:0031410 | 31 | 285 | 5.39E-09 | 4.18E-07 |
| negative regulation of cell population proliferation | GO:0008285 | 37 | 394 | 7.19E-09 | 5.48E-07 |
| positive regulation of cell migration | GO:0030335 | 27 | 227 | 9.46E-09 | 7.09E-07 |
| actin cytoskeleton | GO:0015629 | 28 | 245 | 1.11E-08 | 8.21E-07 |
| cell surface | GO:0009986 | 47 | 598 | 1.36E-08 | 9.88E-07 |
| axonogenesis | GO:0007409 | 16 | 78 | 1.39E-08 | 9.97E-07 |
| collagen-containing extracellular matrix | GO:0062023 | 35 | 370 | 1.54E-08 | 1.08E-06 |
| actin binding | GO:0003779 | 29 | 266 | 1.59E-08 | 1.10E-06 |
| enzyme binding | GO:0019899 | 34 | 353 | 1.64E-08 | 1.12E-06 |
| early endosome | GO:0005769 | 29 | 267 | 1.71E-08 | 1.15E-06 |
| postsynaptic density | GO:0014069 | 28 | 251 | 1.79E-08 | 1.19E-06 |
| GTPase activator activity | GO:0005096 | 29 | 268 | 1.85E-08 | 1.21E-06 |
| heart development | GO:0007507 | 24 | 189 | 2.01E-08 | 1.30E-06 |
| calcium ion binding | GO:0005509 | 51 | 693 | 2.43E-08 | 1.55E-06 |
| microtubule cytoskeleton organization | GO:0000226 | 20 | 133 | 2.49E-08 | 1.56E-06 |
| actin filament binding | GO:0051015 | 25 | 208 | 2.75E-08 | 1.70E-06 |
| ubiquitin-dependent protein catabolic process | GO:0006511 | 30 | 292 | 3.06E-08 | 1.87E-06 |
| cell-cell junction | GO:0005911 | 23 | 183 | 4.68E-08 | 2.82E-06 |
| nucleolus | GO:0005730 | 57 | 839 | 4.91E-08 | 2.92E-06 |
| endoplasmic reticulum | GO:0005783 | 65 | 1018 | 5.02E-08 | 2.95E-06 |
| cell migration | GO:0016477 | 26 | 232 | 5.26E-08 | 3.05E-06 |
| basolateral plasma membrane | GO:0016323 | 25 | 216 | 5.36E-08 | 3.07E-06 |
| adherens junction | GO:0005912 | 21 | 155 | 5.65E-08 | 3.20E-06 |
| in utero embryonic development | GO:0001701 | 24 | 202 | 6.30E-08 | 3.52E-06 |
| cellular response to hypoxia | GO:0071456 | 18 | 114 | 6.37E-08 | 3.52E-06 |
| RNA polymerase II cis-regulatory region sequence-specific DNA binding | GO:0000978 | 48 | 656 | 7.34E-08 | 4.01E-06 |
| positive regulation of GTPase activity | GO:0043547 | 30 | 307 | 8.42E-08 | 4.55E-06 |
| protein domain specific binding | GO:0019904 | 26 | 239 | 8.99E-08 | 4.80E-06 |
| receptor complex | GO:0043235 | 24 | 207 | 9.54E-08 | 5.04E-06 |
| stress fiber | GO:0001725 | 14 | 69 | 1.25E-07 | 6.53E-06 |
| negative regulation of apoptotic process | GO:0043066 | 39 | 487 | 1.47E-07 | 7.58E-06 |
| transcription regulatory region sequence-specific DNA binding | GO:0000976 | 27 | 263 | 1.50E-07 | 7.64E-06 |
| positive regulation of apoptotic process | GO:0043065 | 31 | 335 | 1.57E-07 | 7.95E-06 |
| protein autophosphorylation | GO:0046777 | 21 | 170 | 2.30E-07 | 1.15E-05 |
| late endosome | GO:0005770 | 19 | 140 | 2.33E-07 | 1.15E-05 |
| regulation of transcription by RNA polymerase II | GO:0006357 | 51 | 751 | 2.51E-07 | 1.23E-05 |
| beta-catenin binding | GO:0008013 | 15 | 86 | 2.54E-07 | 1.23E-05 |
| transcription factor binding | GO:0008134 | 30 | 325 | 2.61E-07 | 1.25E-05 |
| cell population proliferation | GO:0008283 | 19 | 142 | 2.84E-07 | 1.35E-05 |
| synapse assembly | GO:0007416 | 13 | 63 | 2.96E-07 | 1.39E-05 |
| chromatin binding | GO:0003682 | 36 | 443 | 3.16E-07 | 1.47E-05 |
| protein tyrosine kinase activity | GO:0004713 | 15 | 89 | 3.75E-07 | 1.73E-05 |
| positive regulation of gene expression | GO:0010628 | 33 | 391 | 4.45E-07 | 2.03E-05 |
| response to retinoic acid | GO:0032526 | 11 | 44 | 4.69E-07 | 2.12E-05 |
| ion channel binding | GO:0044325 | 18 | 133 | 4.99E-07 | 2.23E-05 |
| proteolysis | GO:0006508 | 35 | 434 | 5.42E-07 | 2.40E-05 |
| amyloid-beta binding | GO:0001540 | 14 | 80 | 6.11E-07 | 2.68E-05 |
| protein kinase activity | GO:0004672 | 23 | 216 | 6.87E-07 | 2.98E-05 |
| protein localization to plasma membrane | GO:0072659 | 19 | 152 | 7.26E-07 | 3.12E-05 |
| positive regulation of cell population proliferation | GO:0008284 | 38 | 501 | 7.34E-07 | 3.13E-05 |
| protein kinase binding | GO:0019901 | 36 | 461 | 7.60E-07 | 3.21E-05 |
| lamellipodium | GO:0030027 | 21 | 185 | 8.11E-07 | 3.40E-05 |
| peptidyl-tyrosine phosphorylation | GO:0018108 | 17 | 124 | 8.75E-07 | 3.63E-05 |
| tubulin binding | GO:0015631 | 12 | 59 | 9.64E-07 | 3.94E-05 |
| angiogenesis | GO:0001525 | 24 | 238 | 9.67E-07 | 3.94E-05 |
| positive regulation of cold-induced thermogenesis | GO:0120162 | 15 | 97 | 9.93E-07 | 4.01E-05 |
| learning | GO:0007612 | 12 | 60 | 1.13E-06 | 4.51E-05 |
| sequence-specific double-stranded DNA binding | GO:1990837 | 40 | 553 | 1.15E-06 | 4.58E-05 |
| integrin binding | GO:0005178 | 18 | 142 | 1.18E-06 | 4.60E-05 |
| cell projection | GO:0042995 | 17 | 127 | 1.18E-06 | 4.60E-05 |
| insulin receptor signaling pathway | GO:0008286 | 14 | 86 | 1.32E-06 | 5.10E-05 |
| lysosome | GO:0005764 | 25 | 262 | 1.46E-06 | 5.61E-05 |
| lysosomal membrane | GO:0005765 | 28 | 318 | 1.52E-06 | 5.79E-05 |
| positive regulation of canonical Wnt signaling pathway | GO:0090263 | 18 | 145 | 1.54E-06 | 5.83E-05 |
| neurotransmitter secretion | GO:0007269 | 11 | 51 | 1.65E-06 | 6.20E-05 |
| ATPase activity | GO:0016887 | 23 | 229 | 1.72E-06 | 6.39E-05 |
| neutrophil degranulation | GO:0043312 | 36 | 482 | 1.98E-06 | 7.30E-05 |
| negative regulation of macroautophagy | GO:0016242 | 7 | 15 | 2.08E-06 | 7.57E-05 |
| synaptic vesicle | GO:0008021 | 17 | 133 | 2.09E-06 | 7.57E-05 |
| chaperone binding | GO:0051087 | 15 | 104 | 2.16E-06 | 7.80E-05 |
| endocytosis | GO:0006897 | 20 | 182 | 2.32E-06 | 8.28E-05 |
| neural tube closure | GO:0001843 | 13 | 78 | 2.48E-06 | 8.80E-05 |
| cation channel activity | GO:0005261 | 10 | 43 | 2.73E-06 | 9.63E-05 |
| exocytosis | GO:0006887 | 14 | 93 | 3.00E-06 | 0.000104835 |
| negative regulation of neuron apoptotic process | GO:0043524 | 17 | 138 | 3.28E-06 | 0.000113759 |
| positive regulation of axon extension | GO:0045773 | 9 | 34 | 3.49E-06 | 0.000120138 |
| neuron differentiation | GO:0030182 | 17 | 139 | 3.58E-06 | 0.000122408 |
| neuron migration | GO:0001764 | 15 | 110 | 4.03E-06 | 0.000136626 |
| cell junction | GO:0030054 | 20 | 190 | 4.20E-06 | 0.000141486 |
| outflow tract morphogenesis | GO:0003151 | 10 | 46 | 4.61E-06 | 0.000154199 |
| sodium ion transmembrane transport | GO:0035725 | 14 | 97 | 4.65E-06 | 0.000154508 |
| protein transport | GO:0015031 | 27 | 320 | 4.83E-06 | 0.000158681 |
| extracellular space | GO:0005615 | 81 | 1572 | 4.85E-06 | 0.000158681 |
| multicellular organism development | GO:0007275 | 34 | 462 | 5.07E-06 | 0.000164844 |
| response to hypoxia | GO:0001666 | 18 | 160 | 5.43E-06 | 0.000174592 |
| cardiac conduction | GO:0061337 | 10 | 47 | 5.45E-06 | 0.000174592 |
| locomotory behavior | GO:0007626 | 13 | 85 | 5.76E-06 | 0.000183016 |
| retrograde axonal transport | GO:0008090 | 6 | 11 | 5.79E-06 | 0.000183016 |
| positive regulation of cell adhesion | GO:0045785 | 10 | 48 | 6.41E-06 | 0.000201285 |
| positive regulation of phosphatidylinositol 3-kinase signaling | GO:0014068 | 13 | 86 | 6.45E-06 | 0.000201285 |
| calcium ion transmembrane transport | GO:0070588 | 15 | 115 | 6.56E-06 | 0.000203227 |
| positive regulation of endothelial cell proliferation | GO:0001938 | 12 | 73 | 6.81E-06 | 0.00020956 |
| extracellular matrix | GO:0031012 | 22 | 233 | 7.11E-06 | 0.000217517 |
| cytoskeleton-dependent intracellular transport | GO:0030705 | 7 | 19 | 7.25E-06 | 0.000220262 |
| proteasome-mediated ubiquitin-dependent protein catabolic process | GO:0043161 | 19 | 181 | 7.51E-06 | 0.000225397 |
| kinesin complex | GO:0005871 | 10 | 49 | 7.52E-06 | 0.000225397 |
| lysosome localization | GO:0032418 | 8 | 28 | 7.62E-06 | 0.000226922 |
| protein-containing complex binding | GO:0044877 | 28 | 349 | 7.82E-06 | 0.000231421 |
| intracellular protein transport | GO:0006886 | 24 | 273 | 8.50E-06 | 0.000249949 |
| cis-Golgi network | GO:0005801 | 11 | 62 | 8.64E-06 | 0.000252441 |
| cellular response to amino acid stimulus | GO:0071230 | 10 | 51 | 1.02E-05 | 0.000297275 |
| protein localization | GO:0008104 | 12 | 77 | 1.10E-05 | 0.000318625 |
| osteoblast differentiation | GO:0001649 | 14 | 106 | 1.16E-05 | 0.000333254 |
| glutamate secretion | GO:0014047 | 8 | 30 | 1.17E-05 | 0.000333254 |
| ubiquitin ligase complex | GO:0000151 | 14 | 107 | 1.28E-05 | 0.000361345 |
| response to insulin | GO:0032868 | 11 | 65 | 1.28E-05 | 0.000361345 |
| cation transmembrane transport | GO:0098655 | 9 | 41 | 1.29E-05 | 0.000361345 |
| GTPase activity | GO:0003924 | 26 | 320 | 1.33E-05 | 0.000369419 |
| sensory perception of pain | GO:0019233 | 10 | 53 | 1.38E-05 | 0.00038062 |
| voluntary musculoskeletal movement | GO:0050882 | 5 | 7 | 1.41E-05 | 0.000388779 |
| ERBB2 signaling pathway | GO:0038128 | 8 | 31 | 1.43E-05 | 0.000389424 |
| anterograde axonal transport | GO:0008089 | 8 | 31 | 1.43E-05 | 0.000389424 |
| terminal bouton | GO:0043195 | 10 | 54 | 1.59E-05 | 0.000424207 |
| transmembrane receptor protein tyrosine kinase activity | GO:0004714 | 10 | 54 | 1.59E-05 | 0.000424207 |
| brush border | GO:0005903 | 10 | 54 | 1.59E-05 | 0.000424207 |
| presynaptic membrane | GO:0042734 | 11 | 67 | 1.65E-05 | 0.000438969 |
| cellular response to oxidative stress | GO:0034599 | 13 | 95 | 1.69E-05 | 0.000445388 |
| ubiquitin protein ligase activity | GO:0061630 | 24 | 286 | 1.73E-05 | 0.000449883 |
| cell-cell adhesion | GO:0098609 | 16 | 142 | 1.75E-05 | 0.000449883 |
| spinal cord development | GO:0021510 | 8 | 32 | 1.75E-05 | 0.000449883 |
| neuromuscular junction development | GO:0007528 | 8 | 32 | 1.75E-05 | 0.000449883 |
| protein polyubiquitination | GO:0000209 | 23 | 267 | 1.75E-05 | 0.000449883 |
| insulin receptor binding | GO:0005158 | 7 | 23 | 2.03E-05 | 0.000517398 |
| integrin-mediated signaling pathway | GO:0007229 | 13 | 97 | 2.06E-05 | 0.000522704 |
| peptidyl-serine phosphorylation | GO:0018105 | 17 | 161 | 2.08E-05 | 0.00052519 |
| histone deacetylase complex | GO:0000118 | 9 | 44 | 2.11E-05 | 0.000530217 |
| inorganic cation transmembrane transport | GO:0098662 | 5 | 8 | 2.24E-05 | 0.000560163 |
| node of Ranvier | GO:0033268 | 6 | 15 | 2.30E-05 | 0.00057058 |
| canonical Wnt signaling pathway | GO:0060070 | 11 | 70 | 2.38E-05 | 0.000588107 |
| Schaffer collateral - CA1 synapse | GO:0098685 | 12 | 84 | 2.41E-05 | 0.000592397 |
| positive regulation of glycolytic process | GO:0045821 | 7 | 24 | 2.55E-05 | 0.00062077 |
| decidualization | GO:0046697 | 7 | 24 | 2.55E-05 | 0.00062077 |
| membrane raft | GO:0045121 | 21 | 237 | 2.73E-05 | 0.000660534 |
| neural crest cell migration | GO:0001755 | 9 | 46 | 2.87E-05 | 0.000692068 |
| structural constituent of cytoskeleton | GO:0005200 | 13 | 101 | 3.02E-05 | 0.000723821 |
| synaptic vesicle exocytosis | GO:0016079 | 7 | 25 | 3.18E-05 | 0.000750984 |
| synaptic membrane adhesion | GO:0099560 | 7 | 25 | 3.18E-05 | 0.000750984 |
| positive regulation of lamellipodium assembly | GO:0010592 | 7 | 25 | 3.18E-05 | 0.000750984 |
| vesicle-mediated transport | GO:0016192 | 16 | 150 | 3.22E-05 | 0.000751754 |
| regulation of cell shape | GO:0008360 | 16 | 150 | 3.22E-05 | 0.000751754 |
| endosome to lysosome transport | GO:0008333 | 9 | 47 | 3.34E-05 | 0.00077521 |
| response to drug | GO:0042493 | 23 | 280 | 3.49E-05 | 0.000807839 |
| positive regulation of proteasomal ubiquitin-dependent protein catabolic process | GO:0032436 | 11 | 74 | 3.77E-05 | 0.000866177 |
| adult locomotory behavior | GO:0008344 | 9 | 48 | 3.86E-05 | 0.000883574 |
| clathrin-coated vesicle | GO:0030136 | 10 | 61 | 4.03E-05 | 0.000912624 |
| positive regulation of MAP kinase activity | GO:0043406 | 10 | 61 | 4.03E-05 | 0.000912624 |
| Wnt signaling pathway | GO:0016055 | 18 | 189 | 4.24E-05 | 0.000955635 |
| phosphatidylinositol 3-kinase signaling | GO:0014065 | 8 | 37 | 4.30E-05 | 0.000965095 |
| vasculogenesis | GO:0001570 | 10 | 62 | 4.55E-05 | 0.001017388 |
| cytoskeleton organization | GO:0007010 | 15 | 138 | 4.62E-05 | 0.001026861 |
| protein-macromolecule adaptor activity | GO:0030674 | 11 | 76 | 4.69E-05 | 0.001032247 |
| caveola | GO:0005901 | 11 | 76 | 4.69E-05 | 0.001032247 |
| endoplasmic reticulum membrane | GO:0005789 | 52 | 942 | 4.76E-05 | 0.001039049 |
| metalloendopeptidase activity | GO:0004222 | 13 | 106 | 4.76E-05 | 0.001039049 |
| regulation of small GTPase mediated signal transduction | GO:0051056 | 15 | 139 | 4.98E-05 | 0.00106604 |
| intermediate filament binding | GO:0019215 | 5 | 10 | 4.98E-05 | 0.00106604 |
| regulation of bone resorption | GO:0045124 | 5 | 10 | 4.98E-05 | 0.00106604 |
| negative regulation of gene expression | GO:0010629 | 19 | 210 | 4.99E-05 | 0.00106604 |
| transcription coactivator activity | GO:0003713 | 21 | 248 | 5.02E-05 | 0.00106604 |
| chemical synaptic transmission | GO:0007268 | 21 | 248 | 5.02E-05 | 0.00106604 |
| neuron development | GO:0048666 | 9 | 50 | 5.12E-05 | 0.001071084 |
| epidermal growth factor receptor signaling pathway | GO:0007173 | 9 | 50 | 5.12E-05 | 0.001071084 |
| SNARE complex | GO:0031201 | 9 | 50 | 5.12E-05 | 0.001071084 |
| activation of MAPK activity | GO:0000187 | 14 | 123 | 5.20E-05 | 0.001073875 |
| potassium ion transmembrane transport | GO:0071805 | 14 | 123 | 5.20E-05 | 0.001073875 |
| transmembrane receptor protein tyrosine kinase signaling pathway | GO:0007169 | 14 | 123 | 5.20E-05 | 0.001073875 |
| myelination in peripheral nervous system | GO:0022011 | 6 | 18 | 5.28E-05 | 0.001086299 |
| mRNA processing | GO:0006397 | 16 | 158 | 5.70E-05 | 0.001167245 |
| negative regulation of canonical Wnt signaling pathway | GO:0090090 | 17 | 176 | 5.83E-05 | 0.001188008 |
| social behavior | GO:0035176 | 9 | 51 | 5.86E-05 | 0.001188352 |
| basement membrane | GO:0005604 | 12 | 93 | 5.92E-05 | 0.001188352 |
| male gonad development | GO:0008584 | 12 | 93 | 5.92E-05 | 0.001188352 |
| chondrocyte differentiation | GO:0002062 | 8 | 39 | 5.96E-05 | 0.001188352 |
| GTP binding | GO:0005525 | 27 | 373 | 5.96E-05 | 0.001188352 |
| biological_process | GO:0008150 | 37 | 594 | 6.06E-05 | 0.001203307 |
| peptidyl-threonine phosphorylation | GO:0018107 | 10 | 65 | 6.51E-05 | 0.001286392 |
| DNA-binding transcription repressor activity, RNA polymerase II-specific | GO:0001227 | 21 | 253 | 6.55E-05 | 0.00128907 |
| autophagy | GO:0006914 | 14 | 126 | 6.60E-05 | 0.001293173 |
| positive regulation of stress fiber assembly | GO:0051496 | 9 | 52 | 6.70E-05 | 0.001308714 |
| TOR signaling | GO:0031929 | 6 | 19 | 6.78E-05 | 0.001318212 |
| motor activity | GO:0003774 | 8 | 40 | 6.96E-05 | 0.001348587 |
| myelin sheath | GO:0043209 | 7 | 29 | 7.13E-05 | 0.001369262 |
| protein ubiquitination | GO:0016567 | 32 | 486 | 7.15E-05 | 0.001369262 |
| cytoplasmic vesicle membrane | GO:0030659 | 15 | 144 | 7.19E-05 | 0.001369262 |
| sensory perception of sound | GO:0007605 | 15 | 144 | 7.19E-05 | 0.001369262 |
| activation of protein kinase activity | GO:0032147 | 10 | 66 | 7.30E-05 | 0.001383952 |
| protein localization to juxtaparanode region of axon | GO:0071205 | 4 | 5 | 8.15E-05 | 0.00153049 |
| mammary gland duct morphogenesis | GO:0060603 | 4 | 5 | 8.15E-05 | 0.00153049 |
| post-embryonic development | GO:0009791 | 10 | 67 | 8.17E-05 | 0.00153049 |
| negative regulation of smooth muscle cell proliferation | GO:0048662 | 7 | 30 | 8.57E-05 | 0.001597833 |
| histone H3-K4 trimethylation | GO:0080182 | 6 | 20 | 8.60E-05 | 0.001597833 |
| cytoskeletal protein binding | GO:0008092 | 9 | 54 | 8.68E-05 | 0.001607544 |
| regulation of cell growth | GO:0001558 | 10 | 68 | 9.13E-05 | 0.001682867 |
| histone deacetylase binding | GO:0042826 | 13 | 114 | 9.36E-05 | 0.001719643 |
| post-translational protein modification | GO:0043687 | 25 | 342 | 9.52E-05 | 0.001742293 |
| regulation of signal transduction by p53 class mediator | GO:1901796 | 14 | 131 | 9.65E-05 | 0.0017476 |
| actin cytoskeleton organization | GO:0030036 | 16 | 166 | 9.73E-05 | 0.0017476 |
| positive regulation of protein kinase B signaling | GO:0051897 | 16 | 166 | 9.73E-05 | 0.0017476 |
| mitochondrial inner membrane | GO:0005743 | 27 | 385 | 9.76E-05 | 0.0017476 |
| hepatocyte growth factor receptor signaling pathway | GO:0048012 | 5 | 12 | 9.78E-05 | 0.0017476 |
| cardiac myofibril assembly | GO:0055003 | 5 | 12 | 9.78E-05 | 0.0017476 |
| cell fate commitment | GO:0045165 | 9 | 55 | 9.84E-05 | 0.00175265 |
| regulation of neuron projection development | GO:0010975 | 7 | 31 | 0.00010246 | 0.001817238 |
| muscle cell cellular homeostasis | GO:0046716 | 6 | 21 | 0.00010779 | 0.001897419 |
| intermediate filament cytoskeleton organization | GO:0045104 | 6 | 21 | 0.00010779 | 0.001897419 |
| structural constituent of muscle | GO:0008307 | 8 | 43 | 0.00010878 | 0.001907562 |
| protein homodimerization activity | GO:0042803 | 39 | 660 | 0.00011094 | 0.001923065 |
| mitotic spindle | GO:0072686 | 12 | 100 | 0.0001112 | 0.001923065 |
| substrate adhesion-dependent cell spreading | GO:0034446 | 9 | 56 | 0.00011132 | 0.001923065 |
| cellular response to transforming growth factor beta stimulus | GO:0071560 | 9 | 56 | 0.00011132 | 0.001923065 |
| GABA-ergic synapse | GO:0098982 | 10 | 70 | 0.0001133 | 0.001949983 |
| regulation of presynapse assembly | GO:1905606 | 7 | 32 | 0.00012173 | 0.002087453 |
| regulation of GTPase activity | GO:0043087 | 10 | 71 | 0.0001259 | 0.002151019 |
| nuclear body | GO:0016604 | 23 | 307 | 0.00012731 | 0.00216648 |
| PDZ domain binding | GO:0030165 | 11 | 86 | 0.00012774 | 0.00216648 |
| actin filament organization | GO:0007015 | 13 | 118 | 0.00012844 | 0.002170442 |
| insulin-like growth factor receptor signaling pathway | GO:0048009 | 5 | 13 | 0.00013213 | 0.002224636 |
| cell-cell junction organization | GO:0045216 | 6 | 22 | 0.00013381 | 0.002244814 |
| positive regulation of kinase activity | GO:0033674 | 10 | 72 | 0.00013967 | 0.002326406 |
| cellular response to starvation | GO:0009267 | 10 | 72 | 0.00013967 | 0.002326406 |
| bone mineralization | GO:0030282 | 8 | 45 | 0.00014381 | 0.002386721 |
| vesicle | GO:0031982 | 16 | 173 | 0.00015119 | 0.002500362 |
| ribonucleoprotein complex | GO:1990904 | 15 | 156 | 0.00016276 | 0.002682072 |
| wound healing | GO:0042060 | 11 | 89 | 0.00016802 | 0.002757557 |
| lung alveolus development | GO:0048286 | 7 | 34 | 0.00016912 | 0.002757557 |
| long-term memory | GO:0007616 | 7 | 34 | 0.00016912 | 0.002757557 |
| transcription corepressor activity | GO:0003714 | 17 | 194 | 0.00017354 | 0.002819731 |
| ATPase-coupled cation transmembrane transporter activity | GO:0019829 | 5 | 14 | 0.00017501 | 0.002833631 |
| ubiquitin protein ligase binding | GO:0031625 | 22 | 294 | 0.00017941 | 0.002894792 |
| regulation of actin cytoskeleton organization | GO:0032956 | 10 | 75 | 0.00018884 | 0.003036389 |
| postsynaptic membrane | GO:0045211 | 14 | 141 | 0.00019598 | 0.003140418 |
| positive regulation of synapse assembly | GO:0051965 | 9 | 61 | 0.00019884 | 0.003164351 |
| negative regulation of neuron projection development | GO:0010977 | 9 | 61 | 0.00019884 | 0.003164351 |
| neurofilament cytoskeleton organization | GO:0060052 | 4 | 7 | 0.00020356 | 0.003225772 |
| positive regulation of protein phosphorylation | GO:0001934 | 16 | 178 | 0.00020408 | 0.003225772 |
| phosphatidylinositol biosynthetic process | GO:0006661 | 10 | 76 | 0.00020815 | 0.003278887 |
| DNA binding | GO:0003677 | 60 | 1209 | 0.00021132 | 0.003317619 |
| Rho guanyl-nucleotide exchange factor activity | GO:0005089 | 8 | 48 | 0.00021335 | 0.003338224 |
| cellular response to tumor necrosis factor | GO:0071356 | 13 | 125 | 0.00021646 | 0.003375396 |
| neuron projection morphogenesis | GO:0048812 | 9 | 62 | 0.00022185 | 0.003448027 |
| phospholipid binding | GO:0005543 | 12 | 109 | 0.00023195 | 0.003592864 |
| postsynapse | GO:0098794 | 11 | 93 | 0.00023807 | 0.003675437 |
| SMAD binding | GO:0046332 | 8 | 49 | 0.00024188 | 0.00372198 |
| mitochondrial intermembrane space | GO:0005758 | 10 | 78 | 0.00025178 | 0.003858855 |
| histone binding | GO:0042393 | 15 | 163 | 0.00025244 | 0.003858855 |
| vesicle fusion | GO:0006906 | 7 | 37 | 0.00026714 | 0.004070101 |
| positive regulation of neuron projection development | GO:0010976 | 12 | 111 | 0.00027032 | 0.004105098 |
| skeletal muscle tissue development | GO:0007519 | 8 | 50 | 0.00027346 | 0.004125942 |
| negative regulation of autophagy | GO:0010507 | 8 | 50 | 0.00027346 | 0.004125942 |
| SNARE binding | GO:0000149 | 9 | 64 | 0.00027456 | 0.004129002 |
| mRNA 3'-UTR AU-rich region binding | GO:0035925 | 6 | 26 | 0.00029131 | 0.004331177 |
| G-protein alpha-subunit binding | GO:0001965 | 6 | 26 | 0.00029131 | 0.004331177 |
| syntaxin-1 binding | GO:0017075 | 6 | 26 | 0.00029131 | 0.004331177 |
| ATP-dependent microtubule motor activity, plus-end-directed | GO:0008574 | 5 | 16 | 0.00029173 | 0.004331177 |
| MOZ/MORF histone acetyltransferase complex | GO:0070776 | 4 | 8 | 0.00029827 | 0.004372487 |
| synaptic vesicle clustering | GO:0097091 | 4 | 8 | 0.00029827 | 0.004372487 |
| sodium channel inhibitor activity | GO:0019871 | 4 | 8 | 0.00029827 | 0.004372487 |
| paranodal junction | GO:0033010 | 4 | 8 | 0.00029827 | 0.004372487 |
| centrosome | GO:0005813 | 31 | 506 | 0.00030254 | 0.004411191 |
| phosphatidylinositol binding | GO:0035091 | 10 | 80 | 0.00030281 | 0.004411191 |
| anterior/posterior pattern specification | GO:0009952 | 11 | 96 | 0.00030554 | 0.004437062 |
| synapse organization | GO:0050808 | 8 | 51 | 0.00030833 | 0.004451104 |
| protein O-linked glycosylation | GO:0006493 | 7 | 38 | 0.00030843 | 0.004451104 |
| transcription regulator complex | GO:0005667 | 18 | 226 | 0.00033647 | 0.004840768 |
| ephrin receptor binding | GO:0046875 | 6 | 27 | 0.0003473 | 0.004920374 |
| negative regulation of JNK cascade | GO:0046329 | 6 | 27 | 0.0003473 | 0.004920374 |
| associative learning | GO:0008306 | 6 | 27 | 0.0003473 | 0.004920374 |
| focal adhesion assembly | GO:0048041 | 6 | 27 | 0.0003473 | 0.004920374 |
| voltage-gated calcium channel complex | GO:0005891 | 6 | 27 | 0.0003473 | 0.004920374 |
| regulation of heart rate by cardiac conduction | GO:0086091 | 7 | 39 | 0.00035468 | 0.004994568 |
| heart morphogenesis | GO:0003007 | 7 | 39 | 0.00035468 | 0.004994568 |
| cellular calcium ion homeostasis | GO:0006874 | 11 | 98 | 0.00035894 | 0.005039188 |
| Golgi organization | GO:0007030 | 12 | 115 | 0.00036339 | 0.005051029 |
| glucose homeostasis | GO:0042593 | 12 | 115 | 0.00036339 | 0.005051029 |
| DNA-binding transcription activator activity, RNA polymerase II-specific | GO:0001228 | 29 | 466 | 0.0003673 | 0.005051029 |
| axon initial segment | GO:0043194 | 5 | 17 | 0.00036847 | 0.005051029 |
| voltage-gated sodium channel complex | GO:0001518 | 5 | 17 | 0.00036847 | 0.005051029 |
| myosin V binding | GO:0031489 | 5 | 17 | 0.00036847 | 0.005051029 |
| apical dendrite | GO:0097440 | 5 | 17 | 0.00036847 | 0.005051029 |
| positive regulation of protein localization to cell surface | GO:2000010 | 5 | 17 | 0.00036847 | 0.005051029 |
| DNA-binding transcription factor activity | GO:0003700 | 35 | 607 | 0.0003727 | 0.005060668 |
| microtubule motor activity | GO:0003777 | 9 | 67 | 0.00037271 | 0.005060668 |
| cellular response to retinoic acid | GO:0071300 | 9 | 67 | 0.00037271 | 0.005060668 |
| lipid binding | GO:0008289 | 14 | 151 | 0.00037353 | 0.005060668 |
| regulation of membrane potential | GO:0042391 | 11 | 99 | 0.00038844 | 0.005247318 |
| lung development | GO:0030324 | 10 | 83 | 0.00039536 | 0.005325341 |
| extracellular matrix binding | GO:0050840 | 6 | 28 | 0.00041135 | 0.00552475 |
| calcium-release channel activity | GO:0015278 | 4 | 9 | 0.00042088 | 0.005620133 |
| response to fluid shear stress | GO:0034405 | 4 | 9 | 0.00042088 | 0.005620133 |
| multicellular organism growth | GO:0035264 | 10 | 84 | 0.000431 | 0.005738848 |
| phosphorylation | GO:0016310 | 11 | 101 | 0.00045358 | 0.006022211 |
| retinal ganglion cell axon guidance | GO:0031290 | 5 | 18 | 0.00045954 | 0.006049561 |
| camera-type eye morphogenesis | GO:0048593 | 5 | 18 | 0.00045954 | 0.006049561 |
| endocardial cushion morphogenesis | GO:0003203 | 5 | 18 | 0.00045954 | 0.006049561 |
| transport vesicle membrane | GO:0030658 | 7 | 41 | 0.00046381 | 0.006088494 |
| ubiquitin-protein transferase activity | GO:0004842 | 18 | 233 | 0.00047161 | 0.006173474 |
| cation transport | GO:0006812 | 6 | 29 | 0.00048427 | 0.006286054 |
| axonal growth cone | GO:0044295 | 6 | 29 | 0.00048427 | 0.006286054 |
| branching involved in blood vessel morphogenesis | GO:0001569 | 6 | 29 | 0.00048427 | 0.006286054 |
| phosphatidylinositol-mediated signaling | GO:0048015 | 8 | 55 | 0.00048596 | 0.006290427 |
| regulation of macroautophagy | GO:0016241 | 9 | 70 | 0.00049812 | 0.006429914 |
| estrogen receptor binding | GO:0030331 | 7 | 42 | 0.00052759 | 0.006791471 |
| oxidation-reduction process | GO:0055114 | 31 | 525 | 0.00054219 | 0.006960053 |
| molecular adaptor activity | GO:0060090 | 9 | 71 | 0.0005469 | 0.007001244 |
| erythrocyte development | GO:0048821 | 5 | 19 | 0.00056657 | 0.007117805 |
| costamere | GO:0043034 | 5 | 19 | 0.00056657 | 0.007117805 |
| cellular response to peptide hormone stimulus | GO:0071375 | 5 | 19 | 0.00056657 | 0.007117805 |
| regulation of neurotransmitter secretion | GO:0046928 | 5 | 19 | 0.00056657 | 0.007117805 |
| reactive oxygen species metabolic process | GO:0072593 | 6 | 30 | 0.00056686 | 0.007117805 |
| platelet-derived growth factor receptor signaling pathway | GO:0048008 | 6 | 30 | 0.00056686 | 0.007117805 |
| activation of GTPase activity | GO:0090630 | 11 | 104 | 0.00056826 | 0.007117805 |
| protein N-terminus binding | GO:0047485 | 11 | 104 | 0.00056826 | 0.007117805 |
| chloride channel inhibitor activity | GO:0019869 | 4 | 10 | 0.00057561 | 0.007171215 |
| positive regulation of synaptic plasticity | GO:0031915 | 4 | 10 | 0.00057561 | 0.007171215 |
| bicellular tight junction | GO:0005923 | 12 | 122 | 0.00059149 | 0.007349375 |
| regulation of G protein-coupled receptor signaling pathway | GO:0008277 | 7 | 43 | 0.00059816 | 0.007392669 |
| regulation of RNA splicing | GO:0043484 | 7 | 43 | 0.00059816 | 0.007392669 |
| Rac GTPase binding | GO:0048365 | 8 | 57 | 0.00060167 | 0.00741638 |
| positive regulation of protein catabolic process | GO:0045732 | 9 | 73 | 0.00065622 | 0.008046057 |
| tertiary granule membrane | GO:0070821 | 9 | 73 | 0.00065622 | 0.008046057 |
| regulation of exocytosis | GO:0017157 | 6 | 31 | 0.00065999 | 0.008069948 |
| mitochondrial matrix | GO:0005759 | 24 | 370 | 0.00066164 | 0.008069948 |
| promoter-specific chromatin binding | GO:1990841 | 8 | 58 | 0.00066734 | 0.008118148 |
| mRNA binding | GO:0003729 | 16 | 200 | 0.00067204 | 0.008153977 |
| platelet degranulation | GO:0002576 | 12 | 124 | 0.0006753 | 0.008172219 |
| cadherin binding | GO:0045296 | 21 | 305 | 0.00069069 | 0.00833674 |
| homophilic cell adhesion via plasma membrane adhesion molecules | GO:0007156 | 14 | 162 | 0.00071246 | 0.008577147 |
| negative regulation of cell growth | GO:0030308 | 12 | 125 | 0.0007208 | 0.008655191 |
| negative regulation of neuron differentiation | GO:0045665 | 8 | 59 | 0.00073866 | 0.008846818 |
| T-tubule | GO:0030315 | 7 | 45 | 0.00076168 | 0.009067049 |
| mitochondrion organization | GO:0007005 | 10 | 91 | 0.00076274 | 0.009067049 |
| positive regulation of erythrocyte differentiation | GO:0045648 | 6 | 32 | 0.00076452 | 0.009067049 |
| messenger ribonucleoprotein complex | GO:1990124 | 4 | 11 | 0.00076681 | 0.009067049 |
| synaptic vesicle maturation | GO:0016188 | 4 | 11 | 0.00076681 | 0.009067049 |
| regulation of alternative mRNA splicing, via spliceosome | GO:0000381 | 9 | 75 | 0.00078276 | 0.00920879 |
| calcium ion transport | GO:0006816 | 9 | 75 | 0.00078276 | 0.00920879 |
| negative regulation of epithelial cell proliferation | GO:0050680 | 8 | 60 | 0.00081599 | 0.009527385 |
| cellular protein localization | GO:0034613 | 8 | 60 | 0.00081599 | 0.009527385 |
| syntaxin binding | GO:0019905 | 8 | 60 | 0.00081599 | 0.009527385 |
| positive regulation of dendrite morphogenesis | GO:0050775 | 5 | 21 | 0.00083539 | 0.009632855 |
| regulation of dendrite morphogenesis | GO:0048814 | 5 | 21 | 0.00083539 | 0.009632855 |
| regulation of neuron death | GO:1901214 | 5 | 21 | 0.00083539 | 0.009632855 |
| adrenal gland development | GO:0030325 | 5 | 21 | 0.00083539 | 0.009632855 |
| positive regulation of endocytosis | GO:0045807 | 5 | 21 | 0.00083539 | 0.009632855 |
| cell body | GO:0044297 | 9 | 76 | 0.00085309 | 0.009788417 |
| liver development | GO:0001889 | 9 | 76 | 0.00085309 | 0.009788417 |
| lactation | GO:0007595 | 7 | 46 | 0.00085571 | 0.009794263 |
| positive regulation of macroautophagy | GO:0016239 | 6 | 33 | 0.00088139 | 0.009989826 |
| autophagosome maturation | GO:0097352 | 6 | 33 | 0.00088139 | 0.009989826 |
| extrinsic apoptotic signaling pathway via death domain receptors | GO:0008625 | 6 | 33 | 0.00088139 | 0.009989826 |
| odontogenesis | GO:0042476 | 6 | 33 | 0.00088139 | 0.009989826 |
| mitochondrial outer membrane | GO:0005741 | 14 | 166 | 0.00088767 | 0.010026429 |
| trans-Golgi network membrane | GO:0032588 | 10 | 93 | 0.00088894 | 0.010026429 |
| ficolin-1-rich granule membrane | GO:0101003 | 8 | 61 | 0.00089968 | 0.010123041 |
| sodium ion transport | GO:0006814 | 9 | 77 | 0.00092847 | 0.010421768 |
| lysosomal lumen | GO:0043202 | 10 | 94 | 0.00095818 | 0.01070875 |
| negative regulation of BMP signaling pathway | GO:0030514 | 7 | 47 | 0.00095865 | 0.01070875 |
| regulation of smooth muscle contraction | GO:0006940 | 4 | 12 | 0.00099883 | 0.0109423 |
| calcineurin-NFAT signaling cascade | GO:0033173 | 4 | 12 | 0.00099883 | 0.0109423 |
| semaphorin-plexin signaling pathway involved in axon guidance | GO:1902287 | 4 | 12 | 0.00099883 | 0.0109423 |
| cellular response to vitamin D | GO:0071305 | 4 | 12 | 0.00099883 | 0.0109423 |
| calcium ion transport into cytosol | GO:0060402 | 4 | 12 | 0.00099883 | 0.0109423 |
| myoblast fusion | GO:0007520 | 5 | 22 | 0.00100075 | 0.0109423 |
| membrane depolarization | GO:0051899 | 5 | 22 | 0.00100075 | 0.0109423 |
| calmodulin-dependent protein kinase activity | GO:0004683 | 5 | 22 | 0.00100075 | 0.0109423 |
| translation factor activity, RNA binding | GO:0008135 | 5 | 22 | 0.00100075 | 0.0109423 |
| negative regulation of endopeptidase activity | GO:0010951 | 13 | 149 | 0.00100357 | 0.010947353 |
| myosin complex | GO:0016459 | 6 | 34 | 0.00101155 | 0.010957233 |
| renal water homeostasis | GO:0003091 | 6 | 34 | 0.00101155 | 0.010957233 |
| phosphatidylcholine biosynthetic process | GO:0006656 | 6 | 34 | 0.00101155 | 0.010957233 |
| ion transmembrane transport | GO:0034220 | 16 | 209 | 0.00103761 | 0.011213388 |
| SH3 domain binding | GO:0017124 | 12 | 131 | 0.00105096 | 0.011331329 |
| collagen fibril organization | GO:0030199 | 7 | 48 | 0.00107109 | 0.011495017 |
| erythrocyte differentiation | GO:0030218 | 7 | 48 | 0.00107109 | 0.011495017 |
| cellular response to mechanical stimulus | GO:0071260 | 9 | 79 | 0.00109546 | 0.011729446 |
| SNAP receptor activity | GO:0005484 | 6 | 35 | 0.00115596 | 0.012348857 |
| positive regulation of dendrite extension | GO:1903861 | 5 | 23 | 0.00118921 | 0.012625729 |
| fatty acid metabolic process | GO:0006631 | 8 | 64 | 0.00119275 | 0.012625729 |
| vascular endothelial growth factor receptor signaling pathway | GO:0048010 | 8 | 64 | 0.00119275 | 0.012625729 |
| smoothened signaling pathway | GO:0007224 | 8 | 64 | 0.00119275 | 0.012625729 |
| peptide hormone secretion | GO:0030072 | 3 | 5 | 0.00125056 | 0.01300074 |
| histone acetyltransferase activity (H3-K23 specific) | GO:0043994 | 3 | 5 | 0.00125056 | 0.01300074 |
| histone methyltransferase activity (H3-K27 specific) | GO:0046976 | 3 | 5 | 0.00125056 | 0.01300074 |
| neurofibrillary tangle | GO:0097418 | 3 | 5 | 0.00125056 | 0.01300074 |
| retrograde neuronal dense core vesicle transport | GO:1990049 | 3 | 5 | 0.00125056 | 0.01300074 |
| establishment of spindle orientation | GO:0051294 | 3 | 5 | 0.00125056 | 0.01300074 |
| histone H3-K23 acetylation | GO:0043972 | 3 | 5 | 0.00125056 | 0.01300074 |
| regulation of calcineurin-NFAT signaling cascade | GO:0070884 | 3 | 5 | 0.00125056 | 0.01300074 |
| detection of mechanical stimulus | GO:0050982 | 4 | 13 | 0.00127608 | 0.01303287 |
| co-receptor binding | GO:0039706 | 4 | 13 | 0.00127608 | 0.01303287 |
| dopamine metabolic process | GO:0042417 | 4 | 13 | 0.00127608 | 0.01303287 |
| mechanosensitive ion channel activity | GO:0008381 | 4 | 13 | 0.00127608 | 0.01303287 |
| removal of superoxide radicals | GO:0019430 | 4 | 13 | 0.00127608 | 0.01303287 |
| inositol 1,4,5 trisphosphate binding | GO:0070679 | 4 | 13 | 0.00127608 | 0.01303287 |
| HOPS complex | GO:0030897 | 4 | 13 | 0.00127608 | 0.01303287 |
| somatodendritic compartment | GO:0036477 | 4 | 13 | 0.00127608 | 0.01303287 |
| microtubule-based movement | GO:0007018 | 9 | 81 | 0.00128592 | 0.013075857 |
| cellular protein modification process | GO:0006464 | 9 | 81 | 0.00128592 | 0.013075857 |
| regulation of gene expression | GO:0010468 | 17 | 235 | 0.00130929 | 0.013284447 |
| positive regulation of tumor necrosis factor production | GO:0032760 | 7 | 50 | 0.00132686 | 0.013404208 |
| regulation of synaptic vesicle exocytosis | GO:2000300 | 7 | 50 | 0.00132686 | 0.013404208 |
| trans-Golgi network | GO:0005802 | 14 | 174 | 0.0013483 | 0.013591206 |
| response to toxic substance | GO:0009636 | 9 | 82 | 0.00139068 | 0.013988107 |
| protein kinase A binding | GO:0051018 | 5 | 24 | 0.00140268 | 0.01404799 |
| actin-dependent ATPase activity | GO:0030898 | 5 | 24 | 0.00140268 | 0.01404799 |
| circadian regulation of gene expression | GO:0032922 | 8 | 66 | 0.00142711 | 0.01414028 |
| chromatin DNA binding | GO:0031490 | 8 | 66 | 0.00142711 | 0.01414028 |
| hematopoietic progenitor cell differentiation | GO:0002244 | 8 | 66 | 0.00142711 | 0.01414028 |
| roof of mouth development | GO:0060021 | 8 | 66 | 0.00142711 | 0.01414028 |
| p53 binding | GO:0002039 | 8 | 66 | 0.00142711 | 0.01414028 |
| integral component of postsynaptic density membrane | GO:0099061 | 7 | 51 | 0.00147145 | 0.01454855 |
| intrinsic component of plasma membrane | GO:0031226 | 6 | 37 | 0.00149163 | 0.014685603 |
| hair follicle development | GO:0001942 | 6 | 37 | 0.00149163 | 0.014685603 |
| cellular response to hydrogen peroxide | GO:0070301 | 8 | 67 | 0.00155725 | 0.015297289 |
| RNA polymerase II transcription regulatory region sequence-specific DNA binding | GO:0000977 | 21 | 327 | 0.00156034 | 0.015297289 |
| response to oxidative stress | GO:0006979 | 10 | 101 | 0.00157664 | 0.015424478 |
| cellular response to nutrient levels | GO:0031669 | 4 | 14 | 0.00160293 | 0.015486072 |
| negative regulation of necroptotic process | GO:0060546 | 4 | 14 | 0.00160293 | 0.015486072 |
| negative regulation of calcium ion transport | GO:0051926 | 4 | 14 | 0.00160293 | 0.015486072 |
| pre-miRNA processing | GO:0031054 | 4 | 14 | 0.00160293 | 0.015486072 |
| ruffle assembly | GO:0097178 | 4 | 14 | 0.00160293 | 0.015486072 |
| calcium activated cation channel activity | GO:0005227 | 4 | 14 | 0.00160293 | 0.015486072 |
| cellular protein metabolic process | GO:0044267 | 15 | 198 | 0.00161466 | 0.015567026 |
| transport across blood-brain barrier | GO:0150104 | 9 | 84 | 0.0016208 | 0.015593862 |
| positive regulation of fat cell differentiation | GO:0045600 | 7 | 52 | 0.00162802 | 0.01559879 |
| ribosome binding | GO:0043022 | 7 | 52 | 0.00162802 | 0.01559879 |
| microtubule cytoskeleton | GO:0015630 | 13 | 158 | 0.00163928 | 0.015646419 |
| retina layer formation | GO:0010842 | 5 | 25 | 0.00164309 | 0.015646419 |
| cell aging | GO:0007569 | 5 | 25 | 0.00164309 | 0.015646419 |
| sarcomere organization | GO:0045214 | 6 | 38 | 0.00168497 | 0.015899147 |
| positive regulation of glucose import | GO:0046326 | 6 | 38 | 0.00168497 | 0.015899147 |
| voltage-gated calcium channel activity | GO:0005245 | 6 | 38 | 0.00168497 | 0.015899147 |
| frizzled binding | GO:0005109 | 6 | 38 | 0.00168497 | 0.015899147 |
| platelet activation | GO:0030168 | 10 | 102 | 0.00168674 | 0.015899147 |
| synaptic vesicle membrane | GO:0030672 | 8 | 68 | 0.00169661 | 0.015959777 |
| cellular response to cAMP | GO:0071320 | 7 | 53 | 0.00179725 | 0.016838307 |
| cortical actin cytoskeleton | GO:0030864 | 7 | 53 | 0.00179725 | 0.016838307 |
| negative regulation of protein targeting to mitochondrion | GO:1903215 | 3 | 6 | 0.0018351 | 0.01685322 |
| positive regulation of intrinsic apoptotic signaling pathway in response to DNA damage | GO:1902231 | 3 | 6 | 0.0018351 | 0.01685322 |
| negative regulation of sodium ion transport | GO:0010766 | 3 | 6 | 0.0018351 | 0.01685322 |
| histone H3-K27 methylation | GO:0070734 | 3 | 6 | 0.0018351 | 0.01685322 |
| neurotrophin TRKA receptor binding | GO:0005168 | 3 | 6 | 0.0018351 | 0.01685322 |
| ATP-activated inward rectifier potassium channel activity | GO:0015272 | 3 | 6 | 0.0018351 | 0.01685322 |
| RNA polymerase III type 3 promoter sequence-specific DNA binding | GO:0001006 | 3 | 6 | 0.0018351 | 0.01685322 |
| peptide cross-linking via chondroitin 4-sulfate glycosaminoglycan | GO:0019800 | 3 | 6 | 0.0018351 | 0.01685322 |
| intrinsic component of the cytoplasmic side of the plasma membrane | GO:0031235 | 3 | 6 | 0.0018351 | 0.01685322 |
| peripheral nervous system axon regeneration | GO:0014012 | 3 | 6 | 0.0018351 | 0.01685322 |
| positive regulation of peptidyl-tyrosine phosphorylation | GO:0050731 | 9 | 86 | 0.00188047 | 0.017189466 |
| apoptotic process | GO:0006915 | 30 | 546 | 0.00189431 | 0.017189466 |
| histone deacetylation | GO:0016575 | 6 | 39 | 0.00189675 | 0.017189466 |
| negative regulation of G0 to G1 transition | GO:0070317 | 6 | 39 | 0.00189675 | 0.017189466 |
| presynaptic active zone | GO:0048786 | 6 | 39 | 0.00189675 | 0.017189466 |
| long-chain fatty acid biosynthetic process | GO:0042759 | 5 | 26 | 0.00191241 | 0.017189466 |
| protein catabolic process | GO:0030163 | 5 | 26 | 0.00191241 | 0.017189466 |
| positive regulation of microtubule polymerization | GO:0031116 | 5 | 26 | 0.00191241 | 0.017189466 |
| response to electrical stimulus | GO:0051602 | 5 | 26 | 0.00191241 | 0.017189466 |
| chondroitin sulfate biosynthetic process | GO:0030206 | 5 | 26 | 0.00191241 | 0.017189466 |
| positive regulation of muscle cell differentiation | GO:0051149 | 5 | 26 | 0.00191241 | 0.017189466 |
| cell differentiation | GO:0030154 | 32 | 596 | 0.00192606 | 0.017278811 |
| response to ischemia | GO:0002931 | 7 | 54 | 0.0019798 | 0.017360226 |
| translation repressor activity, mRNA regulatory element binding | GO:0000900 | 4 | 15 | 0.00198371 | 0.017360226 |
| muscle alpha-actinin binding | GO:0051371 | 4 | 15 | 0.00198371 | 0.017360226 |
| C2H2 zinc finger domain binding | GO:0070742 | 4 | 15 | 0.00198371 | 0.017360226 |
| pre-mRNA binding | GO:0036002 | 4 | 15 | 0.00198371 | 0.017360226 |
| regulation of protein kinase A signaling | GO:0010738 | 4 | 15 | 0.00198371 | 0.017360226 |
| negative regulation of intrinsic apoptotic signaling pathway in response to DNA damage | GO:1902230 | 4 | 15 | 0.00198371 | 0.017360226 |
| alpha-actinin binding | GO:0051393 | 4 | 15 | 0.00198371 | 0.017360226 |
| sympathetic nervous system development | GO:0048485 | 4 | 15 | 0.00198371 | 0.017360226 |
| positive regulation of nuclear-transcribed mRNA catabolic process, deadenylation-dependent decay | GO:1900153 | 4 | 15 | 0.00198371 | 0.017360226 |
| nuclear migration | GO:0007097 | 4 | 15 | 0.00198371 | 0.017360226 |
| synaptic vesicle transport | GO:0048489 | 4 | 15 | 0.00198371 | 0.017360226 |
| vocalization behavior | GO:0071625 | 4 | 15 | 0.00198371 | 0.017360226 |
| guanyl-nucleotide exchange factor activity | GO:0005085 | 11 | 123 | 0.00199335 | 0.017411859 |
| cerebral cortex development | GO:0021987 | 8 | 70 | 0.0020048 | 0.017478974 |
| dendritic spine | GO:0043197 | 13 | 162 | 0.00201335 | 0.017515085 |
| spermatogenesis | GO:0007283 | 24 | 404 | 0.00201648 | 0.017515085 |
| actin filament | GO:0005884 | 9 | 87 | 0.00202221 | 0.017532075 |
| Rab GTPase binding | GO:0017137 | 12 | 143 | 0.00209121 | 0.018096577 |
| alpha-tubulin binding | GO:0043014 | 6 | 40 | 0.00212806 | 0.01834709 |
| ovarian follicle development | GO:0001541 | 6 | 40 | 0.00212806 | 0.01834709 |
| regulation of insulin secretion | GO:0050796 | 8 | 71 | 0.00217456 | 0.018713328 |
| fibronectin binding | GO:0001968 | 5 | 27 | 0.00221261 | 0.018935535 |
| mRNA stabilization | GO:0048255 | 5 | 27 | 0.00221261 | 0.018935535 |
| carboxylic ester hydrolase activity | GO:0052689 | 5 | 27 | 0.00221261 | 0.018935535 |
| Wnt signaling pathway, planar cell polarity pathway | GO:0060071 | 9 | 89 | 0.0023312 | 0.019840804 |
| kinase activity | GO:0016301 | 9 | 89 | 0.0023312 | 0.019840804 |
| transcription coregulator activity | GO:0003712 | 9 | 89 | 0.0023312 | 0.019840804 |
| filopodium | GO:0030175 | 8 | 72 | 0.00235541 | 0.019973747 |
| negative regulation of cysteine-type endopeptidase activity involved in apoptotic process | GO:0043154 | 8 | 72 | 0.00235541 | 0.019973747 |
| response to starvation | GO:0042594 | 6 | 41 | 0.00238002 | 0.020068233 |
| regulation of cytosolic calcium ion concentration | GO:0051480 | 6 | 41 | 0.00238002 | 0.020068233 |
| clathrin-coated vesicle membrane | GO:0030665 | 7 | 56 | 0.00238768 | 0.020068233 |
| protein processing | GO:0016485 | 7 | 56 | 0.00238768 | 0.020068233 |
| positive regulation of nitric-oxide synthase biosynthetic process | GO:0051770 | 4 | 16 | 0.0024227 | 0.020068233 |
| positive regulation of sodium ion transport | GO:0010765 | 4 | 16 | 0.0024227 | 0.020068233 |
| dendrite self-avoidance | GO:0070593 | 4 | 16 | 0.0024227 | 0.020068233 |
| positive regulation of lipid biosynthetic process | GO:0046889 | 4 | 16 | 0.0024227 | 0.020068233 |
| extracellular matrix structural constituent conferring compression resistance | GO:0030021 | 4 | 16 | 0.0024227 | 0.020068233 |
| regulation of protein secretion | GO:0050708 | 4 | 16 | 0.0024227 | 0.020068233 |
| glycogen catabolic process | GO:0005980 | 4 | 16 | 0.0024227 | 0.020068233 |
| membrane depolarization during action potential | GO:0086010 | 4 | 16 | 0.0024227 | 0.020068233 |
| regulation of protein catabolic process | GO:0042176 | 4 | 16 | 0.0024227 | 0.020068233 |
| positive regulation of peptidyl-serine phosphorylation | GO:0033138 | 9 | 90 | 0.00249915 | 0.020513532 |
| recycling endosome | GO:0055037 | 11 | 127 | 0.00251238 | 0.020513532 |
| microfilament motor activity | GO:0000146 | 5 | 28 | 0.0025457 | 0.020513532 |
| positive regulation of viral genome replication | GO:0045070 | 5 | 28 | 0.0025457 | 0.020513532 |
| regulation of calcium ion transport | GO:0051924 | 5 | 28 | 0.0025457 | 0.020513532 |
| autophagosome | GO:0005776 | 8 | 73 | 0.00254784 | 0.020513532 |
| mRNA 3'-UTR binding | GO:0003730 | 8 | 73 | 0.00254784 | 0.020513532 |
| endoplasmic reticulum lumen | GO:0005788 | 19 | 296 | 0.00255563 | 0.020513532 |
| nucleus localization | GO:0051647 | 3 | 7 | 0.00256474 | 0.020513532 |
| regulation of synaptic vesicle fusion to presynaptic active zone membrane | GO:0031630 | 3 | 7 | 0.00256474 | 0.020513532 |
| cuprous ion binding | GO:1903136 | 3 | 7 | 0.00256474 | 0.020513532 |
| negative regulation of cytoplasmic translation | GO:2000766 | 3 | 7 | 0.00256474 | 0.020513532 |
| clustering of voltage-gated sodium channels | GO:0045162 | 3 | 7 | 0.00256474 | 0.020513532 |
| vacuole organization | GO:0007033 | 3 | 7 | 0.00256474 | 0.020513532 |
| creatine metabolic process | GO:0006600 | 3 | 7 | 0.00256474 | 0.020513532 |
| paranodal junction assembly | GO:0030913 | 3 | 7 | 0.00256474 | 0.020513532 |
| axon extension involved in axon guidance | GO:0048846 | 3 | 7 | 0.00256474 | 0.020513532 |
| response to manganese ion | GO:0010042 | 3 | 7 | 0.00256474 | 0.020513532 |
| CDP-choline pathway | GO:0006657 | 3 | 7 | 0.00256474 | 0.020513532 |
| cellular response to iron ion | GO:0071281 | 3 | 7 | 0.00256474 | 0.020513532 |
| lysosome organization | GO:0007040 | 6 | 42 | 0.00265376 | 0.021152707 |
| negative regulation of cell cycle | GO:0045786 | 6 | 42 | 0.00265376 | 0.021152707 |
| specific granule membrane | GO:0035579 | 9 | 91 | 0.00267656 | 0.021261462 |
| endosomal transport | GO:0016197 | 9 | 91 | 0.00267656 | 0.021261462 |
| structural molecule activity | GO:0005198 | 13 | 168 | 0.002704 | 0.021442839 |
| protein C-terminus binding | GO:0008022 | 14 | 189 | 0.00275064 | 0.021775474 |
| DNA helicase activity | GO:0003678 | 7 | 58 | 0.00285734 | 0.022555886 |
| transport vesicle | GO:0030133 | 9 | 92 | 0.00286378 | 0.022555886 |
| secretory granule | GO:0030141 | 9 | 92 | 0.00286378 | 0.022555886 |
| replication fork processing | GO:0031297 | 5 | 29 | 0.00291369 | 0.022722921 |
| regulation of Rho protein signal transduction | GO:0035023 | 5 | 29 | 0.00291369 | 0.022722921 |
| cortical actin cytoskeleton organization | GO:0030866 | 5 | 29 | 0.00291369 | 0.022722921 |
| neuron cell-cell adhesion | GO:0007158 | 4 | 17 | 0.0029241 | 0.022722921 |
| trans-Golgi network transport vesicle | GO:0030140 | 4 | 17 | 0.0029241 | 0.022722921 |
| retinoic acid receptor signaling pathway | GO:0048384 | 4 | 17 | 0.0029241 | 0.022722921 |
| determination of adult lifespan | GO:0008340 | 4 | 17 | 0.0029241 | 0.022722921 |
| negative regulation of anoikis | GO:2000811 | 4 | 17 | 0.0029241 | 0.022722921 |
| eye development | GO:0001654 | 6 | 43 | 0.00295044 | 0.022851126 |
| cellular response to epidermal growth factor stimulus | GO:0071364 | 6 | 43 | 0.00295044 | 0.022851126 |
| cell adhesion molecule binding | GO:0050839 | 7 | 59 | 0.00311717 | 0.024022378 |
| RNA polymerase II activating transcription factor binding | GO:0001102 | 7 | 59 | 0.00311717 | 0.024022378 |
| cellular response to growth factor stimulus | GO:0071363 | 7 | 59 | 0.00311717 | 0.024022378 |
| neuromuscular junction | GO:0031594 | 8 | 76 | 0.0031996 | 0.024616756 |
| signaling receptor activity | GO:0038023 | 15 | 214 | 0.00323613 | 0.024856703 |
| small GTPase mediated signal transduction | GO:0007264 | 9 | 94 | 0.00326917 | 0.024961036 |
| platelet aggregation | GO:0070527 | 6 | 44 | 0.0032712 | 0.024961036 |
| response to cytokine | GO:0034097 | 6 | 44 | 0.0032712 | 0.024961036 |
| positive regulation of protein secretion | GO:0050714 | 6 | 44 | 0.0032712 | 0.024961036 |
| positive regulation of vasoconstriction | GO:0045907 | 5 | 30 | 0.0033186 | 0.025042314 |
| regulation of synaptic transmission, glutamatergic | GO:0051966 | 5 | 30 | 0.0033186 | 0.025042314 |
| regulation of cytoskeleton organization | GO:0051493 | 5 | 30 | 0.0033186 | 0.025042314 |
| pattern specification process | GO:0007389 | 5 | 30 | 0.0033186 | 0.025042314 |
| response to tumor necrosis factor | GO:0034612 | 5 | 30 | 0.0033186 | 0.025042314 |
| protein targeting to mitochondrion | GO:0006626 | 5 | 30 | 0.0033186 | 0.025042314 |
| nucleotide binding | GO:0000166 | 7 | 60 | 0.00339466 | 0.025042314 |
| axon terminus | GO:0043679 | 7 | 60 | 0.00339466 | 0.025042314 |
| cellular iron ion homeostasis | GO:0006879 | 7 | 60 | 0.00339466 | 0.025042314 |
| positive regulation of osteoblast differentiation | GO:0045669 | 7 | 60 | 0.00339466 | 0.025042314 |
| negative regulation of protein binding | GO:0032091 | 8 | 77 | 0.00344339 | 0.025042314 |
| TORC1 complex | GO:0031931 | 3 | 8 | 0.00345019 | 0.025042314 |
| cellular hyperosmotic response | GO:0071474 | 3 | 8 | 0.00345019 | 0.025042314 |
| positive regulation of histone H3-K9 acetylation | GO:2000617 | 3 | 8 | 0.00345019 | 0.025042314 |
| hemidesmosome | GO:0030056 | 3 | 8 | 0.00345019 | 0.025042314 |
| plasma membrane to endosome transport | GO:0048227 | 3 | 8 | 0.00345019 | 0.025042314 |
| regulation of keratinocyte proliferation | GO:0010837 | 3 | 8 | 0.00345019 | 0.025042314 |
| cellular response to oxygen-glucose deprivation | GO:0090650 | 3 | 8 | 0.00345019 | 0.025042314 |
| regulation of calcium ion transmembrane transport via high voltage-gated calcium channel | GO:1902514 | 3 | 8 | 0.00345019 | 0.025042314 |
| TORC1 signaling | GO:0038202 | 3 | 8 | 0.00345019 | 0.025042314 |
| atrial septum development | GO:0003283 | 3 | 8 | 0.00345019 | 0.025042314 |
| negative regulation of RIG-I signaling pathway | GO:0039536 | 3 | 8 | 0.00345019 | 0.025042314 |
| anterograde axonal protein transport | GO:0099641 | 3 | 8 | 0.00345019 | 0.025042314 |
| vesicle-mediated transport in synapse | GO:0099003 | 3 | 8 | 0.00345019 | 0.025042314 |
| neuronal dense core vesicle | GO:0098992 | 3 | 8 | 0.00345019 | 0.025042314 |
| peripheral nervous system myelin maintenance | GO:0032287 | 3 | 8 | 0.00345019 | 0.025042314 |
| potassium channel inhibitor activity | GO:0019870 | 3 | 8 | 0.00345019 | 0.025042314 |
| regulation of histone H3-K4 methylation | GO:0051569 | 3 | 8 | 0.00345019 | 0.025042314 |
| histone H3-K14 acetylation | GO:0044154 | 3 | 8 | 0.00345019 | 0.025042314 |
| cytokine-mediated signaling pathway | GO:0019221 | 18 | 282 | 0.00345139 | 0.025042314 |
| kidney development | GO:0001822 | 9 | 95 | 0.00348811 | 0.025042314 |
| cellular sodium ion homeostasis | GO:0006883 | 4 | 18 | 0.00349202 | 0.025042314 |
| clathrin coat assembly | GO:0048268 | 4 | 18 | 0.00349202 | 0.025042314 |
| interleukin-6-mediated signaling pathway | GO:0070102 | 4 | 18 | 0.00349202 | 0.025042314 |
| muscle fiber development | GO:0048747 | 4 | 18 | 0.00349202 | 0.025042314 |
| olfactory bulb development | GO:0021772 | 4 | 18 | 0.00349202 | 0.025042314 |
| response to iron ion | GO:0010039 | 4 | 18 | 0.00349202 | 0.025042314 |
| filopodium membrane | GO:0031527 | 4 | 18 | 0.00349202 | 0.025042314 |
| regulation of microtubule polymerization or depolymerization | GO:0031110 | 4 | 18 | 0.00349202 | 0.025042314 |
| secretory granule lumen | GO:0034774 | 10 | 114 | 0.00356137 | 0.025500256 |
| ATPase-coupled transmembrane transporter activity | GO:0042626 | 6 | 45 | 0.00361723 | 0.025702215 |
| response to organic cyclic compound | GO:0014070 | 6 | 45 | 0.00361723 | 0.025702215 |
| negative regulation of I-kappaB kinase/NF-kappaB signaling | GO:0043124 | 6 | 45 | 0.00361723 | 0.025702215 |
| tau protein binding | GO:0048156 | 6 | 45 | 0.00361723 | 0.025702215 |
| anatomical structure development | GO:0048856 | 6 | 45 | 0.00361723 | 0.025702215 |
| MAPK cascade | GO:0000165 | 17 | 261 | 0.00364149 | 0.025835138 |
| cell-matrix adhesion | GO:0007160 | 9 | 96 | 0.00371839 | 0.026327494 |
| actomyosin structure organization | GO:0031032 | 5 | 31 | 0.00376242 | 0.026327494 |
| thyroid hormone receptor binding | GO:0046966 | 5 | 31 | 0.00376242 | 0.026327494 |
| growth factor binding | GO:0019838 | 5 | 31 | 0.00376242 | 0.026327494 |
| maintenance of blood-brain barrier | GO:0035633 | 5 | 31 | 0.00376242 | 0.026327494 |
| negative regulation of endothelial cell proliferation | GO:0001937 | 5 | 31 | 0.00376242 | 0.026327494 |
| embryonic skeletal system development | GO:0048706 | 5 | 31 | 0.00376242 | 0.026327494 |
| anchored component of plasma membrane | GO:0046658 | 5 | 31 | 0.00376242 | 0.026327494 |
| negative regulation of epithelial to mesenchymal transition | GO:0010719 | 5 | 31 | 0.00376242 | 0.026327494 |
| apical plasma membrane | GO:0016324 | 21 | 354 | 0.00376755 | 0.026327494 |
| protein deubiquitination | GO:0016579 | 17 | 262 | 0.00377514 | 0.026340921 |
| cellular response to leukemia inhibitory factor | GO:1990830 | 9 | 97 | 0.00396041 | 0.027548411 |
| ubiquitin binding | GO:0043130 | 8 | 79 | 0.00397395 | 0.027548411 |
| negative regulation of translation | GO:0017148 | 8 | 79 | 0.00397395 | 0.027548411 |
| voltage-gated ion channel activity | GO:0005244 | 6 | 46 | 0.00398969 | 0.027548411 |
| negative regulation of fat cell differentiation | GO:0045599 | 6 | 46 | 0.00398969 | 0.027548411 |
| forebrain development | GO:0030900 | 6 | 46 | 0.00398969 | 0.027548411 |
| interleukin-12-mediated signaling pathway | GO:0035722 | 6 | 46 | 0.00398969 | 0.027548411 |
| endosome membrane | GO:0010008 | 15 | 220 | 0.00411523 | 0.028309942 |
| cytoskeletal anchor activity | GO:0008093 | 4 | 19 | 0.00413044 | 0.028309942 |
| vesicle transport along actin filament | GO:0030050 | 4 | 19 | 0.00413044 | 0.028309942 |
| apicolateral plasma membrane | GO:0016327 | 4 | 19 | 0.00413044 | 0.028309942 |
| glycogen biosynthetic process | GO:0005978 | 4 | 19 | 0.00413044 | 0.028309942 |
| antigen processing and presentation of exogenous peptide antigen via MHC class II | GO:0019886 | 9 | 98 | 0.00421457 | 0.028770613 |
| intermediate filament | GO:0005882 | 10 | 117 | 0.00422294 | 0.028770613 |
| blood vessel remodeling | GO:0001974 | 5 | 32 | 0.00424718 | 0.028770613 |
| negative regulation of proteasomal ubiquitin-dependent protein catabolic process | GO:0032435 | 5 | 32 | 0.00424718 | 0.028770613 |
| 14-3-3 protein binding | GO:0071889 | 5 | 32 | 0.00424718 | 0.028770613 |
| microtubule associated complex | GO:0005875 | 5 | 32 | 0.00424718 | 0.028770613 |
| activation of protein kinase B activity | GO:0032148 | 5 | 32 | 0.00424718 | 0.028770613 |
| mismatch repair | GO:0006298 | 5 | 32 | 0.00424718 | 0.028770613 |
| JNK cascade | GO:0007254 | 6 | 47 | 0.00438977 | 0.029478706 |
| heterophilic cell-cell adhesion via plasma membrane cell adhesion molecules | GO:0007157 | 6 | 47 | 0.00438977 | 0.029478706 |
| negative regulation of cold-induced thermogenesis | GO:0120163 | 6 | 47 | 0.00438977 | 0.029478706 |
| negative regulation of Wnt signaling pathway | GO:0030178 | 6 | 47 | 0.00438977 | 0.029478706 |
| cell leading edge | GO:0031252 | 6 | 47 | 0.00438977 | 0.029478706 |
| bone development | GO:0060348 | 6 | 47 | 0.00438977 | 0.029478706 |
| peptidyl-tyrosine dephosphorylation | GO:0035335 | 9 | 99 | 0.00448128 | 0.029541424 |
| T-tubule organization | GO:0033292 | 3 | 9 | 0.00450082 | 0.029541424 |
| neurofilament | GO:0005883 | 3 | 9 | 0.00450082 | 0.029541424 |
| atrial cardiac muscle cell action potential | GO:0086014 | 3 | 9 | 0.00450082 | 0.029541424 |
| peptidyl-tyrosine dephosphorylation involved in inactivation of protein kinase activity | GO:1990264 | 3 | 9 | 0.00450082 | 0.029541424 |
| cellular response to parathyroid hormone stimulus | GO:0071374 | 3 | 9 | 0.00450082 | 0.029541424 |
| neurotransmitter receptor transport, endosome to postsynaptic membrane | GO:0098887 | 3 | 9 | 0.00450082 | 0.029541424 |
| actin filament reorganization | GO:0090527 | 3 | 9 | 0.00450082 | 0.029541424 |
| progesterone receptor signaling pathway | GO:0050847 | 3 | 9 | 0.00450082 | 0.029541424 |
| neuronal action potential propagation | GO:0019227 | 3 | 9 | 0.00450082 | 0.029541424 |
| pericardium development | GO:0060039 | 3 | 9 | 0.00450082 | 0.029541424 |
| positive regulation of synapse maturation | GO:0090129 | 3 | 9 | 0.00450082 | 0.029541424 |
| dendritic growth cone | GO:0044294 | 3 | 9 | 0.00450082 | 0.029541424 |
| iron ion transmembrane transporter activity | GO:0005381 | 3 | 9 | 0.00450082 | 0.029541424 |
| iris morphogenesis | GO:0061072 | 3 | 9 | 0.00450082 | 0.029541424 |
| negative regulation of sodium ion transmembrane transporter activity | GO:2000650 | 3 | 9 | 0.00450082 | 0.029541424 |
| cell morphogenesis | GO:0000902 | 8 | 81 | 0.00456543 | 0.029923179 |
| cell cycle | GO:0007049 | 16 | 245 | 0.00458964 | 0.030039528 |
| hippocampus development | GO:0021766 | 7 | 64 | 0.00469655 | 0.030695991 |
| ion transport | GO:0006811 | 9 | 100 | 0.00476095 | 0.030989871 |
| protein tyrosine phosphatase activity | GO:0004725 | 9 | 100 | 0.00476095 | 0.030989871 |
| regulation of heart contraction | GO:0008016 | 5 | 33 | 0.00477485 | 0.030989871 |
| positive regulation of axonogenesis | GO:0050772 | 5 | 33 | 0.00477485 | 0.030989871 |
| heterotrimeric G-protein complex | GO:0005834 | 5 | 33 | 0.00477485 | 0.030989871 |
| mitotic spindle organization | GO:0007052 | 6 | 48 | 0.00481866 | 0.031043306 |
| ankyrin binding | GO:0030506 | 4 | 20 | 0.00484321 | 0.031043306 |
| branched-chain amino acid catabolic process | GO:0009083 | 4 | 20 | 0.00484321 | 0.031043306 |
| histone-lysine N-methyltransferase activity | GO:0018024 | 4 | 20 | 0.00484321 | 0.031043306 |
| regulation of myelination | GO:0031641 | 4 | 20 | 0.00484321 | 0.031043306 |
| regulation of ventricular cardiac muscle cell membrane repolarization | GO:0060307 | 4 | 20 | 0.00484321 | 0.031043306 |
| inclusion body | GO:0016234 | 4 | 20 | 0.00484321 | 0.031043306 |
| axonal fasciculation | GO:0007413 | 4 | 20 | 0.00484321 | 0.031043306 |
| negative regulation of adenylate cyclase activity | GO:0007194 | 4 | 20 | 0.00484321 | 0.031043306 |
| midbody | GO:0030496 | 12 | 160 | 0.00487406 | 0.03114177 |
| RNA splicing | GO:0008380 | 12 | 160 | 0.00487406 | 0.03114177 |
| positive regulation of catalytic activity | GO:0043085 | 8 | 82 | 0.00488538 | 0.03114177 |
| memory | GO:0007613 | 8 | 82 | 0.00488538 | 0.03114177 |
| cellular response to DNA damage stimulus | GO:0006974 | 16 | 248 | 0.00511409 | 0.032555029 |
| phosphatidylinositol-4,5-bisphosphate binding | GO:0005546 | 8 | 83 | 0.00522222 | 0.033152509 |
| regulation of protein stability | GO:0031647 | 8 | 83 | 0.00522222 | 0.033152509 |
| protein destabilization | GO:0031648 | 6 | 49 | 0.00527755 | 0.033458068 |
| protein stabilization | GO:0050821 | 13 | 183 | 0.00530287 | 0.03357281 |
| dendrite morphogenesis | GO:0048813 | 5 | 34 | 0.00534742 | 0.033762868 |
| protein monoubiquitination | GO:0006513 | 5 | 34 | 0.00534742 | 0.033762868 |
| chromatin remodeling | GO:0006338 | 9 | 102 | 0.00536081 | 0.033801459 |
| positive regulation of NIK/NF-kappaB signaling | GO:1901224 | 7 | 66 | 0.00547353 | 0.034418787 |
| regulation of blood pressure | GO:0008217 | 7 | 66 | 0.00547353 | 0.034418787 |
| cell cortex | GO:0005938 | 11 | 142 | 0.00550618 | 0.03454959 |
| transmembrane transport | GO:0055085 | 18 | 296 | 0.0055128 | 0.03454959 |
| regulation of transcription, DNA-templated | GO:0006355 | 37 | 773 | 0.00555866 | 0.03454959 |
| retrograde vesicle-mediated transport, Golgi to endoplasmic reticulum | GO:0006890 | 8 | 84 | 0.0055765 | 0.03454959 |
| heparin binding | GO:0008201 | 12 | 163 | 0.00558272 | 0.03454959 |
| cell migration involved in sprouting angiogenesis | GO:0002042 | 4 | 21 | 0.00563406 | 0.03454959 |
| mRNA splice site selection | GO:0006376 | 4 | 21 | 0.00563406 | 0.03454959 |
| voltage-gated sodium channel activity | GO:0005248 | 4 | 21 | 0.00563406 | 0.03454959 |
| regulation of nitric-oxide synthase activity | GO:0050999 | 4 | 21 | 0.00563406 | 0.03454959 |
| non-canonical Wnt signaling pathway | GO:0035567 | 4 | 21 | 0.00563406 | 0.03454959 |
| vesicle docking | GO:0048278 | 4 | 21 | 0.00563406 | 0.03454959 |
| phosphatidylinositol 3-kinase binding | GO:0043548 | 4 | 21 | 0.00563406 | 0.03454959 |
| cellular protein-containing complex localization | GO:0034629 | 3 | 10 | 0.00572481 | 0.03454959 |
| positive regulation of protein kinase C signaling | GO:0090037 | 3 | 10 | 0.00572481 | 0.03454959 |
| negative regulation of potassium ion transmembrane transport | GO:1901380 | 3 | 10 | 0.00572481 | 0.03454959 |
| prostate gland development | GO:0030850 | 3 | 10 | 0.00572481 | 0.03454959 |
| plasma membrane protein complex | GO:0098797 | 3 | 10 | 0.00572481 | 0.03454959 |
| histone methyltransferase activity (H3-K9 specific) | GO:0046974 | 3 | 10 | 0.00572481 | 0.03454959 |
| negative regulation of cell size | GO:0045792 | 3 | 10 | 0.00572481 | 0.03454959 |
| neuron projection maintenance | GO:1990535 | 3 | 10 | 0.00572481 | 0.03454959 |
| muscle structure development | GO:0061061 | 3 | 10 | 0.00572481 | 0.03454959 |
| glutamate binding | GO:0016595 | 3 | 10 | 0.00572481 | 0.03454959 |
| cellular response to leucine | GO:0071233 | 3 | 10 | 0.00572481 | 0.03454959 |
| positive regulation of transcription of nucleolar large rRNA by RNA polymerase I | GO:1901838 | 3 | 10 | 0.00572481 | 0.03454959 |
| positive regulation of mitochondrial depolarization | GO:0051901 | 3 | 10 | 0.00572481 | 0.03454959 |
| phospholipase C activity | GO:0004629 | 3 | 10 | 0.00572481 | 0.03454959 |
| regulation of dendritic spine development | GO:0060998 | 3 | 10 | 0.00572481 | 0.03454959 |
| juxtaparanode region of axon | GO:0044224 | 3 | 10 | 0.00572481 | 0.03454959 |
| fructose metabolic process | GO:0006000 | 3 | 10 | 0.00572481 | 0.03454959 |
| divalent metal ion transport | GO:0070838 | 3 | 10 | 0.00572481 | 0.03454959 |
| histone H3-K9 methylation | GO:0051567 | 3 | 10 | 0.00572481 | 0.03454959 |
| basal plasma membrane | GO:0009925 | 6 | 50 | 0.00576762 | 0.034628068 |
| E-box binding | GO:0070888 | 6 | 50 | 0.00576762 | 0.034628068 |
| regulation of synaptic plasticity | GO:0048167 | 6 | 50 | 0.00576762 | 0.034628068 |
| positive regulation of protein serine/threonine kinase activity | GO:0071902 | 6 | 50 | 0.00576762 | 0.034628068 |
| ficolin-1-rich granule lumen | GO:1904813 | 10 | 123 | 0.00583462 | 0.034985148 |
| BMP signaling pathway | GO:0030509 | 8 | 85 | 0.0059488 | 0.035457639 |
| voltage-gated potassium channel complex | GO:0008076 | 8 | 85 | 0.0059488 | 0.035457639 |
| regulation of endocytosis | GO:0030100 | 5 | 35 | 0.00596683 | 0.035457639 |
| positive regulation of epithelial cell migration | GO:0010634 | 5 | 35 | 0.00596683 | 0.035457639 |
| negative regulation of extrinsic apoptotic signaling pathway | GO:2001237 | 5 | 35 | 0.00596683 | 0.035457639 |
| cell-cell junction assembly | GO:0007043 | 5 | 35 | 0.00596683 | 0.035457639 |
| calcium channel regulator activity | GO:0005246 | 5 | 35 | 0.00596683 | 0.035457639 |
| response to virus | GO:0009615 | 9 | 104 | 0.00601751 | 0.035713138 |
| endomembrane system | GO:0012505 | 10 | 125 | 0.00646683 | 0.038330794 |
| negative regulation of Rho protein signal transduction | GO:0035024 | 4 | 22 | 0.00650654 | 0.038419187 |
| cell-cell adhesion mediator activity | GO:0098632 | 4 | 22 | 0.00650654 | 0.038419187 |
| branching morphogenesis of an epithelial tube | GO:0048754 | 4 | 22 | 0.00650654 | 0.038419187 |
| protein autoubiquitination | GO:0051865 | 7 | 69 | 0.00681432 | 0.040134542 |
| regulation of hematopoietic stem cell differentiation | GO:1902036 | 7 | 69 | 0.00681432 | 0.040134542 |
| positive regulation of neuron apoptotic process | GO:0043525 | 6 | 52 | 0.00684605 | 0.040270398 |
| protease binding | GO:0002020 | 9 | 107 | 0.00711663 | 0.041359663 |
| arachidonate-CoA ligase activity | GO:0047676 | 3 | 11 | 0.00712913 | 0.041359663 |
| secondary heart field specification | GO:0003139 | 3 | 11 | 0.00712913 | 0.041359663 |
| positive regulation of transcription by RNA polymerase III | GO:0045945 | 3 | 11 | 0.00712913 | 0.041359663 |
| mesodermal cell differentiation | GO:0048333 | 3 | 11 | 0.00712913 | 0.041359663 |
| vinculin binding | GO:0017166 | 3 | 11 | 0.00712913 | 0.041359663 |
| regulation of cell-cell adhesion | GO:0022407 | 3 | 11 | 0.00712913 | 0.041359663 |
| positive regulation of vascular permeability | GO:0043117 | 3 | 11 | 0.00712913 | 0.041359663 |
| SNARE complex assembly | GO:0035493 | 3 | 11 | 0.00712913 | 0.041359663 |
| positive regulation of extrinsic apoptotic signaling pathway in absence of ligand | GO:2001241 | 3 | 11 | 0.00712913 | 0.041359663 |
| cellular response to forskolin | GO:1904322 | 3 | 11 | 0.00712913 | 0.041359663 |
| ATPase binding | GO:0051117 | 8 | 88 | 0.00717951 | 0.041599979 |
| glycosaminoglycan biosynthetic process | GO:0006024 | 5 | 37 | 0.00735384 | 0.042189268 |
| cell maturation | GO:0048469 | 5 | 37 | 0.00735384 | 0.042189268 |
| ureteric bud development | GO:0001657 | 5 | 37 | 0.00735384 | 0.042189268 |
| anion transmembrane transport | GO:0098656 | 5 | 37 | 0.00735384 | 0.042189268 |
| Wnt signaling pathway, calcium modulating pathway | GO:0007223 | 5 | 37 | 0.00735384 | 0.042189268 |
| chloride transport | GO:0006821 | 5 | 37 | 0.00735384 | 0.042189268 |
| synaptic vesicle endocytosis | GO:0048488 | 5 | 37 | 0.00735384 | 0.042189268 |
| negative regulation of protein-containing complex assembly | GO:0031333 | 5 | 37 | 0.00735384 | 0.042189268 |
| regulation of cardiac conduction | GO:1903779 | 6 | 53 | 0.00743678 | 0.042612446 |
| melanosome organization | GO:0032438 | 4 | 23 | 0.00746407 | 0.042663639 |
| fibroblast growth factor binding | GO:0017134 | 4 | 23 | 0.00746407 | 0.042663639 |
| nuclear membrane | GO:0031965 | 15 | 237 | 0.0077054 | 0.043988911 |
| protein-containing complex assembly | GO:0065003 | 10 | 129 | 0.00788974 | 0.044986055 |
| positive regulation of MAPK cascade | GO:0043410 | 9 | 109 | 0.00793048 | 0.045107612 |
| presynapse | GO:0098793 | 9 | 109 | 0.00793048 | 0.045107612 |
| sequence-specific DNA binding | GO:0043565 | 22 | 405 | 0.0080019 | 0.045458219 |
| calcium channel activity | GO:0005262 | 6 | 54 | 0.00806339 | 0.045695818 |
| response to mechanical stimulus | GO:0009612 | 6 | 54 | 0.00806339 | 0.045695818 |
| dendritic shaft | GO:0043198 | 5 | 38 | 0.00812519 | 0.04587818 |
| protein kinase B signaling | GO:0043491 | 5 | 38 | 0.00812519 | 0.04587818 |
| positive regulation of neuron death | GO:1901216 | 5 | 38 | 0.00812519 | 0.04587818 |
| Notch signaling pathway | GO:0007219 | 9 | 110 | 0.00836304 | 0.047163873 |
| double-stranded RNA binding | GO:0003725 | 7 | 72 | 0.00838461 | 0.047228242 |
| protein-lysine N-methyltransferase activity | GO:0016279 | 4 | 24 | 0.00850988 | 0.047655251 |
| calyx of Held | GO:0044305 | 4 | 24 | 0.00850988 | 0.047655251 |
| translation | GO:0006412 | 12 | 173 | 0.00855221 | 0.047655251 |
| negative regulation of angiogenesis | GO:0016525 | 8 | 91 | 0.00859299 | 0.047655251 |
| paranode region of axon | GO:0033270 | 3 | 12 | 0.00871972 | 0.047655251 |
| protein localization to synapse | GO:0035418 | 3 | 12 | 0.00871972 | 0.047655251 |
| planar cell polarity pathway involved in neural tube closure | GO:0090179 | 3 | 12 | 0.00871972 | 0.047655251 |
| cellular response to hydroxyurea | GO:0072711 | 3 | 12 | 0.00871972 | 0.047655251 |
| positive regulation of nuclear-transcribed mRNA poly(A) tail shortening | GO:0060213 | 3 | 12 | 0.00871972 | 0.047655251 |
| negative regulation of cell motility | GO:2000146 | 3 | 12 | 0.00871972 | 0.047655251 |
| neurotransmitter uptake | GO:0001504 | 3 | 12 | 0.00871972 | 0.047655251 |
| L-type voltage-gated calcium channel complex | GO:1990454 | 3 | 12 | 0.00871972 | 0.047655251 |
| regulation of postsynaptic neurotransmitter receptor internalization | GO:0099149 | 3 | 12 | 0.00871972 | 0.047655251 |
| semaphorin receptor activity | GO:0017154 | 3 | 12 | 0.00871972 | 0.047655251 |
| positive regulation of positive chemotaxis | GO:0050927 | 3 | 12 | 0.00871972 | 0.047655251 |
| presynaptic active zone membrane | GO:0048787 | 3 | 12 | 0.00871972 | 0.047655251 |
| filamin binding | GO:0031005 | 3 | 12 | 0.00871972 | 0.047655251 |
| dermatan sulfate biosynthetic process | GO:0030208 | 3 | 12 | 0.00871972 | 0.047655251 |
| peroxisome proliferator activated receptor binding | GO:0042975 | 3 | 12 | 0.00871972 | 0.047655251 |
| striatum development | GO:0021756 | 3 | 12 | 0.00871972 | 0.047655251 |
| positive regulation of protein localization to plasma membrane | GO:1903078 | 6 | 55 | 0.00872705 | 0.047655251 |
| transcription repressor complex | GO:0017053 | 6 | 55 | 0.00872705 | 0.047655251 |
| cis-regulatory region sequence-specific DNA binding | GO:0000987 | 6 | 55 | 0.00872705 | 0.047655251 |
| response to calcium ion | GO:0051592 | 6 | 55 | 0.00872705 | 0.047655251 |
| extracellular vesicle | GO:1903561 | 6 | 55 | 0.00872705 | 0.047655251 |
| copper ion binding | GO:0005507 | 6 | 55 | 0.00872705 | 0.047655251 |
| mitochondrial membrane | GO:0031966 | 9 | 111 | 0.00881328 | 0.048069605 |
| positive regulation of nitric oxide biosynthetic process | GO:0045429 | 5 | 39 | 0.00895086 | 0.048486528 |
| negative regulation of signal transduction | GO:0009968 | 5 | 39 | 0.00895086 | 0.048486528 |
| NADH dehydrogenase (ubiquinone) activity | GO:0008137 | 5 | 39 | 0.00895086 | 0.048486528 |
| phosphatidylinositol-3,4,5-trisphosphate binding | GO:0005547 | 5 | 39 | 0.00895086 | 0.048486528 |
| DNA duplex unwinding | GO:0032508 | 7 | 73 | 0.00896276 | 0.048486528 |
| Ras protein signal transduction | GO:0007265 | 7 | 73 | 0.00896276 | 0.048486528 |
| cytoplasmic stress granule | GO:0010494 | 7 | 73 | 0.00896276 | 0.048486528 |
