# Supplementary material for: Phenotypic and Transcriptomic Analysis Revealed a Lack of Risk Perception by Native Tadpoles Toward Novel Non‐Native Fish
Source: Ecol Evol. 2024 Oct 21;14(10):e70481. doi: 10.1002/ece3.70481 (PMC11493475; doi:10.1002/ece3.70481)
Supplement: Supplementary file 7 — Table S6. [file ECE3-14-e70481-s002.docx]

**Table_S6_SuppInfo.** Enriched GO terms of DEGs in the “*C. auratus* treatment - Muscle *vs* Control - Muscle” comparison.

| Term | ID | Input number | Background number | P-Value | Corrected P-Value |
| --- | --- | --- | --- | --- | --- |
| calcium activated cation channel activity | GO:0005227 | 2 | 14 | 2.37E-05 | 0.002971111 |
| cellular response to peptide | GO:1901653 | 2 | 15 | 2.69E-05 | 0.002971111 |
| intracellular receptor signaling pathway | GO:0030522 | 2 | 26 | 7.45E-05 | 0.005488868 |
| stereocilium membrane | GO:0060171 | 1 | 5 | 0.002747775 | 0.023598057 |
| cellular response to corticotropin-releasing hormone stimulus | GO:0071376 | 1 | 5 | 0.002747775 | 0.023598057 |
| maintenance of animal organ identity | GO:0048496 | 1 | 5 | 0.002747775 | 0.023598057 |
| positive regulation of retinoic acid receptor signaling pathway | GO:0048386 | 1 | 5 | 0.002747775 | 0.023598057 |
| positive regulation of cholesterol biosynthetic process | GO:0045542 | 1 | 7 | 0.003662115 | 0.023598057 |
| cellular protein metabolic process | GO:0044267 | 2 | 198 | 0.003743772 | 0.023598057 |
| regulation of fatty acid metabolic process | GO:0019217 | 1 | 8 | 0.004118987 | 0.023598057 |
| integral component of membrane | GO:0016021 | 6 | 3643 | 0.004455974 | 0.023598057 |
| positive regulation of protein metabolic process | GO:0051247 | 1 | 10 | 0.005032139 | 0.023598057 |
| nucleoside metabolic process | GO:0009116 | 1 | 12 | 0.0059445 | 0.023598057 |
| alpha-linolenic acid metabolic process | GO:0036109 | 1 | 13 | 0.006400385 | 0.023598057 |
| positive regulation of triglyceride biosynthetic process | GO:0010867 | 1 | 13 | 0.006400385 | 0.023598057 |
| very long-chain fatty acid metabolic process | GO:0000038 | 1 | 16 | 0.007766853 | 0.023598057 |
| nucleoside diphosphate phosphorylation | GO:0006165 | 1 | 17 | 0.008221948 | 0.023598057 |
| estrogen metabolic process | GO:0008210 | 1 | 20 | 0.00958605 | 0.026104926 |
| bile acid biosynthetic process | GO:0006699 | 1 | 27 | 0.012762066 | 0.030656703 |
| regulation of cholesterol biosynthetic process | GO:0045540 | 1 | 35 | 0.016380021 | 0.03731943 |
| fatty acid beta-oxidation | GO:0006635 | 1 | 43 | 0.019985451 | 0.03908659 |
