# Supplementary material for: Phenotypic and Transcriptomic Analysis Revealed a Lack of Risk Perception by Native Tadpoles Toward Novel Non‐Native Fish
Source: Ecol Evol. 2024 Oct 21;14(10):e70481. doi: 10.1002/ece3.70481 (PMC11493475; doi:10.1002/ece3.70481)
Supplement: Supplementary file 8 — Table S7. [file ECE3-14-e70481-s006.docx]

**Table_S7_SuppInfo.** Enriched KEGG pathways of DEGs in the “*S. prenanti* treatment - Liver *vs* Control - Liver” comparison.

| Term | ID | Input number | Background number | P-Value | Corrected P-Value |
| --- | --- | --- | --- | --- | --- |
| Metabolic pathways | hsa01100 | 83 | 1433 | 1.62E-17 | 4.77E-15 |
| Lysosome | hsa04142 | 21 | 123 | 6.94E-13 | 1.02E-10 |
| Ribosome | hsa03010 | 17 | 153 | 3.76E-08 | 3.70E-06 |
| Regulation of actin cytoskeleton | hsa04810 | 17 | 214 | 2.92E-06 | 0.000215 |
| Protein processing in endoplasmic reticulum | hsa04141 | 14 | 165 | 1.08E-05 | 0.000638 |
| Autophagy - animal | hsa04140 | 12 | 128 | 1.83E-05 | 0.000805 |
| Focal adhesion | hsa04510 | 15 | 199 | 1.96E-05 | 0.000805 |
| mTOR signaling pathway | hsa04150 | 13 | 153 | 2.18E-05 | 0.000805 |
| Spliceosome | hsa03040 | 12 | 135 | 2.98E-05 | 0.000976 |
| Insulin signaling pathway | hsa04910 | 12 | 137 | 3.40E-05 | 0.001004 |
| Fatty acid biosynthesis | hsa00061 | 5 | 18 | 6.57E-05 | 0.001763 |
| Sphingolipid metabolism | hsa00600 | 7 | 47 | 7.68E-05 | 0.001887 |
| Proteoglycans in cancer | hsa05205 | 14 | 203 | 9.02E-05 | 0.002047 |
| PI3K-Akt signaling pathway | hsa04151 | 19 | 354 | 0.000135 | 0.002841 |
| Ubiquitin mediated proteolysis | hsa04120 | 11 | 137 | 0.000147 | 0.00289 |
| Biosynthesis of amino acids | hsa01230 | 8 | 75 | 0.000201 | 0.003589 |
| Thyroid hormone signaling pathway | hsa04919 | 10 | 119 | 0.000207 | 0.003589 |
| AMPK signaling pathway | hsa04152 | 10 | 120 | 0.00022 | 0.00361 |
| Cellular senescence | hsa04218 | 11 | 160 | 0.000511 | 0.007896 |
| Pathways in cancer | hsa05200 | 23 | 530 | 0.000535 | 0.007896 |
| Longevity regulating pathway | hsa04211 | 8 | 89 | 0.000582 | 0.008174 |
| Cysteine and methionine metabolism | hsa00270 | 6 | 49 | 0.000648 | 0.008331 |
| RNA transport | hsa03013 | 11 | 165 | 0.00065 | 0.008331 |
| p53 signaling pathway | hsa04115 | 7 | 72 | 0.00083 | 0.010208 |
| Dilated cardiomyopathy (DCM) | hsa05414 | 8 | 96 | 0.000922 | 0.010885 |
| Adrenergic signaling in cardiomyocytes | hsa04261 | 10 | 149 | 0.001075 | 0.012194 |
| Platelet activation | hsa04611 | 9 | 124 | 0.001131 | 0.012267 |
| Human papillomavirus infection | hsa05165 | 16 | 330 | 0.001234 | 0.012267 |
| Phagosome | hsa04145 | 10 | 152 | 0.001238 | 0.012267 |
| Arginine biosynthesis | hsa00220 | 4 | 21 | 0.001247 | 0.012267 |
| Fatty acid metabolism | hsa01212 | 6 | 57 | 0.001333 | 0.012689 |
| cAMP signaling pathway | hsa04024 | 12 | 214 | 0.001558 | 0.013992 |
| Endocytosis | hsa04144 | 13 | 244 | 0.001565 | 0.013992 |
| Glycerolipid metabolism | hsa00561 | 6 | 61 | 0.001836 | 0.015929 |
| ECM-receptor interaction | hsa04512 | 7 | 86 | 0.002156 | 0.01817 |
| Leukocyte transendothelial migration | hsa04670 | 8 | 112 | 0.002305 | 0.018507 |
| Fluid shear stress and atherosclerosis | hsa05418 | 9 | 139 | 0.002365 | 0.018507 |
| cGMP-PKG signaling pathway | hsa04022 | 10 | 167 | 0.002384 | 0.018507 |
| Signaling pathways regulating pluripotency of stem cells | hsa04550 | 9 | 140 | 0.002475 | 0.018721 |
| Tight junction | hsa04530 | 10 | 170 | 0.002692 | 0.019228 |
| Basal transcription factors | hsa03022 | 5 | 45 | 0.002699 | 0.019228 |
| Hypertrophic cardiomyopathy (HCM) | hsa05410 | 7 | 90 | 0.002738 | 0.019228 |
| Viral carcinogenesis | hsa05203 | 11 | 201 | 0.002871 | 0.019694 |
| TGF-beta signaling pathway | hsa04350 | 7 | 94 | 0.003433 | 0.022311 |
| Fc gamma R-mediated phagocytosis | hsa04666 | 7 | 94 | 0.003433 | 0.022311 |
| Epithelial cell signaling in Helicobacter pylori infection | hsa05120 | 6 | 70 | 0.003479 | 0.022311 |
| Adherens junction | hsa04520 | 6 | 72 | 0.003958 | 0.024809 |
| Glycerophospholipid metabolism | hsa00564 | 7 | 97 | 0.004037 | 0.024809 |
| Glioma | hsa05214 | 6 | 75 | 0.004766 | 0.028693 |
| PPAR signaling pathway | hsa03320 | 6 | 76 | 0.00506 | 0.029856 |
| Arrhythmogenic right ventricular cardiomyopathy (ARVC) | hsa05412 | 6 | 77 | 0.005368 | 0.030627 |
| Purine metabolism | hsa00230 | 8 | 130 | 0.005399 | 0.030627 |
| Complement and coagulation cascades | hsa04610 | 6 | 79 | 0.006024 | 0.033528 |
| Calcium signaling pathway | hsa04020 | 10 | 193 | 0.006256 | 0.033604 |
| Glucagon signaling pathway | hsa04922 | 7 | 106 | 0.006336 | 0.033604 |
| Glutathione metabolism | hsa00480 | 5 | 56 | 0.006379 | 0.033604 |
| Alanine, aspartate and glutamate metabolism | hsa00250 | 4 | 36 | 0.00718 | 0.037081 |
| Other glycan degradation | hsa00511 | 3 | 18 | 0.007291 | 0.037081 |
| Cholinergic synapse | hsa04725 | 7 | 112 | 0.008335 | 0.041007 |
| ErbB signaling pathway | hsa04012 | 6 | 85 | 0.00834 | 0.041007 |
| Toxoplasmosis | hsa05145 | 7 | 113 | 0.008708 | 0.041117 |
| Cardiac muscle contraction | hsa04260 | 6 | 86 | 0.008781 | 0.041117 |
| Insulin secretion | hsa04911 | 6 | 86 | 0.008781 | 0.041117 |
| Pyruvate metabolism | hsa00620 | 4 | 39 | 0.009268 | 0.042721 |
| Longevity regulating pathway - multiple species | hsa04213 | 5 | 62 | 0.00943 | 0.042799 |
| Breast cancer | hsa05224 | 8 | 147 | 0.01057 | 0.047245 |
| mRNA surveillance pathway | hsa03015 | 6 | 91 | 0.011236 | 0.049471 |
| Oxytocin signaling pathway | hsa04921 | 8 | 153 | 0.013068 | 0.055409 |
| Proximal tubule bicarbonate reclamation | hsa04964 | 3 | 23 | 0.013276 | 0.055409 |
| Amoebiasis | hsa05146 | 6 | 95 | 0.013521 | 0.055409 |
| Fatty acid degradation | hsa00071 | 4 | 44 | 0.013557 | 0.055409 |
| Carbohydrate digestion and absorption | hsa04973 | 4 | 44 | 0.013557 | 0.055409 |
| Transcriptional misregulation in cancer | hsa05202 | 9 | 186 | 0.013711 | 0.055409 |
| Cushing syndrome | hsa04934 | 8 | 155 | 0.013989 | 0.055491 |
| Adipocytokine signaling pathway | hsa04920 | 5 | 69 | 0.014108 | 0.055491 |
| Endocrine resistance | hsa01522 | 6 | 98 | 0.015435 | 0.059912 |
| Phosphatidylinositol signaling system | hsa04070 | 6 | 99 | 0.016112 | 0.060762 |
| Choline metabolism in cancer | hsa05231 | 6 | 99 | 0.016112 | 0.060762 |
| Wnt signaling pathway | hsa04310 | 8 | 160 | 0.016498 | 0.060762 |
| Ether lipid metabolism | hsa00565 | 4 | 47 | 0.016647 | 0.060762 |
| MAPK signaling pathway | hsa04010 | 12 | 295 | 0.016684 | 0.060762 |
| Necroptosis | hsa04217 | 8 | 162 | 0.017588 | 0.063147 |
| Valine, leucine and isoleucine degradation | hsa00280 | 4 | 48 | 0.017767 | 0.063147 |
| Inositol phosphate metabolism | hsa00562 | 5 | 74 | 0.018267 | 0.063398 |
| Bacterial invasion of epithelial cells | hsa05100 | 5 | 74 | 0.018267 | 0.063398 |
| Thermogenesis | hsa04714 | 10 | 231 | 0.019062 | 0.065388 |
| Apoptosis | hsa04210 | 7 | 136 | 0.021084 | 0.070008 |
| Antigen processing and presentation | hsa04612 | 5 | 77 | 0.021113 | 0.070008 |
| Epstein-Barr virus infection | hsa05169 | 9 | 201 | 0.021121 | 0.070008 |
| Nicotinate and nicotinamide metabolism | hsa00760 | 3 | 30 | 0.025239 | 0.082729 |
| B cell receptor signaling pathway | hsa04662 | 5 | 82 | 0.026471 | 0.085812 |
| Pathogenic Escherichia coli infection | hsa05130 | 4 | 55 | 0.026912 | 0.086293 |
| Human immunodeficiency virus 1 infection | hsa05170 | 9 | 212 | 0.028134 | 0.089244 |
| Cell adhesion molecules (CAMs) | hsa04514 | 7 | 146 | 0.029099 | 0.091322 |
| Autophagy - other | hsa04136 | 3 | 32 | 0.029439 | 0.091415 |
| Pyrimidine metabolism | hsa00240 | 4 | 57 | 0.029955 | 0.092049 |
| Axon guidance | hsa04360 | 8 | 181 | 0.03062 | 0.093122 |
| Sphingolipid signaling pathway | hsa04071 | 6 | 119 | 0.034239 | 0.103067 |
| GABAergic synapse | hsa04727 | 5 | 89 | 0.035315 | 0.105233 |
| Prion diseases | hsa05020 | 3 | 35 | 0.036384 | 0.107333 |
| Chemokine signaling pathway | hsa04062 | 8 | 190 | 0.038649 | 0.111748 |
| Starch and sucrose metabolism | hsa00500 | 3 | 36 | 0.038869 | 0.111748 |
| MicroRNAs in cancer | hsa05206 | 11 | 299 | 0.039017 | 0.111748 |
| Arachidonic acid metabolism | hsa00590 | 4 | 63 | 0.04026 | 0.1142 |
| Small cell lung cancer | hsa05222 | 5 | 93 | 0.041097 | 0.114374 |
| GnRH signaling pathway | hsa04912 | 5 | 93 | 0.041097 | 0.114374 |
| Prostate cancer | hsa05215 | 5 | 97 | 0.047419 | 0.130734 |
| Pancreatic secretion | hsa04972 | 5 | 98 | 0.049084 | 0.131935 |
| Aldosterone synthesis and secretion | hsa04925 | 5 | 98 | 0.049084 | 0.131935 |
| Ferroptosis | hsa04216 | 3 | 40 | 0.049643 | 0.131935 |
| Glycine, serine and threonine metabolism | hsa00260 | 3 | 40 | 0.049643 | 0.131935 |
