# Supplementary material for: Phenotypic and Transcriptomic Analysis Revealed a Lack of Risk Perception by Native Tadpoles Toward Novel Non‐Native Fish
Source: Ecol Evol. 2024 Oct 21;14(10):e70481. doi: 10.1002/ece3.70481 (PMC11493475; doi:10.1002/ece3.70481)
Supplement: Supplementary file 9 — Table S8. [file ECE3-14-e70481-s009.docx]

**Table_S8_SuppInfo.** Enriched KEGG pathways of DEGs in the “*C. auratus* treatment - Liver *vs* Control - Liver” comparison.

| Term | ID | Input number | Background number | P-Value | Corrected P-Value |
| --- | --- | --- | --- | --- | --- |
| PI3K-Akt signaling pathway | hsa04151 | 11 | 354 | 1.27E-06 | 0.00019 |
| ECM-receptor interaction | hsa04512 | 6 | 86 | 4.72E-06 | 0.00036 |
| Amoebiasis | hsa05146 | 6 | 95 | 8.10E-06 | 0.00041 |
| Metabolic pathways | hsa01100 | 19 | 1433 | 5.04E-05 | 0.00193 |
| Hypertrophic cardiomyopathy (HCM) | hsa05410 | 5 | 90 | 8.43E-05 | 0.00215 |
| Small cell lung cancer | hsa05222 | 5 | 93 | 9.77E-05 | 0.00215 |
| Nitrogen metabolism | hsa00910 | 3 | 17 | 0.000106 | 0.00215 |
| Dilated cardiomyopathy (DCM) | hsa05414 | 5 | 96 | 0.000113 | 0.00215 |
| Human papillomavirus infection | hsa05165 | 8 | 330 | 0.000196 | 0.00333 |
| Focal adhesion | hsa04510 | 6 | 199 | 0.000411 | 0.00629 |
| Biosynthesis of amino acids | hsa01230 | 4 | 75 | 0.000514 | 0.00715 |
| Signaling pathways regulating pluripotency of stem cells | hsa04550 | 5 | 140 | 0.000601 | 0.00767 |
| mTOR signaling pathway | hsa04150 | 5 | 153 | 0.000885 | 0.01042 |
| Pathways in cancer | hsa05200 | 9 | 530 | 0.000996 | 0.01088 |
| Cholesterol metabolism | hsa04979 | 3 | 50 | 0.001943 | 0.01982 |
| Autophagy - animal | hsa04140 | 4 | 128 | 0.00342 | 0.0327 |
| 2-Oxocarboxylic acid metabolism | hsa01210 | 2 | 18 | 0.003853 | 0.03468 |
| Pantothenate and CoA biosynthesis | hsa00770 | 2 | 19 | 0.004246 | 0.03609 |
| One carbon pool by folate | hsa00670 | 2 | 20 | 0.004656 | 0.03749 |
| Neuroactive ligand-receptor interaction | hsa04080 | 6 | 338 | 0.00559 | 0.04012 |
| Mannose type O-glycan biosynthesis | hsa00515 | 2 | 23 | 0.005992 | 0.04012 |
| Proximal tubule bicarbonate reclamation | hsa04964 | 2 | 23 | 0.005992 | 0.04012 |
| Arrhythmogenic right ventricular cardiomyopathy (ARVC) | hsa05412 | 3 | 77 | 0.006219 | 0.04012 |
| Endocytosis | hsa04144 | 5 | 244 | 0.006293 | 0.04012 |
| Complement and coagulation cascades | hsa04610 | 3 | 79 | 0.006659 | 0.04075 |
| Protein digestion and absorption | hsa04974 | 3 | 90 | 0.009402 | 0.05533 |
| Prion diseases | hsa05020 | 2 | 35 | 0.012827 | 0.07269 |
| Alanine, aspartate and glutamate metabolism | hsa00250 | 2 | 36 | 0.013498 | 0.07376 |
| Aldosterone-regulated sodium reabsorption | hsa04960 | 2 | 37 | 0.014185 | 0.07484 |
| Toxoplasmosis | hsa05145 | 3 | 113 | 0.017001 | 0.08429 |
| Fat digestion and absorption | hsa04975 | 2 | 41 | 0.017079 | 0.08429 |
| Type I diabetes mellitus | hsa04940 | 2 | 43 | 0.018611 | 0.08899 |
| Valine, leucine and isoleucine degradation | hsa00280 | 2 | 48 | 0.022685 | 0.10518 |
| Cysteine and methionine metabolism | hsa00270 | 2 | 49 | 0.02354 | 0.10593 |
| Ras signaling pathway | hsa04014 | 4 | 232 | 0.024663 | 0.10781 |
| Systemic lupus erythematosus | hsa05322 | 3 | 133 | 0.025727 | 0.10934 |
| Gastric cancer | hsa05226 | 3 | 149 | 0.034138 | 0.13745 |
| Non-alcoholic fatty liver disease (NAFLD) | hsa04932 | 3 | 149 | 0.034138 | 0.13745 |
| Basal cell carcinoma | hsa05217 | 2 | 63 | 0.036822 | 0.14445 |
| Staphylococcus aureus infection | hsa05150 | 2 | 68 | 0.042117 | 0.1611 |
| Hepatocellular carcinoma | hsa05225 | 3 | 168 | 0.045747 | 0.17071 |
