# Supplementary material for: Phenotypic and Transcriptomic Analysis Revealed a Lack of Risk Perception by Native Tadpoles Toward Novel Non‐Native Fish
Source: Ecol Evol. 2024 Oct 21;14(10):e70481. doi: 10.1002/ece3.70481 (PMC11493475; doi:10.1002/ece3.70481)
Supplement: Supplementary file 10 — Table S9. [file ECE3-14-e70481-s010.docx]

**Table_S9_SuppInfo.** Enriched KEGG pathways of DEGs in the “*S. prenanti* treatment - Muscle *vs* Control - Muscle” comparison.

| Term | ID | Input number | Background number | P-Value | Corrected P-Value |
| --- | --- | --- | --- | --- | --- |
| Metabolic pathways | hsa01100 | 96 | 1433 | 2.39E-12 | 3.98E-10 |
| Proteoglycans in cancer | hsa05205 | 31 | 203 | 2.75E-12 | 3.98E-10 |
| MAPK signaling pathway | hsa04010 | 34 | 295 | 2.53E-10 | 2.44E-08 |
| Oxytocin signaling pathway | hsa04921 | 24 | 153 | 4.82E-10 | 3.49E-08 |
| Focal adhesion | hsa04510 | 26 | 199 | 3.07E-09 | 1.56E-07 |
| Pathways in cancer | hsa05200 | 45 | 530 | 3.22E-09 | 1.56E-07 |
| Insulin signaling pathway | hsa04910 | 21 | 137 | 8.30E-09 | 3.44E-07 |
| mTOR signaling pathway | hsa04150 | 22 | 153 | 1.05E-08 | 3.80E-07 |
| PI3K-Akt signaling pathway | hsa04151 | 34 | 354 | 1.75E-08 | 5.64E-07 |
| Human cytomegalovirus infection | hsa05163 | 25 | 225 | 1.09E-07 | 3.18E-06 |
| AMPK signaling pathway | hsa04152 | 18 | 120 | 1.27E-07 | 3.35E-06 |
| Relaxin signaling pathway | hsa04926 | 18 | 130 | 3.69E-07 | 8.37E-06 |
| Longevity regulating pathway | hsa04211 | 15 | 89 | 3.75E-07 | 8.37E-06 |
| cGMP-PKG signaling pathway | hsa04022 | 20 | 167 | 6.90E-07 | 1.43E-05 |
| HIF-1 signaling pathway | hsa04066 | 16 | 109 | 8.12E-07 | 1.57E-05 |
| Endocytosis | hsa04144 | 24 | 244 | 1.45E-06 | 2.45E-05 |
| Wnt signaling pathway | hsa04310 | 19 | 160 | 1.46E-06 | 2.45E-05 |
| Rap1 signaling pathway | hsa04015 | 22 | 210 | 1.52E-06 | 2.45E-05 |
| Human papillomavirus infection | hsa05165 | 28 | 330 | 2.94E-06 | 4.49E-05 |
| Platelet activation | hsa04611 | 16 | 124 | 3.70E-06 | 5.37E-05 |
| Cholinergic synapse | hsa04725 | 15 | 112 | 4.91E-06 | 6.78E-05 |
| Axon guidance | hsa04360 | 19 | 181 | 7.51E-06 | 9.90E-05 |
| Adrenergic signaling in cardiomyocytes | hsa04261 | 17 | 149 | 8.30E-06 | 0.000104 |
| Longevity regulating pathway - multiple species | hsa04213 | 11 | 62 | 8.64E-06 | 0.000104 |
| Thyroid hormone signaling pathway | hsa04919 | 15 | 119 | 9.52E-06 | 0.00011 |
| Hypertrophic cardiomyopathy (HCM) | hsa05410 | 13 | 90 | 1.00E-05 | 0.000112 |
| Kaposi sarcoma-associated herpesvirus infection | hsa05167 | 19 | 186 | 1.07E-05 | 0.000114 |
| Arrhythmogenic right ventricular cardiomyopathy (ARVC) | hsa05412 | 12 | 77 | 1.10E-05 | 0.000114 |
| Apelin signaling pathway | hsa04371 | 16 | 137 | 1.16E-05 | 0.000116 |
| EGFR tyrosine kinase inhibitor resistance | hsa01521 | 12 | 79 | 1.39E-05 | 0.000132 |
| Insulin resistance | hsa04931 | 14 | 108 | 1.41E-05 | 0.000132 |
| Dilated cardiomyopathy (DCM) | hsa05414 | 13 | 96 | 1.86E-05 | 0.000169 |
| Ras signaling pathway | hsa04014 | 21 | 232 | 2.04E-05 | 0.000178 |
| Autophagy - animal | hsa04140 | 15 | 128 | 2.09E-05 | 0.000178 |
| Choline metabolism in cancer | hsa05231 | 13 | 99 | 2.50E-05 | 0.000207 |
| Retrograde endocannabinoid signaling | hsa04723 | 16 | 148 | 2.77E-05 | 0.000223 |
| GABAergic synapse | hsa04727 | 12 | 89 | 4.02E-05 | 0.000315 |
| Hippo signaling pathway | hsa04390 | 16 | 154 | 4.31E-05 | 0.000329 |
| Signaling pathways regulating pluripotency of stem cells | hsa04550 | 15 | 140 | 5.37E-05 | 0.000395 |
| Calcium signaling pathway | hsa04020 | 18 | 193 | 5.44E-05 | 0.000395 |
| Human immunodeficiency virus 1 infection | hsa05170 | 19 | 212 | 5.62E-05 | 0.000397 |
| Regulation of actin cytoskeleton | hsa04810 | 19 | 214 | 6.31E-05 | 0.000435 |
| Cell adhesion molecules (CAMs) | hsa04514 | 15 | 146 | 8.29E-05 | 0.000559 |
| Toxoplasmosis | hsa05145 | 13 | 113 | 8.63E-05 | 0.000569 |
| Endocrine resistance | hsa01522 | 12 | 98 | 9.34E-05 | 0.00059 |
| Glutamatergic synapse | hsa04724 | 13 | 114 | 9.36E-05 | 0.00059 |
| Dopaminergic synapse | hsa04728 | 14 | 131 | 9.65E-05 | 0.000596 |
| ErbB signaling pathway | hsa04012 | 11 | 85 | 0.000116 | 0.000703 |
| Tight junction | hsa04530 | 16 | 170 | 0.000126 | 0.000741 |
| Insulin secretion | hsa04911 | 11 | 86 | 0.000128 | 0.000741 |
| Thermogenesis | hsa04714 | 19 | 231 | 0.000159 | 0.000906 |
| Fluid shear stress and atherosclerosis | hsa05418 | 14 | 139 | 0.000171 | 0.000936 |
| Bacterial invasion of epithelial cells | hsa05100 | 10 | 74 | 0.000171 | 0.000936 |
| Basal cell carcinoma | hsa05217 | 9 | 63 | 0.000247 | 0.001327 |
| Phospholipase D signaling pathway | hsa04072 | 14 | 148 | 0.00031 | 0.001633 |
| Gastric cancer | hsa05226 | 14 | 149 | 0.00033 | 0.001686 |
| Circadian entrainment | hsa04713 | 11 | 97 | 0.000331 | 0.001686 |
| Hepatocellular carcinoma | hsa05225 | 15 | 168 | 0.00034 | 0.001701 |
| Aldosterone synthesis and secretion | hsa04925 | 11 | 98 | 0.000359 | 0.001764 |
| Inflammatory mediator regulation of TRP channels | hsa04750 | 11 | 100 | 0.00042 | 0.00203 |
| Central carbon metabolism in cancer | hsa05230 | 9 | 69 | 0.000453 | 0.002088 |
| Renal cell carcinoma | hsa05211 | 9 | 69 | 0.000453 | 0.002088 |
| Melanogenesis | hsa04916 | 11 | 101 | 0.000454 | 0.002088 |
| Cushing syndrome | hsa04934 | 14 | 155 | 0.000476 | 0.002156 |
| Pathogenic Escherichia coli infection | hsa05130 | 8 | 55 | 0.000486 | 0.002168 |
| Estrogen signaling pathway | hsa04915 | 13 | 138 | 0.000519 | 0.002282 |
| Yersinia infection | hsa05135 | 12 | 121 | 0.000553 | 0.002393 |
| Adherens junction | hsa04520 | 9 | 72 | 0.0006 | 0.002527 |
| Gap junction | hsa04540 | 10 | 88 | 0.000601 | 0.002527 |
| Cellular senescence | hsa04218 | 14 | 160 | 0.000636 | 0.002637 |
| Glucagon signaling pathway | hsa04922 | 11 | 106 | 0.000657 | 0.002685 |
| Vasopressin-regulated water reabsorption | hsa04962 | 7 | 44 | 0.000676 | 0.002723 |
| VEGF signaling pathway | hsa04370 | 8 | 59 | 0.000739 | 0.002934 |
| PPAR signaling pathway | hsa03320 | 9 | 76 | 0.000853 | 0.003343 |
| TGF-beta signaling pathway | hsa04350 | 10 | 94 | 0.000958 | 0.003705 |
| Purine metabolism | hsa00230 | 12 | 130 | 0.000989 | 0.003752 |
| Non-alcoholic fatty liver disease (NAFLD) | hsa04932 | 13 | 149 | 0.001004 | 0.003752 |
| Synaptic vesicle cycle | hsa04721 | 9 | 78 | 0.001009 | 0.003752 |
| Vascular smooth muscle contraction | hsa04270 | 12 | 132 | 0.001117 | 0.004048 |
| FoxO signaling pathway | hsa04068 | 12 | 132 | 0.001117 | 0.004048 |
| cAMP signaling pathway | hsa04024 | 16 | 214 | 0.001305 | 0.004618 |
| Mitophagy - animal | hsa04137 | 8 | 65 | 0.001306 | 0.004618 |
| Progesterone-mediated oocyte maturation | hsa04914 | 10 | 99 | 0.001374 | 0.0048 |
| Amyotrophic lateral sclerosis (ALS) | hsa05014 | 7 | 51 | 0.001471 | 0.004993 |
| AGE-RAGE signaling pathway in diabetic complications | hsa04933 | 10 | 100 | 0.001472 | 0.004993 |
| Aldosterone-regulated sodium reabsorption | hsa04960 | 6 | 37 | 0.001492 | 0.004993 |
| Ubiquitin mediated proteolysis | hsa04120 | 12 | 137 | 0.001498 | 0.004993 |
| Chagas disease (American trypanosomiasis) | hsa05142 | 10 | 103 | 0.001803 | 0.005942 |
| Adipocytokine signaling pathway | hsa04920 | 8 | 69 | 0.001846 | 0.006014 |
| C-type lectin receptor signaling pathway | hsa04625 | 10 | 104 | 0.001926 | 0.006205 |
| Hepatitis B | hsa05161 | 13 | 163 | 0.002117 | 0.006747 |
| Regulation of lipolysis in adipocytes | hsa04923 | 7 | 55 | 0.002176 | 0.006832 |
| Parathyroid hormone synthesis, secretion and action | hsa04928 | 10 | 106 | 0.002191 | 0.006832 |
| PD-L1 expression and PD-1 checkpoint pathway in cancer | hsa05235 | 9 | 89 | 0.002331 | 0.007126 |
| Th17 cell differentiation | hsa04659 | 10 | 107 | 0.002334 | 0.007126 |
| Transcriptional misregulation in cancer | hsa05202 | 14 | 186 | 0.002401 | 0.007255 |
| Breast cancer | hsa05224 | 12 | 147 | 0.002584 | 0.007726 |
| Fatty acid metabolism | hsa01212 | 7 | 57 | 0.002614 | 0.007737 |
| Pancreatic cancer | hsa05212 | 8 | 75 | 0.002969 | 0.008526 |
| Gastric acid secretion | hsa04971 | 8 | 75 | 0.002969 | 0.008526 |
| Glioma | hsa05214 | 8 | 75 | 0.002969 | 0.008526 |
| Fc gamma R-mediated phagocytosis | hsa04666 | 9 | 94 | 0.003269 | 0.009295 |
| ABC transporters | hsa02010 | 6 | 45 | 0.003617 | 0.010184 |
| Type II diabetes mellitus | hsa04930 | 6 | 46 | 0.00399 | 0.011125 |
| Hedgehog signaling pathway | hsa04340 | 6 | 47 | 0.00439 | 0.012124 |
| Phosphatidylinositol signaling system | hsa04070 | 9 | 99 | 0.004481 | 0.01226 |
| Neurotrophin signaling pathway | hsa04722 | 10 | 119 | 0.004715 | 0.01278 |
| Salmonella infection | hsa05132 | 8 | 83 | 0.005222 | 0.014023 |
| SNARE interactions in vesicular transport | hsa04130 | 5 | 34 | 0.005347 | 0.014227 |
| Acute myeloid leukemia | hsa05221 | 7 | 66 | 0.005474 | 0.01443 |
| Colorectal cancer | hsa05210 | 8 | 86 | 0.00634 | 0.016563 |
| Chemokine signaling pathway | hsa04062 | 13 | 190 | 0.00706 | 0.018281 |
| Osteoclast differentiation | hsa04380 | 10 | 128 | 0.007513 | 0.019282 |
| Platinum drug resistance | hsa01524 | 7 | 73 | 0.008963 | 0.0228 |
| TNF signaling pathway | hsa04668 | 9 | 112 | 0.009282 | 0.023406 |
| Inositol phosphate metabolism | hsa00562 | 7 | 74 | 0.00957 | 0.023905 |
| GnRH signaling pathway | hsa04912 | 8 | 93 | 0.009645 | 0.023905 |
| Glycine, serine and threonine metabolism | hsa00260 | 5 | 40 | 0.009833 | 0.024165 |
| Bladder cancer | hsa05219 | 5 | 41 | 0.010772 | 0.026073 |
| Amoebiasis | hsa05146 | 8 | 95 | 0.010789 | 0.026073 |
| Lysine degradation | hsa00310 | 6 | 59 | 0.011775 | 0.028221 |
| Glycerophospholipid metabolism | hsa00564 | 8 | 97 | 0.012031 | 0.028597 |
| Pancreatic secretion | hsa04972 | 8 | 98 | 0.012689 | 0.029918 |
| Influenza A | hsa05164 | 11 | 167 | 0.016042 | 0.037517 |
| Huntington disease | hsa05016 | 12 | 193 | 0.018055 | 0.041887 |
| Circadian rhythm | hsa04710 | 4 | 31 | 0.018536 | 0.042663 |
| Non-small cell lung cancer | hsa05223 | 6 | 66 | 0.018788 | 0.042901 |
| Cardiac muscle contraction | hsa04260 | 7 | 86 | 0.019366 | 0.043537 |
| ECM-receptor interaction | hsa04512 | 7 | 86 | 0.019366 | 0.043537 |
| Oocyte meiosis | hsa04114 | 9 | 128 | 0.019598 | 0.043607 |
| Human T-cell leukemia virus 1 infection | hsa05166 | 13 | 219 | 0.019698 | 0.043607 |
| Fc epsilon RI signaling pathway | hsa04664 | 6 | 68 | 0.021223 | 0.046384 |
| Phosphonate and phosphinate metabolism | hsa00440 | 2 | 6 | 0.021273 | 0.046384 |
| Fatty acid biosynthesis | hsa00061 | 3 | 18 | 0.022387 | 0.048449 |
