# Supplementary material for: Phenotypic and Transcriptomic Analysis Revealed a Lack of Risk Perception by Native Tadpoles Toward Novel Non‐Native Fish
Source: Ecol Evol. 2024 Oct 21;14(10):e70481. doi: 10.1002/ece3.70481 (PMC11493475; doi:10.1002/ece3.70481)
Supplement: Supplementary file 11 — Table S10. [file ECE3-14-e70481-s003.docx]

**Table_S10_SuppInfo.** Enriched KEGG pathways of DEGs in the “*C. auratus* treatment - Muscle *vs* Control - Muscle” comparison.

| Term | ID | Input number | Background number | P-Value | Corrected P-Value |
| --- | --- | --- | --- | --- | --- |
| Apelin signaling pathway | hsa04371 | 2 | 137 | 0.00183 | 0.06604 |
| Primary bile acid biosynthesis | hsa00120 | 1 | 17 | 0.00822 | 0.08916 |
| Biosynthesis of unsaturated fatty acids | hsa01040 | 1 | 27 | 0.01276 | 0.08916 |
| Fatty acid metabolism | hsa01212 | 1 | 57 | 0.02626 | 0.08916 |
| Pyrimidine metabolism | hsa00240 | 1 | 57 | 0.02626 | 0.08916 |
| Viral myocarditis | hsa05416 | 1 | 60 | 0.02761 | 0.08916 |
| Cortisol synthesis and secretion | hsa04927 | 1 | 65 | 0.02984 | 0.08916 |
| Gastric acid secretion | hsa04971 | 1 | 75 | 0.03428 | 0.08916 |
| Arrhythmogenic right ventricular cardiomyopathy (ARVC) | hsa05412 | 1 | 77 | 0.03517 | 0.08916 |
| Drug metabolism - other enzymes | hsa00983 | 1 | 79 | 0.03606 | 0.08916 |
| Peroxisome | hsa04146 | 1 | 83 | 0.03783 | 0.08916 |
| Hypertrophic cardiomyopathy (HCM) | hsa05410 | 1 | 90 | 0.04092 | 0.08916 |
| mRNA surveillance pathway | hsa03015 | 1 | 91 | 0.04136 | 0.08916 |
| Dilated cardiomyopathy (DCM) | hsa05414 | 1 | 96 | 0.04356 | 0.08916 |
| Aldosterone synthesis and secretion | hsa04925 | 1 | 98 | 0.04444 | 0.08916 |
| Ribosome biogenesis in eukaryotes | hsa03008 | 1 | 105 | 0.04751 | 0.08916 |
| Insulin resistance | hsa04931 | 1 | 108 | 0.04882 | 0.08916 |
| AMPK signaling pathway | hsa04152 | 1 | 120 | 0.05406 | 0.08916 |
| Platelet activation | hsa04611 | 1 | 124 | 0.05579 | 0.08916 |
| Purine metabolism | hsa00230 | 1 | 130 | 0.0584 | 0.08916 |
| Vascular smooth muscle contraction | hsa04270 | 1 | 132 | 0.05926 | 0.08916 |
| FoxO signaling pathway | hsa04068 | 1 | 132 | 0.05926 | 0.08916 |
| Insulin signaling pathway | hsa04910 | 1 | 137 | 0.06142 | 0.08916 |
| Fluid shear stress and atherosclerosis | hsa05418 | 1 | 139 | 0.06229 | 0.08916 |
| Non-alcoholic fatty liver disease (NAFLD) | hsa04932 | 1 | 149 | 0.06659 | 0.08916 |
| Oxytocin signaling pathway | hsa04921 | 1 | 153 | 0.06831 | 0.08916 |
| Cushing syndrome | hsa04934 | 1 | 155 | 0.06917 | 0.08916 |
| RNA transport | hsa03013 | 1 | 165 | 0.07345 | 0.08916 |
| cGMP-PKG signaling pathway | hsa04022 | 1 | 167 | 0.0743 | 0.08916 |
| Influenza A | hsa05164 | 1 | 167 | 0.0743 | 0.08916 |
| Calcium signaling pathway | hsa04020 | 1 | 193 | 0.08532 | 0.09883 |
| Focal adhesion | hsa04510 | 1 | 199 | 0.08785 | 0.09883 |
| Regulation of actin cytoskeleton | hsa04810 | 1 | 214 | 0.09413 | 0.10269 |
| MAPK signaling pathway | hsa04010 | 1 | 295 | 0.12737 | 0.13487 |
| Metabolic pathways | hsa01100 | 2 | 1433 | 0.13903 | 0.143 |
| PI3K-Akt signaling pathway | hsa04151 | 1 | 354 | 0.15086 | 0.15086 |
